# Pentaphosphorylated magic spot nucleotides: chemoenzymatic synthesis and disassembly-based sensing†

**DOI:** 10.1039/d6ob00328a

**Published:** 2026-04-14

**Authors:** Patrick Moser, Subhra Kanti Roy, Marvin Herzog, Felix Bauer, Felix Wollensack, Bernhard Breit, Henning J. Jessen

**Affiliations:** a Institute of Organic Chemistry, Albert-Ludwigs-Universität Freiburg Albertstraße 21 79104 Freiburg im Breisgau Germany henning.jessen@oc.uni-freiburg.de; b CIBSS-Centre for Integrative Biological Signalling Studies, University of Freiburg 79104 Freiburg Germany

## Abstract

The magic spot nucleotides (MSNs), (p)ppGpp and (p)ppApp, play central roles in bacterial stress signaling, yet their selective detection and chemical accessibility remain limited. This work presents a scalable chemo-enzymatic synthesis of natural and functionalized pentaphosphorylated MSNs based on a cyclic pyrophosphoryl phosphoramidite (cPyPA) mediated phosphorylation and RNase T2-catalyzed hydrolysis. This approach enables preparative access to defined 3′-monophosphates (ppAp, ppGp) and the pentaphosphorylated products pppApp and pppGpp. In parallel, a metal–ligand disassembly-based fluorescence probe that operates in water was developed for the selective detection of MSNs. Coordination of the alarmone to an Fe(iii)-salen complex induces its demetallation and fluorescence activation through salicylaldehyde release, supported by theoretical and spectroscopic studies. The probe displays a two- to threefold selectivity for (p)ppGpp and (p)ppApp over other nucleotides and responds most strongly to MSNs bearing 3′- and 5′-pyrophosphate groups. The probe also detects enzymatically generated ppGpp from *Staphylococcus aureus* RelP reactions *in vitro*. This work combines a robust synthesis route for pentaphosphorylated MSNs with a readily accessible fluorescence sensor, thereby laying the foundation for future investigations into bacterial stress signaling.

## Abstract 2

A new synthesis of pentaphosphorylated magic spot nucleotides and their sensing based on a disassembly approach are presented.

## Graphical abstract

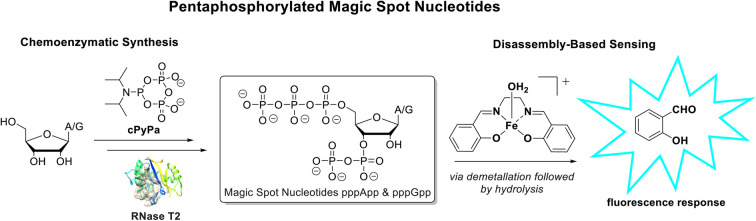

## Introduction

Hyperphosphorylated guanosine and adenosine nucleotides, (p)ppGpp and (p)ppApp – collectively referred to as magic spot nucleotides (MSN) – play a central role in bacterial stress signalling and adaptation.^1^ Under stress conditions such as amino acid deficiency, heat shock or oxidative stress, the enzymes RelA, SpoT and the small alarmone synthetases (SAS) synthesize (p)ppGpp by transferring pyrophosphate (PPi) from ATP to the 3′-hydroxy group of GDP or GTP.^2,3^ This process triggers the so-called stringent response, a conserved bacterial survival mechanism that leads to a comprehensive reprogramming of cellular metabolism. The accumulation of (p)ppGpp directly modulates RNA polymerase activity, thereby influencing the expression of numerous genes involved in metabolism, virulence and antibiotic resistance.^2–4^ Furthermore, (p)ppGpp is involved in the regulation of biofilm formation in *Enterococcus faecalis* and *Pseudomonas putida*, linking the stringent response to chronic infections and increased antibiotic tolerance.^5^ (p)ppGpp has also been identified in plants, where it controls transcription and translation in chloroplasts and cell nuclei and is involved in adaptation to environmental stress.^6^ In addition, adenosine-derived magic spot nucleotides such as (p)ppApp are emerging as additional regulatory messengers, although their biological functions remain underexplored.^7^

Given their central regulatory functions, access to (p)ppGpp and (p)ppApp is of great importance for understanding bacterial persistence and signalling pathways. The detection methods available to date include radiolabelling, thin-layer chromatography, HPLC-UV, LC-MS and CE-MS analysis.^8–10^ The accumulation of ppGpp in *Arabidopsis thaliana* has also been quantified using UPLC-ESI-qMS/MS.^11,12^ Although LC-MS-based methods offer higher sensitivity, they require complex instrumentation and are not well suited for real-time measurements.^8,13^ In recent years, colorimetric and fluorescence-based sensors have been developed; however, some of these systems suffer from synthetic complexity or poor stability in aqueous media.^14^

**Scheme 1:**
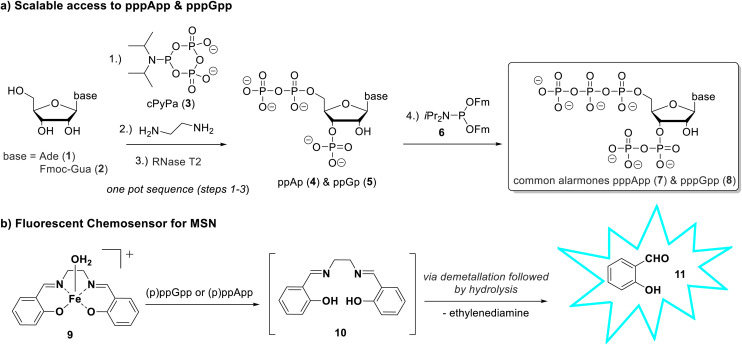
Overview of the synthesis and sensing strategy for MSNs. (a) cPyPA 3 is used to introduce three phosphates on nucleosides in a one-pot key transformation. This enables scalable access to defined 3′-monophosphates 4 and 5 as intermediates for the synthesis of native and modified pentaphosphorylated MSNs. (b) An Fe(iii)-salen complex 9 acts as a disassembly-based fluorescence probe for selective detection of (p)ppGpp and (p)ppApp *via* demetallation and release of fluorescent salicylaldehyde (11).

The efficient chemical synthesis of MSNs has also been a challenge. Classic phosphorylation strategies or enzymatic methods usually yield only milligram quantities of the highly charged nucleotides and can result in 2′/3′-regioisomer mixtures that are difficult to separate.^15^ To address these limitations, we developed a robust and scalable chemo-enzymatic route to the pentaphosphorylated MSNs pppGpp and pppApp. This is based on cPyPa-mediated phosphorylation, followed by ring opening with ethylenediamine and RNase T2-catalysed hydrolysis, which enables the regioselective formation of 3′-monophosphates (Scheme 1a).^16–18^ These intermediates can be further converted into canonical and functionalized pentaphosphorylated MSNs.

Building on this synthetic approach, we further developed a turn-on fluorescence sensor for the real-time detection of (p)ppGpp and (p)ppApp in water. Inspired by the so-called disassembly strategy for phosphate and pyrophosphate sensors, we investigated a probe based on a Fe(iii)-salen complex. Binding of the target analyte to the metal centre triggers disassembly of the complex, resulting in a fluorescence turn-on response (Scheme 1b).

Here, we report (i) a compact and scalable chemo-enzymatic synthesis of pentaphosphorylated magic spot nucleotides and (ii) the development of a disassembly-based Fe(iii)-salen fluorescence probe for their detection. Together, these advances provide an integrated chemical toolbox for studying bacterial stress signalling in biochemical and analytical applications.

## Results and discussion

### Scalable access to pppApp and ppGpp

Previous synthesis routes to pentaphosphorylated MSNs (pppNpp) were based on the nucleotide phosphorylation with cPyPa,^19^ in which propargylamine was used to open the cyclic intermediate 12.^16^ Although this approach allowed access to amino-functionalized pppNpp derivatives, the subsequent acidic cleavage of the corresponding phosphoramidate led to partial hydrolysis of the sensitive pentaphosphate. To overcome this limitation, we used ethylenediamine instead of propargylamine,^18^ an approach that has recently been introduced as an efficient method for opening cyclotriphosphate intermediates.^17^

**Scheme 2:**
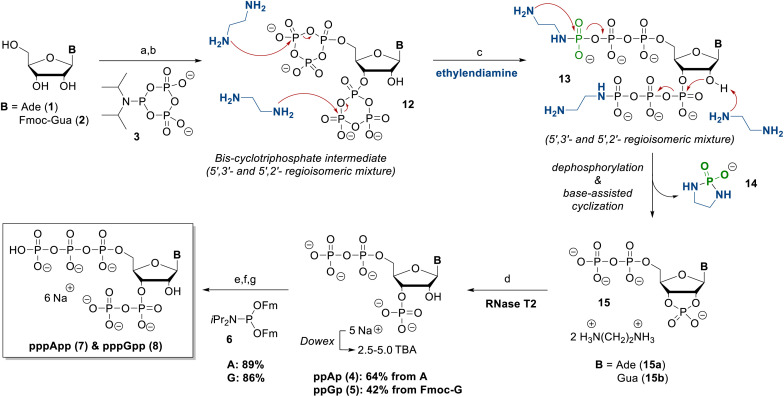
Chemoenzymatic synthesis of the MSNs pppApp and pppGpp from A and Fmoc-G. The route involves cPyPa-mediated phosphorylation and ethylenediamine-assisted opening of the cyclic intermediate 12, followed by RNase T2-catalysed hydrolysis and phosphoramidite coupling of ppNp to pppNpp. (a) 3 (5.0 equiv.), ETT (12 equiv.), DMF, rt, 45 min; (b) *m*CPBA (7.5 equiv.), 0 °C, 10 min; (c) ethylenediamine (150 equiv.); (d) RNase T2, H_2_O, 37 °C, 12 h; (e) 5 (1.7 equiv.), ETT (3.5 equiv.), DMF, rt, 15 min; (f) *m*CPBA (2.1 equiv.), 0 °C, 10 min; (g) DBU (10 vol%), rt, 30 min. Abbreviations: TBA: tetrabutylammonium, Fmoc: fluorenylmethoxycarbonyl, Fm: fluorenylmethyl, DMF: dimethylformamide, ETT: 5-(ethylthio)-1*H*-tetrazole, *m*CPBA: *meta*-chloroperbenzoic acid, DBU: diazabicycloundecene, cPyPa: cyclo-triphosphate amidite.

The reaction of adenosine and 2-*N*-(Fmoc)-guanosine (2) with an excess of cPyPa (3) in the presence of 5-(ethylthio)-1*H*-tetrazole (ETT), followed by oxidation with *m*CPBA, affords the corresponding bis-cyclotriphosphate intermediates 12 as a mixture of the 5′,2′- and 5′,3′-cyclotriphosphorylated isomers (Scheme 2). The Fmoc protecting group from the guanosine derivative had to be introduced beforehand due to the low solubility of guanosine in DMF.

Upon incubation with ethylenediamine, these intermediates undergo a sequence of linearization and intramolecular cyclization reactions, removing the γ-phosphate as a phosphorodiamidate 14.^16,17^ The amine acts as both a nucleophile and a base and also facilitates 5-*exo* cyclization to the corresponding 2′,3′-cyclophosphate intermediates. The large excess of amine also cleaves of the Fmoc protecting group from the guanosine derivative. The resulting 2′,3′-cyclophosphates 15 were hydrolysed overnight, catalysed by RNase T2 at 37 °C and pH 5.5, yielding exclusively the corresponding 3′-monophosphates ppAp (4) and ppGp (5) – in line with previous reports on ribonuclease-mediated hydrolysis of MSNs.^16,17^ After purification *via* SAX chromatography, ppAp (4) and ppGp (5) were isolated as their ammonium salts in 64% yield, and 42% yield, respectively.

Subsequent phosphorylation using Fm-P-amidite chemistry^20,21^ in the presence of ETT, followed by oxidation with *m*CPBA and Fm removal with DBU, yielded ppApp (7) and ppGpp (8) in isolated yields of 89% and 86%, respectively. The sequence discussed herein also only requires two isolation steps, as all other steps can be carried out in a one-flask reaction. Overall, this leads to a very efficient synthetic access to the pentaphosphorylated magic spot nucleotides pppGpp & pppApp.

**Scheme 3:**
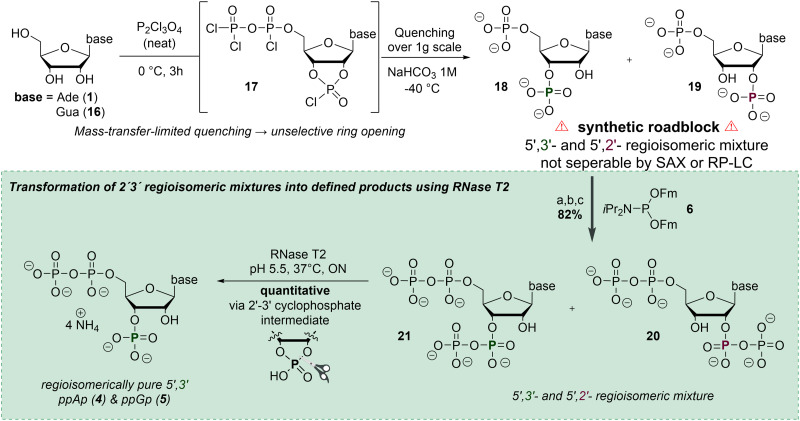
Phosphorylation of nucleosides on a gram-scale leads to unselective ring opening and inseparable 2′/3′-regioisomeric mixtures (top, synthetic roadblock). Subsequent conversion to ppNpp and RNase T2-mediated hydrolysis enable chemoenzymatic isomerisation *via* a transient 2′,3′-cyclophosphate intermediate, affording regioisomerically pure 3′-ppNp (bottom). (a) 6 (1.7 equiv.), ETT (3.5 equiv.), DMF, rt, 15 min; (b) *m*CPBA (2.1 equiv.), 0 °C, 10 min; (c) DBU (10 vol%), rt, 30 min. Abbreviations: Fm: fluorenylmethyl, DMF: dimethylformamide, ETT: 5-(ethylthio)-1*H*-tetrazole, *m*CPBA: *meta*-chloroperbenzoic acid, DBU: diazabicycloundecene.

ppGpp and ppApp, which were also used for evaluation of the chemosensor (*vide infra*), were synthesized from adenosine and guanosine according to Haas *et al.*^21^ In this approach, treatment of the nucleosides with pyrophosphoryl chloride affords chlorophosphate intermediates 17 (Scheme 3), which upon hydrolysis yield the corresponding 2′,3′-cyclophosphate intermediates.^21^ When performed on a larger scale, however, the quenching step can become less selective, leading to partial, non-selective ring opening and formation of 2′/3′-regioisomeric pNp mixtures 18 and 19. These mixtures are difficult to separate by SAX or RP chromatography and thus limit straightforward access to defined 3′-monophosphate building blocks (Scheme 3, top). To address this limitation, we investigated a chemoenzymatic correction strategy (Scheme 3, bottom). The regioisomeric pNp mixtures 18 and 19 were first converted into the corresponding ppNpp derivatives 21 and 22. Subsequent incubation with RNase T2 at 37 °C and pH 5.5 led to intramolecular cyclization *via* a transient 2′,3′-cyclophosphate intermediate, followed by selective enzymatic ring opening to the 3′-position.^22^ Full conversion was confirmed by ^31^P NMR spectroscopy (see SI, Fig. S1). This sequence yields the 3′-monophosphates and ppAp (4) ppGp (5) as their ammonium salts in quantitative yields after SAX purification. While RNase T2-mediated hydrolysis of ppGpp to ppGp has been described previously,^21^ its ability to selectively process regioisomeric ppNpp mixtures – particularly ppApp – has not been reported. This newly described enzymatic reaction represents an efficient and selective method for recovering defined ppNp from regioisomeric ppNpp mixtures and underscores the practical relevance of RNase T2 for MSN synthesis. ppAp was in turn used to synthesize a clickable pppApp analogue (see SI, Chapter 3.5). Such MSN derivatives represent a versatile platform for chemical modifications and can, for example, be derivatized into pull-down probes, as previously demonstrated by Haas *et al.*^23^

### Fluorescent chemosensor for MSN

Although powerful analytical methods for MSN quantification are available,^8–12^ a simple fluorescence-based readout that operates under mild, aqueous buffer conditions remains highly attractive. Selective sensing is challenging due to the high charge density of MSNs and their close structural similarity to abundant nucleotides and other phosphorylated species. To date, only one small-molecule fluorescent chemosensor – the PyDPA sensor^24^ – has been reported. Complementary sensing strategies that are based on readily accessible and robust sensors that operate under physiological conditions are highly desirable. Here, we introduce a disassembly-based “turn-on” fluorescence probe in which coordination of MSNs to an Fe(iii)-salen complex triggers demetallation and release of a fluorescent salicylaldehyde reporter.^25,26^

**Fig. 1:**
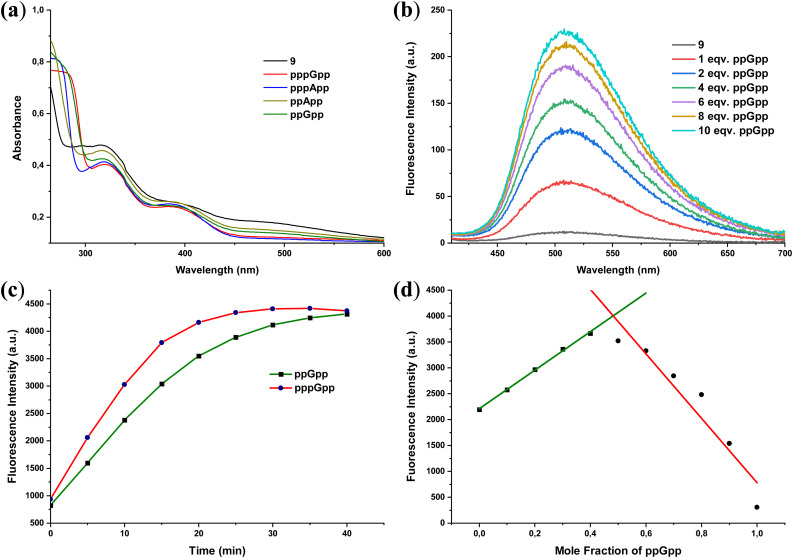
(a) UV/Vis spectra of 9 (100 μM) after incubation with 10 eq. of pppGpp, pppApp, ppGpp, or ppApp in Tris buffer (10 mM, pH 7.4) for 40 min. (b) Fluorescence titration of 9 (50 μM) with ppGpp in Tris buffer (10 mM, pH 7.4) after 40 min incubation (*λ*_ex_ = 375 nm). (c) Time-dependent fluorescence increase at *λ*_em_ = 510 nm of 9 (50 μM) in the presence of 10 eq. pppGpp or ppGpp (*λ*_ex_ = 375 nm). (d) Job's plot of compound 9 with ppGpp according to the method of continuous variation. [9] + [ppGpp] = 100 μM. *λ*_ex_ = 375 nm, *λ*_em_ = 510 nm. Fluorescence intensities are given in arbitrary units (a.u.).

Complex 9 (Fe-Sal) was prepared according to a known procedure.^25^ This Fe(iii)-salen scaffold has previously been used as a disassembly sensor for pyrophosphate. We now investigate its response toward the hyperphosphorylated alarmones (p)ppGpp and (p)ppApp and other nucleotides. Upon incubation of Fe-Sal (9) with 10 equivalents of both (p)ppGpp and (p)ppApp in Tris buffer (10 mM, pH = 7.4), a decrease in absorbance was detected at 320 nm, 392 nm, and 485 nm. The observed decrease in absorbance at these wavelengths was more pronounced when using pentaphosphate (pppGpp and pppApp) compared to tetraphosphate (ppGpp and ppApp) (Fig. 1a). Disassembly of the Fe-Sal complexes was further investigated by titration with (p)ppGpp species. Upon incremental addition of ppGpp sodium salt to a solution of Fe-Sal (50 μM) in 10 mM Tris buffer (pH 7.4), a progressive enhancement in fluorescence emission intensity was observed. Specifically, excitation at 375 nm resulted in an emission centred at 510 nm, which increased proportionally with the amount of ppGpp added. An eightfold enhancement in fluorescence intensity was recorded upon the addition of 10 equivalents of ppGpp (Fig. 1b). The disassembly process was found to be time-dependent. As the reaction progressed, a gradual increase in fluorescence intensity was observed, reaching a maximum after approximately 40 minutes for ppGpp. This behaviour indicates a dynamic disassembly process driven by interaction of the analyte with the Fe centre of the complex (Fig. 1c). pppGpp induced faster disassembly, with maximum emission reached after approximately 25 minutes. However, the overall response time precludes real-time detection under the conditions employed necessitating end-point detection.

To determine the binding stoichiometry between 9 and ppGpp, a Job's plot analysis was conducted based on emission intensity at 510 nm (Fig. 1d). The result suggested a 1 : 1 binding between 9 and ppGpp, while [9 + ppGpp] = 100 µM was maintained throughout the experiment.

**Fig. 2:**
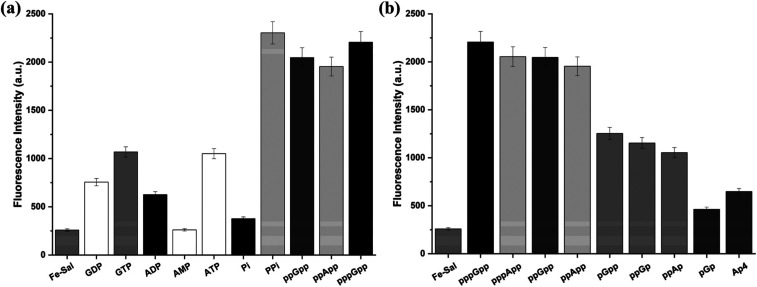
Fluorescence response of probe 9 (50 μM) toward phosphate species and alarmones measured at 510 nm 40 min after addition of 10 eq. analyte in Tris buffer (10 mM, pH 7.4). (a) Inorganic (pyro)phosphate, common nucleotides, and MSNs. (b) Structurally related alarmones. *λ*_ex_ = 375 nm; *n* = 3. Fluorescence intensities are given in arbitrary units (a.u.).

Given the pronounced response of 9 toward MSNs, we next evaluated its selectivity against a panel of biologically relevant phosphorylated molecules. To this end, the probe was incubated with inorganic phosphate (Pi), inorganic pyrophosphate (PPi) and common nucleotides (ATP, ADP, AMP, GTP, GDP, and GMP) under identical conditions (10 mM Tris buffer, pH 7.4, 40 min). As shown in Fig. 2a, the probe exhibits stronger fluorescence responses toward MSNs compared to other phosphorylated species, while no selectivity is observed against inorganic pyrophosphate (PPi).

Selectivity was further assessed using a set of structurally related bacterial alarmones, including pGpp, ppGp, ppAp, pGp, and adenosine 5′-tetraphosphate (Ap4). In this series, significantly stronger fluorescence responses were observed for pGpp, ppGp, and ppAp compared to pGp and Ap4 (Fig. 2b). This trend indicates that the presence of a pyrophosphate moiety at either the 3′ or 5′ position is a key determinant for efficient probe activation. In contrast, alarmones lacking diphosphate units at these positions elicited weaker responses, underscoring the importance of both phosphorylation state and phosphate positioning for recognition by 9.

**Fig. 3:**
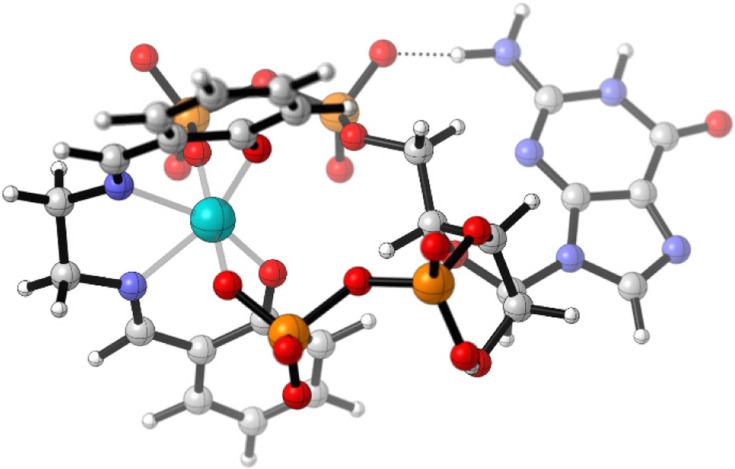
DFT-optimized structure of the mono-ppGpp complex [Fe(salene)(ppGpp)]^5−^ (high-spin configuration). Relative Gibbs free energies of all calculated Fe-Sal-ppGpp species are provided in the SI.

We further investigated the interaction of ppGpp with probe 9 by DFT calculations. Starting from the water-coordinated complex [Fe(salen)(H_2_O)_2_], ligand substitution by ppGpp was examined. Coordination of ppGpp to the Fe(iii) centre with displacement of both water ligands affords a thermodynamically favoured mono-ppGpp complex, [Fe(salen)(ppGpp)]^5−^. The optimized high-spin structure (Fig. 3) reveals bidentate coordination of the polyphosphate chain to iron.

Overall, the calculated Gibbs free energies support a strong thermodynamic driving force for ligand exchange of the Fe-Sal complex with ppGpp, consistent with the experimentally observed fluorescence activation. A complete overview of calculated species and relative Δ*G* values is provided in the SI.

To further support the proposed disassembly mechanism, ^1^H NMR experiments were performed using a water-soluble sulfonated analogue of the Fe-Sal complex (Fe-Sal-SO_3_), which facilitated measurements at higher concentrations required for NMR. Upon incubation with ppGpp, a new resonance at *ca.* 10 ppm appeared, consistent with the aldehyde proton of the liberated salicylaldehyde ligand. This observation provides additional evidence for ppGpp-induced complex disassembly *via* competitive coordination to the Fe(iii)-center (for full spectra and signal assignment see SI, Chapter 6).

**Scheme 4:**
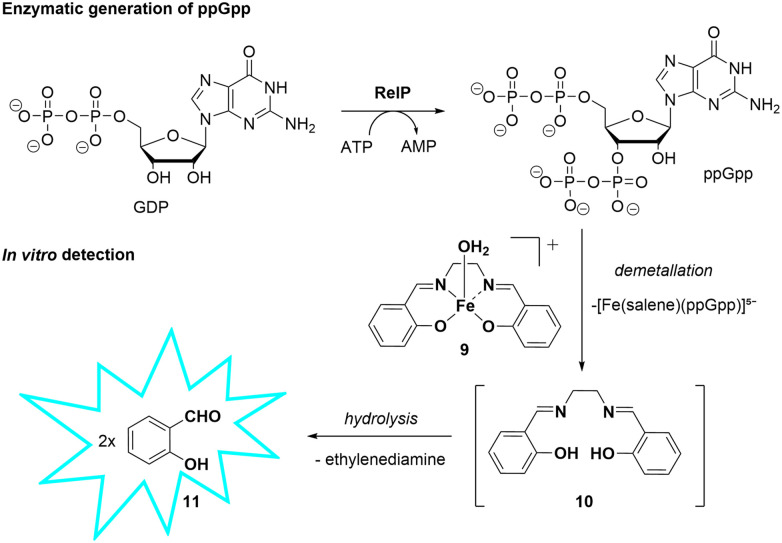
Enzymatic generation of ppGpp by RelP and its subsequent detection *via* Fe-Salen probe disassembly.

To evaluate the applicability of probe 9 for the detection of enzymatically generated ppGpp, we conducted an *in vitro* enzymatic assay using the *S. aureus* RelP synthetase (Scheme 4).^27^

**Fig. 4:**
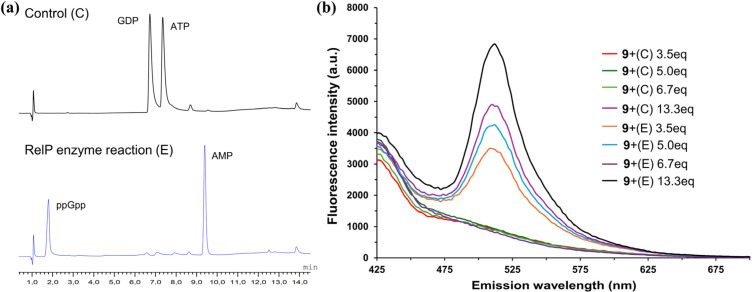
(a) HPLC chromatogram of enzymatic reaction of RelP. (b) Fluorescence emission spectra of probe 9 (50 µM) after addition of aliquots from the RelP reaction mixture (E) corresponding to 3.5–13.3 equiv. ppGpp relative to probe concentration. Control reactions without RelP (C) are shown for comparison. Conditions: *λ*_ex_ = 375 nm, Tris buffer (10 mM, pH 7.4). Fluorescence intensities are given in arbitrary units (a.u.).

The reaction mixture contained ATP (2 mM), GDP (2 mM), and RelP (10 µM) in Tris-HCl buffer (20 mM, pH 8.0), MgCl_2_ (15 mM), KCl (15 mM), and β-mercaptoethanol (1.5 mM). A 1 : 1 ATP : GDP ratio was used to reduce interference from excess ATP given the limited selectivity of probe 9. The reaction was incubated at 37 °C for 1 hour and HPLC analysis confirmed complete consumption of the ATP and GDP substrates, indicating successful and selective synthesis of ppGpp (Fig. 4a). The reaction was subsequently quenched by heat denaturation, and aliquots of the reaction mixture were added to a solution of probe 9 (50 µM in Tris buffer, pH 7.4) to afford final ppGpp equivalents of 3.5, 5.0, 6.7, and 13.3 relative to the probe concentration. Following incubation for 45 minutes, fluorescence emission spectra revealed a significant increase in emission intensity, consistent with the disassembly of the Fe-based probe 9 (Fig. 4b). This fluorescence enhancement is attributed to the coordination of enzymatically produced ppGpp to the iron centre, leading to displacement of the ligand and release of the fluorescent aldehyde. This probe has potential for applications in inhibitor screening of ppGpp synthetases, provided that the nucleotide ratios described herein (1 : 1 ATP : GDP) are maintained to minimize ATP/GDP interference and pyrophosphate is absent. Notably, RSH enzymes comprise both mono- and bifunctional members, with RelA and small alarmone synthetases lacking hydrolase activity, whereas bifunctional enzymes such as SpoT can generate PPi;^2,28^ in such cases, PPi levels may need to be controlled, for example by enzymatic removal (*e.g.*, pyrophosphatase). Accordingly, the applicability of the probe is limited to defined *in vitro* systems as described herein.

## Conclusions

In summary, a combined chemical and analytical strategy was developed for the investigation of bacterial alarmone nucleotides. A scalable chemo-enzymatic synthesis enables efficient access to the natural MSNs pppApp and pppGpp on a gram scale *via* cPyPa-mediated phosphorylation, ethylenediamine-induced ring opening, phosphate excision followed by RNase T2-catalysed hydrolysis. In addition, the chemoenzymatic isomerisation of 2′/3′-isomeric ppNpp intermediates by RNase T2 allows the production of defined ppNp building blocks. With access to a diverse set of MSN and other phosphorylated metabolites, a fluorescence probe based on Fe-salen disassembly was developed for the detection of (p)ppGpp and (p)ppApp. The coordination of the MSN to an Fe(iii)-salen complex leads to demetallation and fluorescence dequenching through the release of salicylaldehyde. Different methods (Job's plot, NMR, DFT) support ligand exchange and disassembly as the sensing mechanism in a 1 : 1 stoichiometry. The probe exhibits moderate selectivity for (p)ppGpp and (p)ppApp over common nucleosides (*ca.* twofold for GDP and ADP and threefold for GTP and ATP), indicating that the presence of a di- or triphosphate ester unit alone is insufficient for complete activation within the studied time frame of 40 minutes. Notably, 5′-triphosphorylated alarmones (pppGpp & pppApp) induce a stronger and more rapid response than their diphosphate counterparts (ppGpp & ppApp). The system also detects enzymatically formed ppGpp from *S. aureus* RelP under optimised conditions. Together, the synthesis and sensing strategies provide a useful set of tools for accessing, modifying and functionally analysing MSN. This creates new opportunities for chemical biology applications, such as screenings for inhibitors of MSN synthesizing enzymes.

## Author contributions

P. M. synthesized the nucleotides together with M. H., P. M. and S. K. R. wrote the first draft of the manuscript. S. K. R. synthesized the sensor, evaluated its photophysical properties and performed the RelP assay together with F. W., F. B. performed the DFT calculations. B. B. supervised the theoretical studies. H. J. J. conceived the project and provided feedback on the first draft of the manuscript. All authors contributed to finalizing the manuscript.

## Conflicts of interest

There are no conflicts to declare.

## Supplementary Material

OB-024-D6OB00328A-s001

## Data Availability

Data are available in the supplementary information (SI). Supplementary information: experimental procedures, spectroscopic data (NMR, HPLC, HRMS), photophysical measurements, and DFT studies. See DOI: https://doi.org/10.1039/d6ob00328a.
