# Supplementary material for: Pentaphosphorylated magic spot nucleotides: chemoenzymatic synthesis and disassembly-based sensing
Source: Org Biomol Chem. 2026 Apr 14;24(18):3918–25. doi: 10.1039/d6ob00328a (PMC13103940; doi:10.1039/d6ob00328a)
Supplement: OB-024-D6OB00328A-s001 [file OB-024-D6OB00328A-s001.pdf]

Supplementary Information (SI) for Organic & Biomolecular Chemistry.
This journal is © The Royal Society of Chemistry 2026

Pentaphosphorylated Magic Spot Nucleotides: Chemoenzymatic Synthesis and Disassembly-Based Sensing

Patrick Moser, Subhra Kanti Roy, Marvin Herzog, Felix Bauer, Felix Wollensack, Bernhard Breit, and Henning J. Jessen

Supporting Information

Contents

1. Abbreviations 2
2. Synthetic methods and analysis 4
3. Experimental procedures 5
3.1. Synthesis of Fmoc-G, ppNpp and P-amedites 5
3.2. Synthesis of ppAp and ppGp 6
3.3. Synthesis of pppApp and pppGpp 7
3.4. Synthesis of ppNp from regioisomeric ppNpp mixtures 9
3.5. Synthesis of clickable pppApp 10
3.6. Synthesis of Fe-Sal complex 13
4. Spectroscopic studies 14
4.1. Materials and Methods 14
4.2. Time-Dependent Fluorescence Response 15
4.3. Fluorescence Titration and Concentration Dependence 16
4.4. Selectivity toward Phosphate Species 17
4.5. Fluorescence intensity of Salen complex with different phosphate compounds 18
5. DFT studies 19
6. NMR spectra 35
7. HRMS analysis 67
8. References 69

1. Abbreviations

Äkta®: chromatography system (GE Healthcare)
Aq.: aqueous
br s: broad singlet
calcd: calculated
*c*PyPA: cyclic pyrophosphoryl phosphoramidite
CuAAC: copper-catalyzed azide–alkyne cycloaddition
D_2O: deuterium oxide
DBU: 1,8-diazabicyclo[5.4.0]undec-7-ene
DIPEA: N,N-diisopropylethylamine
DMF: N,N-dimethylformamide
DMSO: dimethyl sulfoxide
d: doublet
dd: doublet of doublets
ddd: doublet of doublet of doublets
eq: equivalents
ESI: electrospray ionization
Et_2O: diethyl ether
ETT: 5-(ethylthio)-1H-tetrazole
Fmoc: 9-fluorenylmethoxycarbonyl
g: gram
h: hour(s)
HCl: hydrochloric acid
HEPES: 4-(2-hydroxyethyl)-1-piperazineethanesulfonic acid
HPLC: high-performance liquid chromatography
HRMS: high-resolution mass spectrometry
Hz: Hertz
IP_6 = myo-Inositol hexakisphosphate
IP_7 = myo-Inositol heptakisphosphate
IEFPCM: integral equation formalism polarizable continuum model
J: coupling constant
ku: kilo unit(s) (enzyme activity)
LC-MS: liquid chromatography–mass spectrometry
m: multiplet
mCPBA: meta-chloroperbenzoic acid
MeCN: acetonitrile
MeOH: methanol
MHz: Megahertz
MPLC: medium-pressure liquid chromatography
MS: mass spectrometry
n: number of replicates
NMR: nuclear magnetic resonance
ON: overnight

PBS: phosphate-buffered saline
PPC (or IEFPCM=water): implicit solvent model (water)
ppApp: adenosine-3′,5′-bis(diphosphate)
ppAp: adenosine-3′-phosphate-5′-diphosphate
ppGpp: guanosine-3′,5′-bis(diphosphate)
ppGp: guanosine-3′-phosphate-5′-diphosphate
pppApp: adenosine-3′-diphosphate-5′-triphosphate
pppGpp: guanosine-3′-diphosphate-5′-triphosphate
q: quartet
Q-Sepharose: strong anion-exchange resin
quant.: quantitative
RP-MPLC: reversed-phase medium-pressure liquid chromatography
rt: room temperature
s: singlet
SD: standard deviation
scf=xqc: quadratic convergence option in self-consistent field calculations
SDD: Stuttgart/Dresden effective core potential
SI: Supporting Information
t: triplet
TBA: tetrabutylammonium
TBA-Br: tetrabutylammonium bromide
TEAA: triethylammonium acetate
THPTA: tris(3-hydroxypropyltriazolylmethyl)amine
Tris: tris(hydroxymethyl)aminomethane
UB3LYP: unrestricted B3LYP functional
UV/Vis: ultraviolet/visible
v/v: volume/volume
vac.: vacuum
ζ (zvp): zero-point vibrational energy
λ_ex: excitation wavelength

2. Synthetic methods & analyses

General remarks

Reactions were carried out using flame-dried glassware under an atmosphere of dry N_2 and magnetically stirred, unless noted otherwise. Air- and moisture-sensitive liquids and solutions were transferred via syringe or stainless steel cannula.

Reagents were purchased from commercial suppliers (Acros, Aldrich, Fluka, TCI) and used without further purification, unless noted otherwise. DBU (1-8-diazabicyclo[5.4.0]undec-7-en) was distilled under high vacuum and stored over molecular sieves before usage.

Solvents were obtained in analytical grade and used as received for extractions, precipitation and solid washing. Dry DMF, DMSO and MeCN for reactions were purchased in a dry form from Sigma and stored over molecular sieves as well as under an atmosphere of dry N_2. Dry Et_2O for reactions was purified by filtration and dried by passage over activated anhydrous neutral A-2 alumina (MBraun solvent purification system) under an atmosphere of dry N_2.

Ribonuclease T2 from Aspergillus oryzae (50 ku) was purchased from Worthington Biochemical Corporation as lyophilized powder and dissolved in a storage buffer [glycerol / NaH_2PO_4 (10 mM, pH 6.8), 1/1]. The stock solution was stored at -20 °C.

Deuterated solvents for NMR and reactions were obtained from Armar Chemicals, Switzerland and euriso-top, Germany, in the indicated purity grade and used as received for NMR spectroscopy.

Strong ion-exchange chromatography was performed using an automated Äkta®-system. Q-Sepharose was purchased from Aldrich. Buffer solutions were produced manually using milliQ H_2O.

Preparative RP-MPLC was performed using an automated Interchim®-system. The AQ- solid phase was purchased from Interchim. Lyophilizations were done with Christ Freeze Dryer Alpha 1-4 LDplus and Christ Freeze Dryer Alpha 1-2 LDplus.

^{1}H-NMR spectra were recorded on Bruker 300 MHz spectrometers, Bruker 400 MHz (with cryoprobe) and Bruker 500 MHz spectrometers in the indicated deuterated solvent. Data are reported as follows: chemical shift (δ, ppm), multiplicity (s, singlet; d, doublet; t, triplet; q, quartet; m, multiplet; br. s, broad signal), coupling constant(s) (*J*, Hz), integration. All signals were referenced to the internal solvent signal as standard (D_2O, δ 4.70; DMF-d7, δ 2.94).

^{13}C{^{1}H}-NMR spectra were recorded with ^{1}H-decoupling on Bruker 126 MHz, Bruker 101 MHz (with cryoprobe) spectrometers at 298 K in the indicated deuterated solvent.

^{31}P{^{1}H}-NMR spectra and **^{31}P-NMR** spectra were recorded with ^{1}H-decoupling or 1H coupling, respectively, on Bruker 202 MHz , 162 MHz (with cryoprobe) and Bruker 122 MHz spectrometers in the indicated deuterated solvent. All signals were referenced to an internal standard (PPP).

Mass spectra were recorded by C. Warth (Mass spectrometry service of the University of Freiburg) on a Thermo LCQ Advantage [spray voltage: 2.5 - 4.0 kV, spray current: 5 µA, ion transfer tube: 250 (150) °C, evaporation temperature: 50 - 400 °C.

3. Experimental Procedures

3.1. Synthesis of Fmoc-G, ppApp, ppGpp & P-amidites

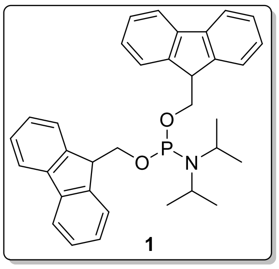

(FmO)$_2$P-N(*i*Pr)$_2$ **1** was synthesized in two steps starting from PCl_3 as reported previously. Analytical data were identical to literature.[1] The compound was stored at −20 °C.

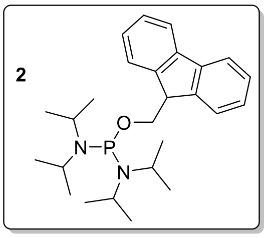

(FmO)P-N[(*i*Pr)$_2$]$_2$ **2** was synthesized as reported previously. Analytical data were identical to literature.[2] The compound was stored at −20 °C.

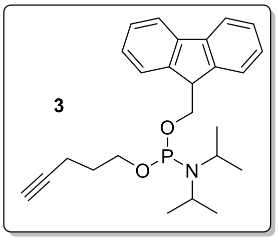

(PentynylO)(FmO)P-N(*i*Pr) **3** was synthesized as reported previously. Analytical data were identical to literature.[3] The compound was stored at −20 °C.

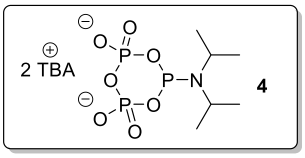

Cyclic pyrophosphoryl-*P*-amidite (cPyPA,**4**) was synthesized according to literature[4] and stored over molecular sieves at −20 °C.

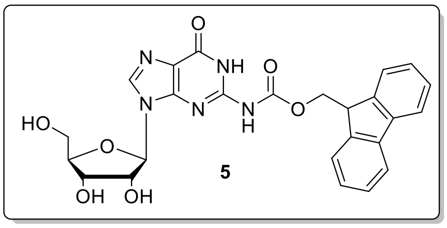

2-*N*-(9-Fluorenylmethoxycarbonyl)guanosine (**5**) was synthesized in one step from guanosine according to Quaedflieg et al. Analytical data were identical to literature.[5]

3.2. Synthesis of nucleotide 3'-phosphate 5'-diphosphates ppAp & ppGp

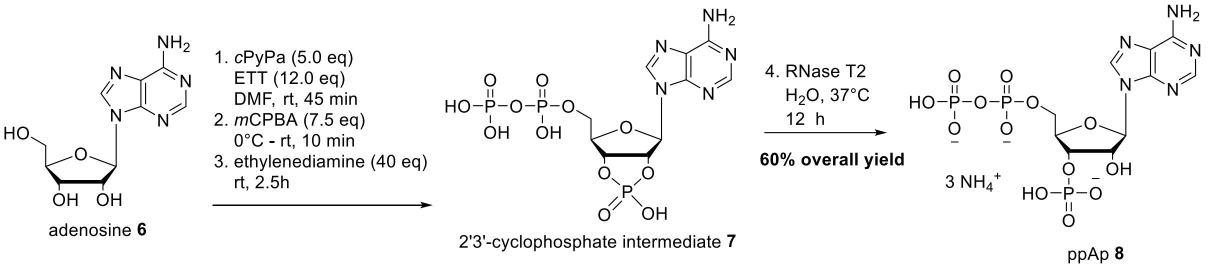

Adenosine (**6**, 500 mg, 2.00 mmol) and ETT (2.92 g, 24.0 mmol, 12.0 eq) were coevaporated together with dry MeCN (2 × 4.0 mL) in a flame dried roundbottom flask. The residue was dissolved in dry DMF (30.0 mL). Then *c*PyPA (0.4 M in DMF, 25.0 mL, 9.0 mL, 5.0 eq) was added dropwise within 5 min, and the resulting solution was stirred for 45 min at rt. The solution was cooled to 0 °C, and *m*CPBA (77%, 3.15 g, 7.0 mmol, 7.5 eq) was added within 5 min in portions. The mixture was stirred at 0 °C for 10 min. To the reaction mixture was added ethylenediamine (5.0 mL, 37.5 mmol, 40.0 eq) and the resulting solution was stirred for 2.5 h at rt, before being precipitated by the addition of Et_2O (200 mL). The resulting solid was dissolved in H_2O (50 mL), and RNase T2 (500 u) was added. The solution was incubated at 37 °C for 12h. Afterwards the solution was directly applied to a strong ion-exchange column (Q-Sepharose). The product was eluted using NH_4HCO_3 – buffer (350 mM). Lyophilization of the product containing fractions (analyzed via ^{31}P NMR) afforded the desired product (**8**, 791 mg, 1.28 mmol, 64 %) as white solid.

^{1}H NMR (400 MHz, D_2O, δ/ppm) 8.55 (d, *J* = 1.4 Hz, 1H), 8.30 – 8.24 (m, 1H), 6.24 – 6.16 (m, 1H), 4.91 – 4.85 (m, 2H), 4.63 (t, *J* = 2.6 Hz, 1H), 4.28 (dd, *J* = 5.4, 2.8 Hz, 2H).

^{13}C NMR (101 MHz, D_2O, δ/ppm) δ 154.93, 152.01, 149.08, 140.06, 118.52, 86.52, 83.35 (dd, *J* = 9.1, 3.7 Hz), 73.75 (d, *J* = 4.8 Hz), 65.02 (d, *J* = 5.4 Hz), 36.71.

^{31}P NMR (162 MHz, D_2O, δ/ppm) δ 4.09, -5.91 (d, *J* = 16.4 Hz), -10.50 (d, *J* = 16.4 Hz).

HRMS (ESI): m/z calcd for $C_{10}H_{15}N_5O_{13}P_3$ $[M-H]^-$ 505.9884; found: 505.9884.

The compounds were previously characterized, and the analytical data matched those reported in the literature. [6]

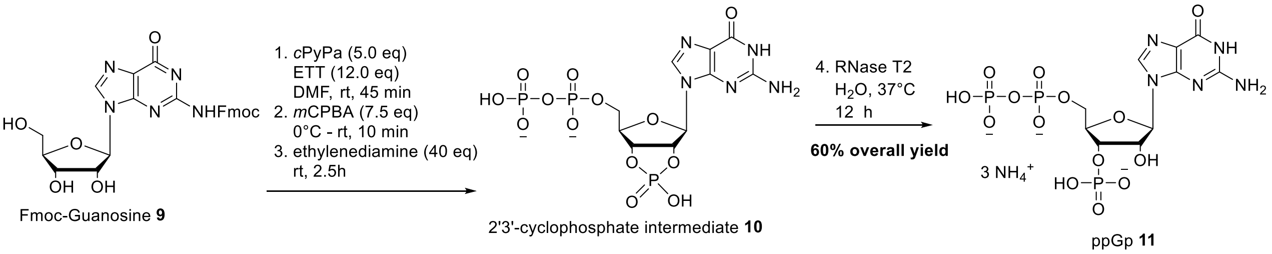

Fmoc-guanosine (**9**, 500 mg, 1.00 mmol) and ETT (1.46 g, 12.0 mmol, 12.0 eq) were coevaporated together with dry MeCN (2 × 4.0 mL) in a flame dried roundbottom flask. The residue was dissolved in dry DMF (15.0 mL). Then *c*PyPA (0.4 M in DMF, 12.5 mL, 4.5 mL, 5.0 eq) was added dropwise within 5 min, and the resulting solution was stirred for 45 min at rt. The solution was cooled to 0 °C, and *m*CPBA (77%, 1.57 g, 3.5 mmol, 7.5 eq) was added within 5 min in portions. The mixture was stirred at 0 °C for 10 min. To the reaction mixture was added diethylamine (2.5 mL, 18.8 mmol, 40.0 eq) and the resulting solution was stirred for 2.5 h at rt, before being precipitated by the addition of Et_2O (100 mL). The resulting solid was dissolved in H_2O (35 mL), and RNase T2 (300 u) was added. The solution was incubated at 37 °C for 12h. Afterwards the solution was directly applied to a strong ion-exchange column (Q-Sepharose). The product was eluted using NH_4HCO_3 – buffer (350 mM). Lyophilization of the product containing fractions (analyzed via ^{31}P NMR) afforded the desired product (**11**, 201 mg, 0.42 mmol, 42 %) as white solid.

^{1}H-NMR (400 MHz, D_2O, δ/ppm): 8.17 (s, 1H), 5.99 (d, *J* = 6.7 Hz, 1H), 4.89 – 4.74 (m, 2H), 4.64 – 4.53 (m, 1H), 4.32 – 4.21 (m, 2H).

^{13}C{^{1}H}-NMR (101 MHz, D_2O, δ/ppm): 153.1, 154.0, 152.0, 137.8, 116.3, 86.5, 83.6 (dd, *J* = 9.4, 4.2 Hz), 73.7 (d, *J* = 4.9 Hz), 73.3 (d, *J* = 4.4 Hz), 65.2 (d, *J* = 5.3 Hz).

^{31}P{^{1}H}-NMR (162 MHz, D_2O, δ/ppm): 1.95, -9.63 (d, *J* = 21.0 Hz), -11.06 (d, *J* = 20.8 Hz).

HRMS (ESI) m/z for $C_{10}H_{15}N_5O_{14}P_3$ $[M-H]^-$: calcd 521.9829, found 521.9832.

The compounds were previously characterized, and the analytical data matched those reported in the literature. [3]

3.3. Synthesis of pppGpp and pppApp

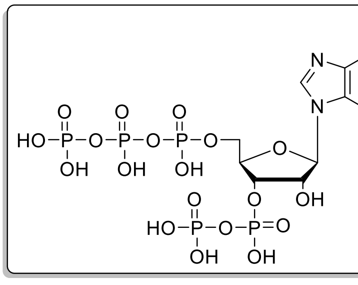

12

To a solution of ppGp x 3.0 TBA (747 mg, 600 μmol) in dry DMF (10 ml) was added ETT (468 mg, 3.60 mmol, 6.0 eq.). Afterwards, a solution of **1** (1.17 g, 1.80 mmol, 3.0 eq.) in DMF (6.5 ml) was added in one portion and the resulting mixture was stirred for 15 min at room temperature. Afterwards, the solution was cooled to −20 °C and *m*CPBA (77%, 488 mg, 1.98 mmol, 3.3 eq.) was added. The solution was stirred for 5 min at −20 °C and for 10 min at 0 °C.

Afterwards, distilled DBU (1.6 mL) was added at 0 °C. The solution was stirred for 5 min at 0 °C and 25 min at room temperature before precipitation was induced by addition of Et_2O (100 mL). The precipitate was separated by centrifugation, washed with Et_2O (3 x 10 mL) and dried under high vac. The crude product was purified by strong ion-exchange chromatography (Q-Sepharose) using an Äkta-system and $NaClO_4$ – buffer (100 mM). The product containing fractions (analyzed via ^{31}P NMR) were precipitated using an 8-fold volume of $NaClO_4$ – solution (−20 °C, 500 mM in acetone). The resulting solid was washed with acetone (−20 °C, 3 x 10 mL) and dried under high vac for 2 h. The product (**12**) (429 mg, 516 µmol, 89%) was isolated as a white solid.

^{1}H-NMR (400 MHz, D_2O, δ/ppm): 8.08 (s, 1H), 5.94 (d, *J* = 6.5 Hz, 1H), 4.89 (ddd, *J* = 8.4, 5.0, 2.9 Hz, 1H), 4.92 – 4.86 (m, 1H), 4.51 – 4.44 (m, 1H), 4.24 – 4.14 (m, 2H).

^{13}C{^{1}H}-NMR (101 MHz, D_2O, δ/ppm): 159.2, 154.0, 152.0, 138.0, 116.3, 86.9, 83.3 (dd, *J* = 9.1, 4.3 Hz), 74.9 (d, J = 5.1 Hz), 73.1 (d, J = 4.2 Hz), 65.5 (d, J = 5.2 Hz).

^{31}P{^{1}H}-NMR (162 MHz, D_2O, δ/ppm): -5.60 (d, *J* = 21.9 Hz), -10.70 (d, *J* = 16.0 Hz), -10.85 (d, *J* = 13.9 Hz), -10.97 (d, *J* = 10.0 Hz), -21.09 (dd, *J* = 18.9 Hz).

HRMS (ESI) m/z for $C_{10}H_{16}N_5O_{20}P_5$ $[M-H]^-$: calcd 681.90088, found 681.9161.

The compounds were previously characterized, and the analytical data matched those reported in the literature.[6]

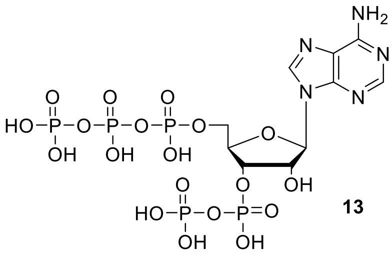

13

To a solution of ppAp x 3.0 TBA (1.37 g, 0.11 mmol) in dry DMF (20 ml) was added ETT (859 mg, 0.66 µmol, 6.0 eq.). Afterwards, a solution of **1** (2.15 mg, 3.30 mmol, 3.0 eq.) in DMF (10 ml) was added in one portion and the resulting mixture was stirred for 15 min at room temperature. Afterwards, the solution was cooled to −20 °C and *m*CPBA (77%, 895 mg, 3.63 mmol, 3.3 eq.) was added. The solution was stirred for 5 min at −20 °C and for 10 min at 0 °C. Afterwards, distilled DBU (3 mL) was added at 0 °C. The solution was stirred for 5 min at 0 °C and 25 min at room temperature before precipitation was induced by addition of Et_2O (300 ml). The precipitate was separated by centrifugation, washed with Et_2O (3 x 10 ml) and dried under high vac. The crude product was purified by strong ion-exchange chromatography (Q-Sepharose) using an Äkta-system and $NaClO_4$ – buffer (100 mM). The product containing fractions (analyzed via ^{31}P NMR) were precipitated using an 8-fold volume of $NaClO_4$ – solution (−20 °C, 500 mM in acetone). The resulting solid was washed with acetone (−20 °C, 3 x 10 ml) and dried under high vac for 2 h. The product (**13**) (59.3 mg, 70.8 µmol, 64%) was isolated as a white solid.

^{1}H-NMR (400 MHz, D_2O, δ/ppm): 8.53 (s, 1H), 8.20 (s, 1H), 6.11 (d, *J* = 6.5 Hz, 1H), 4.76 (ddd, *J* = 8.4, 5.0, 2.9 Hz, 1H), 4.82 – 4.76 (m, 1H), 4.41 – 4.34 (m, 1H), 4.14 – 4.04 (m, 2H).

^{13}C{^{1}H}-NMR (101 MHz, D_2O, δ/ppm): 155.7, 152.9, 149.5, 140.1, 118.7, 86.4, 84.4 (dd, *J* = 9.1, 4.3 Hz), 73.7 (d, *J* = 5.1 Hz), 73.7 (d, *J* = 4.2 Hz), 65.3 (d, *J* = 5.2 Hz).

^{31}P{^{1}H}-NMR (162 MHz, D_2O, δ/ppm): -6.53 (d, *J* = 21.9 Hz), -7.91 (d, *J* = 16.0 Hz), -10.85 (d, *J* = 13.9 Hz), -10.97 (d, *J* = 10.0 Hz), -21.77 (dd, *J* = 18.9 Hz).

HRMS (ESI) m/z for $C_{10}H_{16}N_5O_{20}P_5$ $[M-H]^-$: calcd 607.935644, found 607.9368.

The compounds were previously characterized, and the analytical data matched those reported in the literature.[6]

3.4. Synthesis of ppAp & ppGp from regioisomeric ppApp & ppGpp mixtures

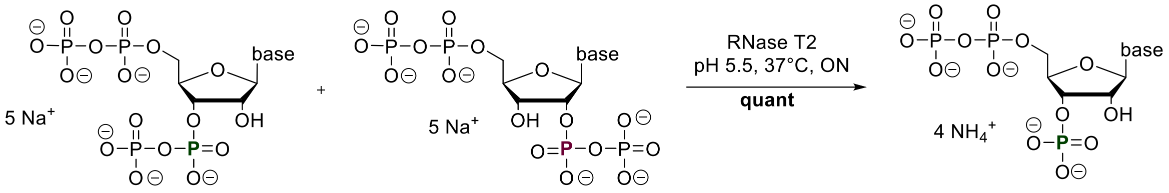

*5',3'- and 5',2'- regioisomeric ppApp (**14**) or ppGpp (**15**) mixture*

*regioisomerically pure 5',3' ppAp (**8**) & ppGp (**11**)*

The 5′,3′- and 5′,2′-regioisomeric ppNpp mixture (2.00 mmol, 1.36 g for **14**, 1.39 g for **15**) was dissolved in H_2O (50 mL) and acidified to pH = 5.5 using 1 M HCl. Subsequently, ribonuclease T2 (500 U) was added and the solution was incubated at 37 °C for 12 h. The progress of the reaction was monitored by ^{31}P NMR spectroscopy, indicating complete conversion to a single regioisomer (Fig S1 & S2). The reaction mixture was then applied to a strong anion-exchange column (Q-Sepharose). ppNp was eluted using NH_4HCO_3 buffer (350 mM). Lyophilization of the product-containing fractions (analyzed via ^{31}P NMR) afforded the desired product (2.00 mmol, quantitative yield, 1.16 g for **8**, 1.39 g for **11**) as a white solid. The analytical data are consistent with those of ppGp and ppAp obtained via the alternative synthetic routes described above.

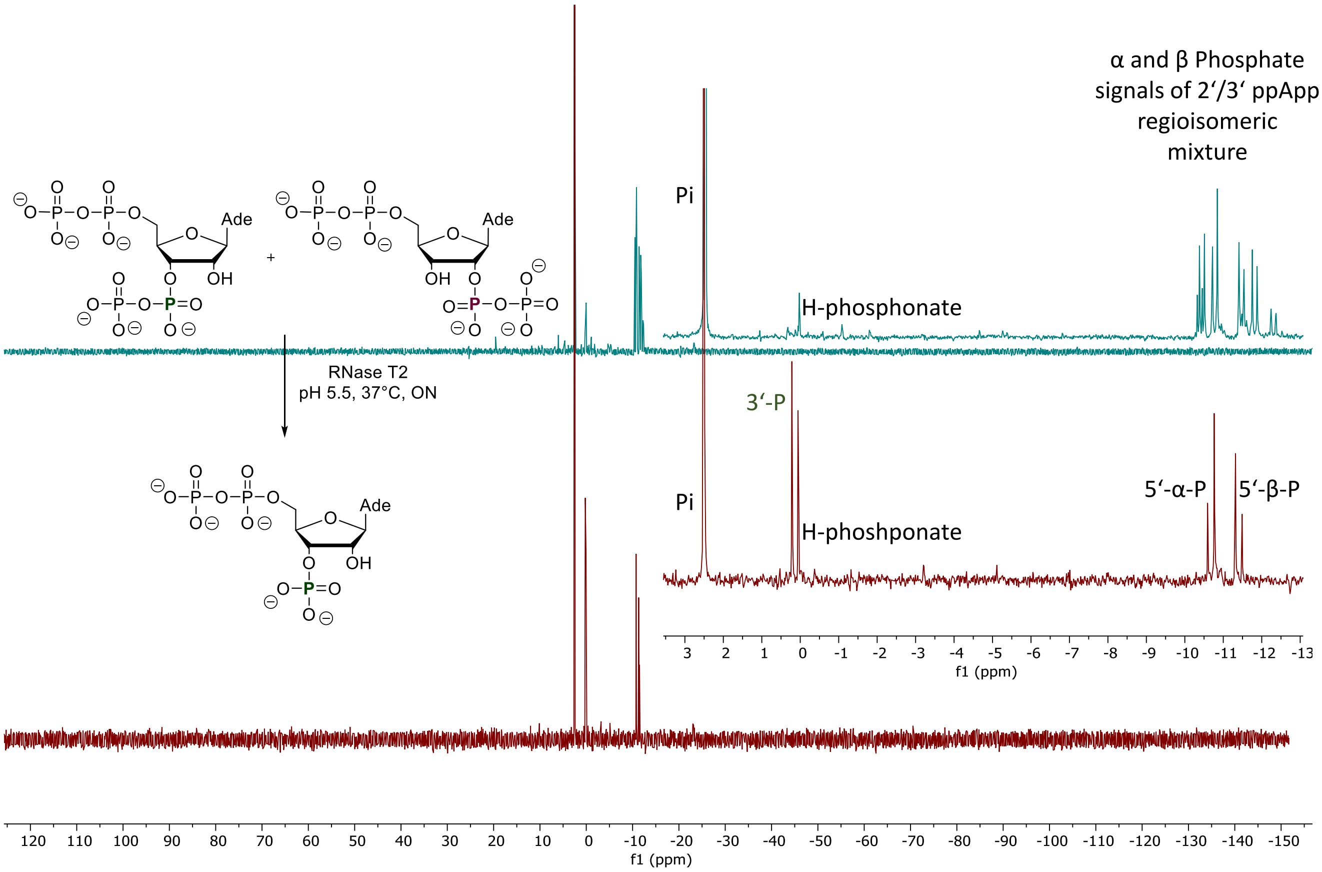

Figure S1. ^{31}P NMR monitoring of the RNase T2–mediated conversion of a regioisomeric ppGpp mixture to ppGp (pH 5.5, 37 °C, 12 h).

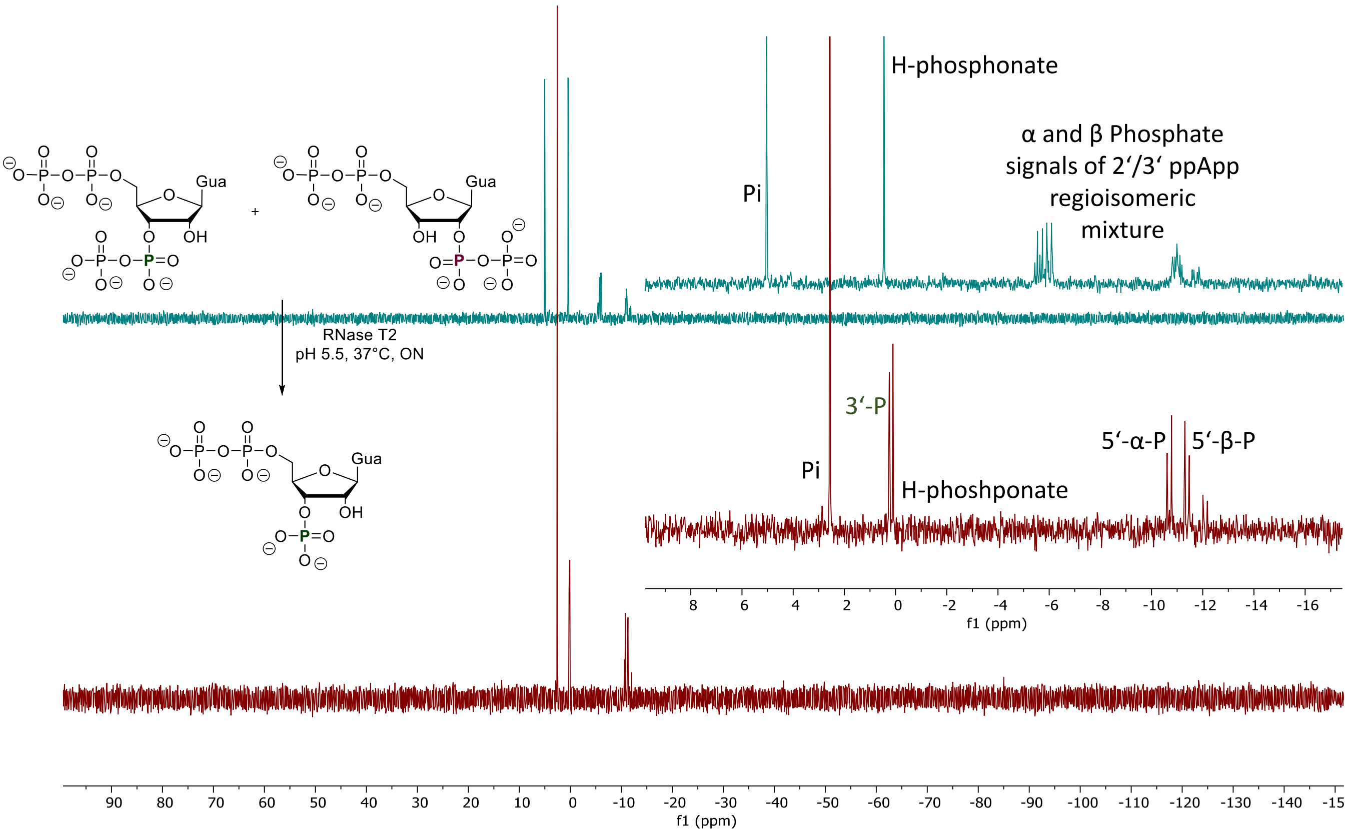

Figure S2. ^{31}P NMR monitoring of the RNase T2–mediated conversion of a regioisomeric ppApp mixture to ppAp (pH 5.5, 37 °C, 12 h

3.5. Synthesis of clickable pppApp

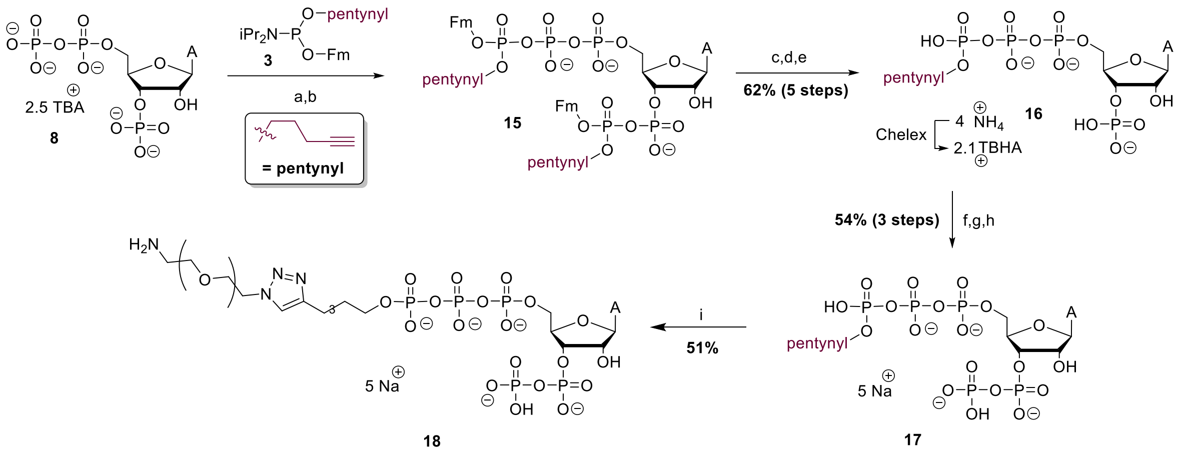

Scheme S1 Synthesis of 5'Amino-pppApp (**18**): (a) **3** (2.2 eq.), ETT (5.0 eq.), DMF. rt, 15 min. (b) *m*CPBA (2.2 eq.), 0°C, 15 min. (c) MeOH, sonication for 5 min. (d) Piperidine, DMF, rt, 30 min. (e) RNase T2, H_2O, 12 h. (f) **1** (1.3 eq.), ETT (3.0 eq.), rt, 15 min. (g) *m*CPBA (1.3 eq.), 0°C, 15 min. (h) DBU, 0°C – rt, 30 min. (i) 1-amino-11-azido-3,6,9-trioxa-undecane (1.5 eq.), sodium ascorbate (10 eq.), $CuSO_4$ (0.1 eq.) THPTA (0.5 eq.), 1x PBS – buffer, rt, 3 h.

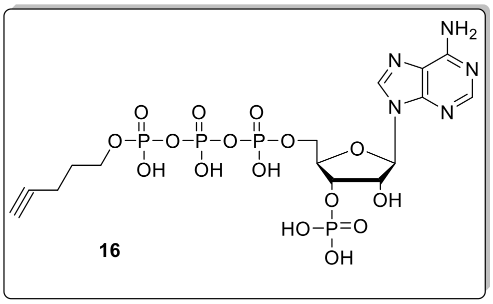

ppAp (**8**) × 2.0 TBA (294 mg, 297 µmol, 1.0 eq.) was dissolved in DMF (8.5 ml), and ETT (135 mg, 1.04 mmol, 3.5 eq.) was added. Afterwards, a solution of phosphoramidite **1** (304 mg, 742 µmol, 2.5 eq.) in DMF (5.5 ml) was added, and the resulting solution was stirred for 15 min at room temperature. The solution was cooled to −5 °C, and *m*CPBA (77%, 166 mg, 742 µmol, 2.5 eq.) was added. After stirring for 10 min at −5 °C, precipitation was induced by adding Et_2O (40 ml). The resulting solid was washed with ether (2 × 15 ml) and dried under high vacuum before being dissolved in MeOH (14 ml). The resulting solution was stirred for 1 h at room temperature. The solvent was removed under reduced pressure, and the residue was dissolved in DMF (14 ml). Piperidine (708 µl) was added at room temperature. After stirring at room temperature for 30 min, precipitation was induced by the addition of Et_2O (40 ml). The resulting precipitate was washed with Et2O (2 × 20 ml) and dried under high vacuum. The solid was redissolved in H_2O (20 ml) and acidified with HCl to pH = 5.3. Afterwards, *RNase T2* (150 µl) was added, and the solution was incubated at 37 °C overnight. The crude product was purified by strong ion-exchange chromatography (Q-Sepharose) using an Äkta system and a $NaClO_4$ buffer (110-130 mM). The product-containing fractions (analyzed via ^{31}P NMR) were precipitated using an 8- fold volume of NaClO4 solution (−20 °C, 500 mM in acetone). The resulting solid was washed with acetone (−20 °C, 3 × 10 ml) and dried under high vacuum for 2 h. The product **16** (62.0 mg, 81.3 µmol, 27%) was isolated as a white solid. It was converted

into its TBA salt by cation exchange on a Chelex column, loaded with TBA-Br followed by lyophilization.

^{1}H-NMR (400 MHz, D_2O) δ = 8.60 (s, 1H), 8.29 (s, 1H), 6.20 (d, *J* = 7.2 Hz, 1H), 4.84 (mc, 1H), 4.78-4.75 (m, 1H), 4.27 (mc, 2H), 4.60 (ddd, *J* = 2.9, 2.9, 2.8 Hz, 1H), AB-Signal (δA = 3.96, δB = 3.94, *J*AB = 6.5 Hz, 2H), 2.25 – 2.22 (m, 1H), 2.17 (ddd, *J* = 7.5, 6.9, 2.5 Hz, 2H), 1.76-1.61 (m, 2H) ppm.

^{31}P{^{1}H}-NMR (162 MHz, D_2O) δ = 4.07 (s), −10.79 (d, *J* = 18.7 Hz), −11.20 (d, *J* = 17.9 Hz), −22.70 (dd, *J* = 18.3, 18.3 Hz) ppm.

^{13}C{^{1}H}-NMR (101 MHz, D_2O) δ = 155.7, 153.0, 149.5, 140.0, 118.7, 86.4, 84.9, 84.1 (dd, *J* = 9.3, 4.2 Hz), 74.9 (d, *J* = 5.2 Hz), 73.7, 69.1, 65.5 (d, *J* = 5.4 Hz), 65.2 (d, *J* = 5.9 Hz), 28.7 (d, *J* = 7.2 Hz), 14.1 (d, *J* = 2.9 Hz) ppm.

HRMS (ESI-): *m/z* for $C_{15}H_{21}N_5NaO_{16}P_4^-$ [M-H]$^-$ calcd. 673.9836, found 673.9836.

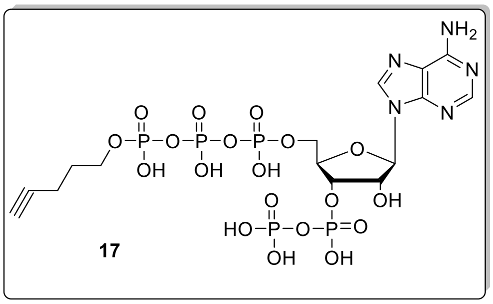

Pentynyl-pppAp (**16**) × 3.0 TBA (111 mg, 81.0 µmol, 1.0 eq.) was dissolved in DMF (4.0 ml), and ETT (31.6 mg, 243 µmol, 3.0 eq.) was added. Afterwards, a solution of phosphoramidite **1** (63.4 mg, 122 µmol, 1.5 eq.) in DMF (4.0 ml) was added, and the resulting solution was stirred for 15 min at room temperature. The solution was cooled to −5 °C, and *m*CPBA (77%, 27.3 mg, 122 µmol, 1.5 eq.) was added. The solution was stirred for 15 min at −5 °C. Subsequently, DBU (158 µl) was added at 0 °C, and the reaction mixture was stirred for 5 min at 0 °C and for 25 min at room temperature. After stirring for 10 min at −20 °C, precipitation was induced by the addition of Et_2O (40 ml). The resulting solid was washed with Et_2O (2 × 15 ml) and dried under high vacuum. The crude product was purified by strong ion-exchange chromatography (Q-Sepharose) using an Äkta system and a $NaClO_4$ buffer (130-150 mM). The product-containing fractions (analyzed via ^{31}P NMR) were precipitated using a $NaClO_4$ solution (0.5 M in acetone, −20 °C, 40 ml), and the precipitate was separated by centrifugation, washed with acetone (−20 °C, 3 × 10 ml), and dried under high vacuum. The product **17** (40.0 mg, 46.2 µmol, 57%) was isolated as a white solid.

^{1}H-NMR (400 MHz, D_2O) δ = 8.59 (s, 1H), 8.31 (s, 1H), 6.24 (d, *J* = 6.4 Hz, 1H), 4.99 (ddd, *J* = 8.5, 5.0, 3.0 Hz, 1H), 4.89 (ddd, *J* = 6.3, 5.1, 1.2 Hz, 1H), 4.62 (ddd, *J* = 2.9, 2.9, 2.9 Hz, 1H), 4.30 (mc, 2H), AB-Signal (δA = 3.97, δB = 3.94, *J*AB = 6.4 Hz, 2H), 2.27-2.23 (m, 1H), 2.18 (ddd, *J* = 7.3, 7.3, 2.6 Hz, 2H), 1.75-1.62 (m, 2H) ppm.

^{31}P{^{1}H}-NMR (162 MHz, D_2O) δ = −5.45 (d, *J* = 22.0 Hz), −10.62 (d, *J* = 21.3 Hz), −10.76 (d, *J* = 18.2 Hz), −11.18 (d, *J* = 17.4 Hz), −22.55 (dd, *J* = 18.3, 18.3 Hz) ppm.

^{13}C{^{1}H}-NMR (101 MHz, D_2O) δ = 155.7, 153.0, 149.4, 140.0, 118.7, 86.7, 84.9, 83.5 (dd, *J* = 9.3, 4.2 Hz), 74.9 (d, *J* = 5.2 Hz), 73.8, 69.1, 65.5 (d, *J* = 5.4 Hz), 65.2 (d, *J* = 5.9 Hz), 28.8 (d, *J* = 7.2 Hz), 14.1 (d, *J* = 2.9 Hz) ppm.

HRMS (ESI-): *m/z* for $C_{15}H_{23}N_5O_{19}P_5^-$ [M-H]$^-$ calcd. 731.9680, found 731.9679.

18

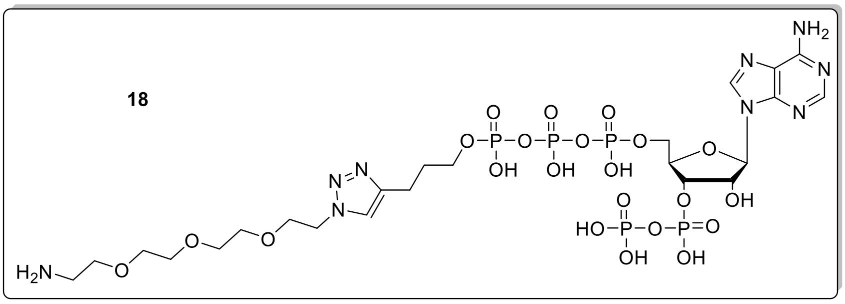

The sodium salt of pentynyl-pppApp (**27**) (20.0 mg, 23.1 µmol, 1.0 eq.) sodium ascorbate (46.0 mg, 231 µmol, 10 eq.) and 1-amino-11-azido-3,6,9-trioxaundecane (11.4 µl, 10.1 mg, 46.2 µmol, 2.0 eq.) were dissolved in water (1.5 ml) and PBS 10× buffer (200 µl) and degassed for 10 min. A premixed solution of tris((1-hydroxy-propyl-1*H*-1,2,3-triazol-4-yl)methyl)amine (THPTA) (5.0 mg, 11.6 µmol, 0.5 eq.) and $CuSO_4$ (115 µl of a 20 mM stock solution) was added, and the mixture was stirred for 1 h at room temperature. A small spatula tip of Chelex 100 was added. Afterwards, H_2O (35 ml) was added. The crude product was purified by strong ion-exchange chromatography (Q-Sepharose) using an Äkta system and a $NaClO_4$ buffer (110-130 mM). The product-containing fractions (analyzed via HPLC-UV) were precipitated using an 8-fold volume of $NaClO_4$ solution (−20 °C, 500 mM in acetone). The resulting solid was separated by centrifugation, washed with acetone (−20 °C, 3 × 10 ml), and dried under high vacuum for 2 h. The product **18** (7.7 mg, 7.1 µmol, 31%) was isolated as a white solid.

^{1}H NMR (400 MHz, D_2O) δ = 8.55 (s, 1H), 8.23 (s, 1H), 7.66 (s, 1H), 6.17 (d, *J* = 6.4 Hz, 1H), 5.04-4.93 (m, 1H), 4.61 (mc, 1H), 4.53 (dd, *J* = 5.0, 5.0 Hz, 2H), 4.30 (mc, 2H), 3.99 – 3.89 (m, 4H), 3.74 (dd, *J* = 5.2, 5.2 Hz, 2H), 3.66-3.58 (m, 6H), 3.58-3.50 (m, 2H), 3.21 (dd, *J* = 5.8, 4.6 Hz, 2H), 2.58 (dd, *J* = 6.5, 6.5 Hz, 2H), 1.77 (dddd, *J* = 6.5, 6.5, 6.5, 6.4 Hz, 2H) ppm.

^{31}P{^{1}H}-NMR (162 MHz, D_2O) δ = −5.60 (d, *J* = 21.9 Hz), −10.76 (d, *J* = 21.9 Hz), −10.83 (d, *J* = 18.2 Hz), −11.24 (d, *J* = 17.1 Hz), −22.70 (dd, *J* = 18.2, 18.2 Hz) ppm.

^{13}C{^{1}H}-NMR (101 MHz, D_2O) δ = 155.5, 152.8, 149.2, 147.4, 139.9, 123.2, 118.4, 86.6, 83.5 (dd, *J* = 9.3, 4.2 Hz), 74.9 (d, *J* = 5.2 Hz), 73.9 (d, *J* = 4.3 Hz), 69.7, 69.5, 69.5, 69.4, 68.7, 66.5, 65.6 (d, *J* = 5.4 Hz), 65.5 (d, *J* = 5.9 Hz), 49.8, 39.0, 29.2 (d, *J* = 7.7 Hz), 20.9 ppm.

HRMS (ESI-): *m/z* for $C_{23}H_{41}N_9O_{22}P_5^-$ [M-H]$^-$ calcd. 950.1060, found 950.1055.

3.6. Synthesis of fluorescence sensor probe

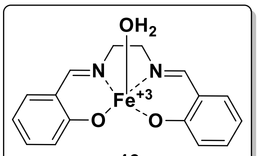

19

Fe–Sal Complex **19** was prepared according to the reported procedure. Analytical data were identical to literature.[7]

4. Spectroscopic studies- MSN sensing

4.1. Materials and Methods

General Spectroscopic Methods

UV/Vis absorption spectra were recorded on a Shimadzu UV-1900i spectrophotometer using quartz cuvettes with a path length of 1 cm. Fluorescence emission spectra were recorded on a JASCO FP-8200 spectrofluorometer or, where indicated, on a Tecan Spark 10M microplate reader using black 96-well plates (Thermo Fisher Scientific, Nunclon). All measurements were performed at room temperature unless stated otherwise. Tris buffer (10 mM, pH 7.4) was used for all spectroscopic experiments. Stock solutions of probe 1 and analytes were prepared in water and diluted immediately prior to use.

UV/Vis Absorption Measurements

UV/Vis absorption experiments were performed using a stock solution of probe **19** (100 µM). The final concentration of 1 in the measurement solution was 100 µM in Tris buffer (10 mM, pH 7.4). Sodium salts of the respective alarmone nucleotides were added from aqueous stock solutions to afford the indicated equivalents. After incubation for 40 min, absorption spectra were recorded in the range of 250–600 nm.

Fluorescence Emission Measurements

Fluorescence measurements were performed using a stock solution of probe **19** (100 µM). The final concentration of 1 in the measurement solution was 50 µM in Tris buffer (10 mM, pH 7.4). Alarmone nucleotides were added from aqueous stock solutions to afford the indicated equivalents. After incubation for 40 min, fluorescence emission spectra were recorded upon excitation at λ_{ex} = 375 nm.

Time-resolved Fluorescence Measurements

For time-resolved fluorescence experiments, probe **19** (50 µM) was incubated with 10 equivalents of the respective alarmone nucleotide in Tris buffer (10 mM, pH 7.4). Fluorescence emission spectra were recorded at defined time points over a period of up to 70 min with excitation at λ_{ex} = 375 nm and λ_{em} = 510 nm.

Fluorescence Titration Experiments

Fluorescence titration experiments were conducted by incremental addition of alarmone nucleotides (1–10 equivalents) to a solution of probe **19** (50 µM) in Tris buffer (10 mM, pH 7.4). After incubation for 40 min, fluorescence emission spectra were recorded at λ_{ex} = 375 nm and λ_{em} = 510 nm.

Selectivity Experiments

Selectivity studies were performed by incubating probe **19** (80 µM) with various phosphate species and nucleotides (5.0 equivalents each) in Tris buffer (10 mM, pH 7.4). Measurements were conducted in black 96-well plates with a total volume of 200 µL per well. Fluorescence emission intensities at 520 nm were recorded after 40 min incubation (λ_{ex} = 375 nm). All measurements were performed in triplicate, and data are reported as mean ± standard deviation.

Plate-Reader Measurements

Plate-reader-based fluorescence measurements were performed using a Tecan Spark 10M microplate reader. Emission spectra and fluorescence intensities were recorded in black 96-well plates at λ_{ex} = 375 nm under otherwise identical buffer and concentration conditions as described above.

4.2. Time-Dependent Fluorescence Response

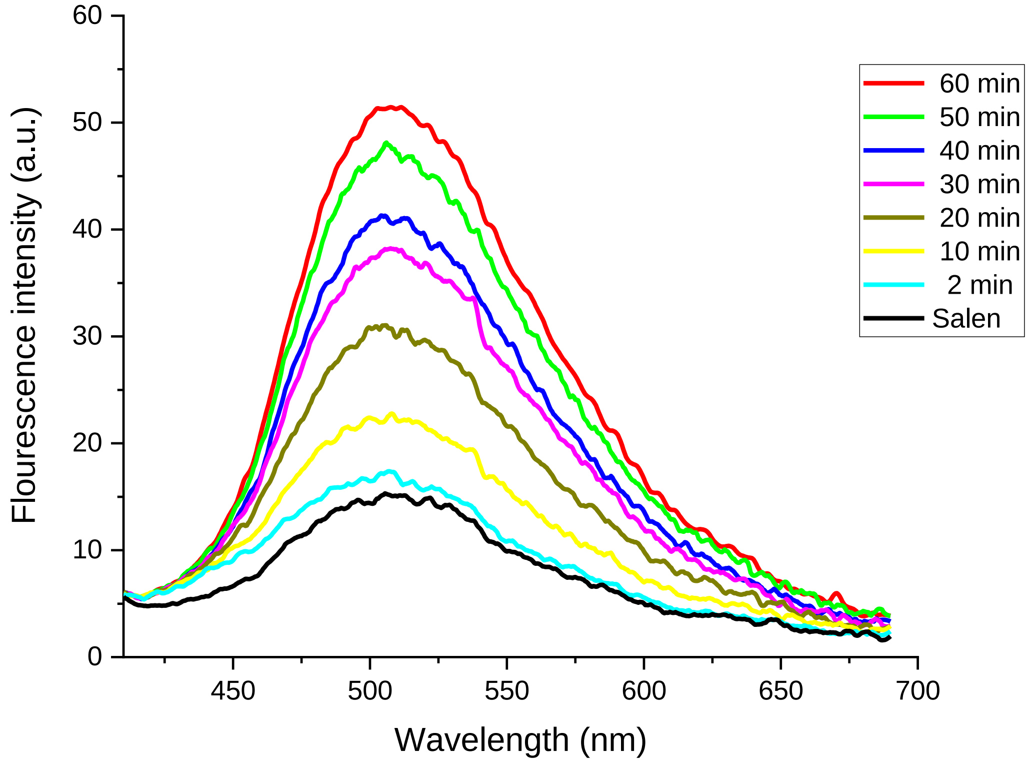

Figure S3. Time-dependent fluorescence emission spectra of probe **19** (50 µM) recorded after incubation with ppGpp (10 equiv) in Tris buffer (10 mM, pH 7.4). Emission spectra were recorded at the indicated time points (2–60 min) upon excitation at λ_{ex} = 375 nm and λ_{em} = 510 nm.

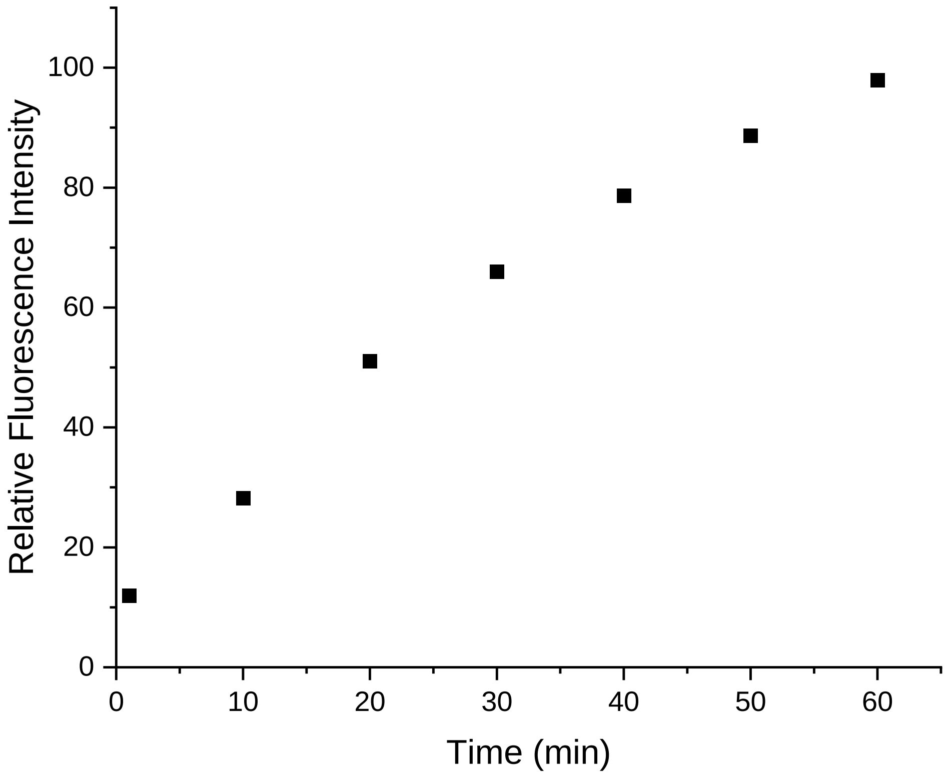

Figure S4. Time-dependent fluorescence emission spectra of probe **19** (50 µM) in the presence of pppApp (10 equiv) in Tris buffer (10 mM, pH 7.4). Spectra were recorded at the indicated time points (2–70 min) upon excitation at λ_{ex} = 375 nm and λ_{em} = 510 nm.

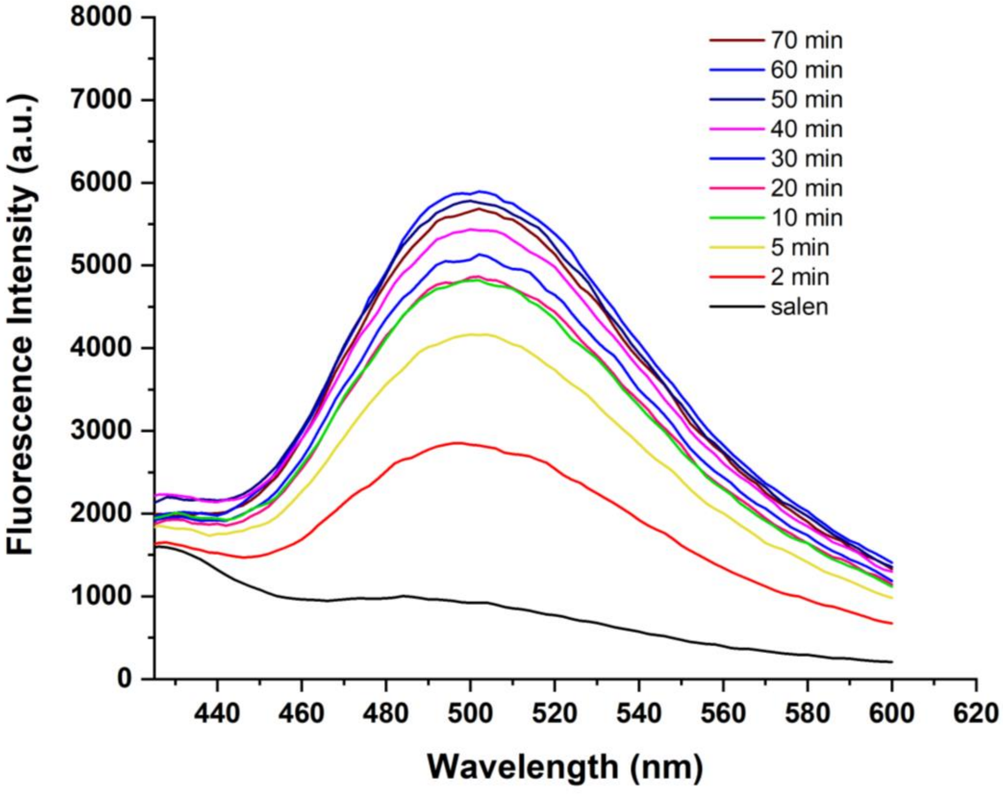

Figure S5. Time-dependent evolution of the fluorescence emission profile of probe **19** (50 µM) in the presence of ppApp (10 equiv), recorded using a plate-reader setup. Emission spectra were collected at the indicated time points (λ_{ex} = 375 nm and λ_{em} = 510 nm).

4.3. Fluorescence Titration and Concentration Dependence

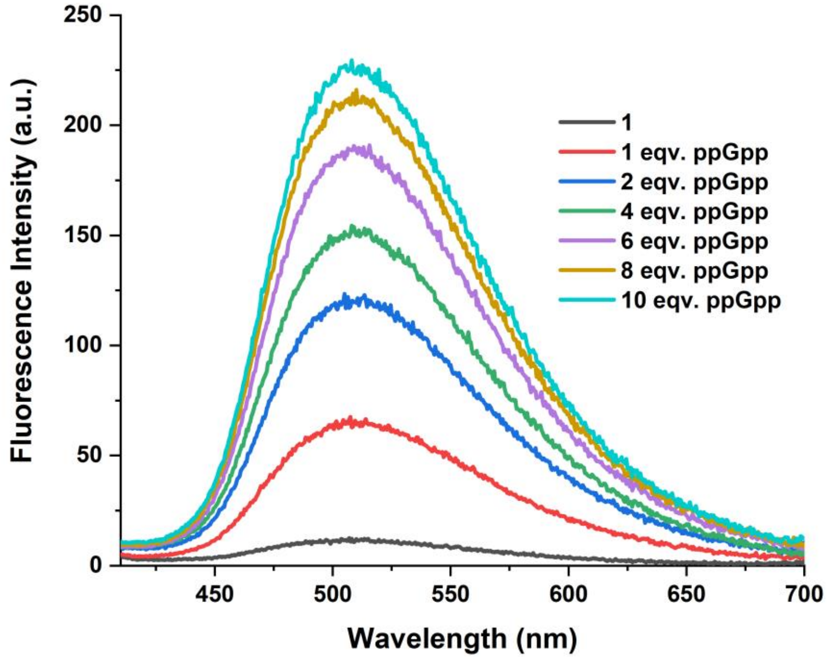

Figure S6. Fluorescence titration of probe **19** (50 µM) with increasing equivalents of ppGpp (1–10 equiv) in Tris buffer (10 mM, pH 7.4). Emission spectra were recorded after 40 min incubation (λ_{ex} = 375 nm and λ_{em} = 510 nm).

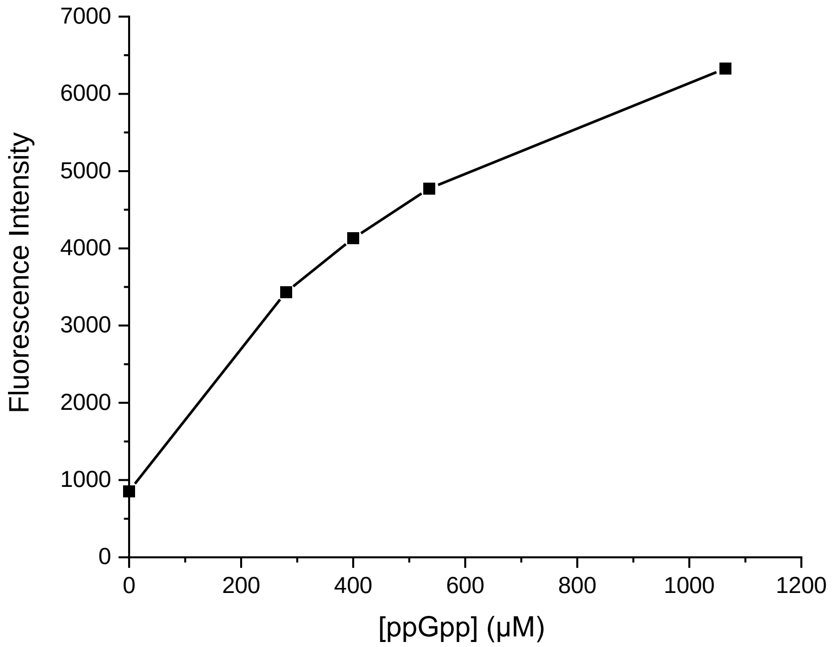

Figure S7. Concentration-dependent fluorescence response of probe **19** (50 μM) toward ppGpp. Fluorescence intensity at 510 nm is plotted as a function of ppGpp concentration after 40 min incubation in Tris buffer (10 mM, pH 7.4) (λ_{ex} = 375 nm and λ_{em} = 510 nm).

4.4. Selectivity toward Phosphate Species

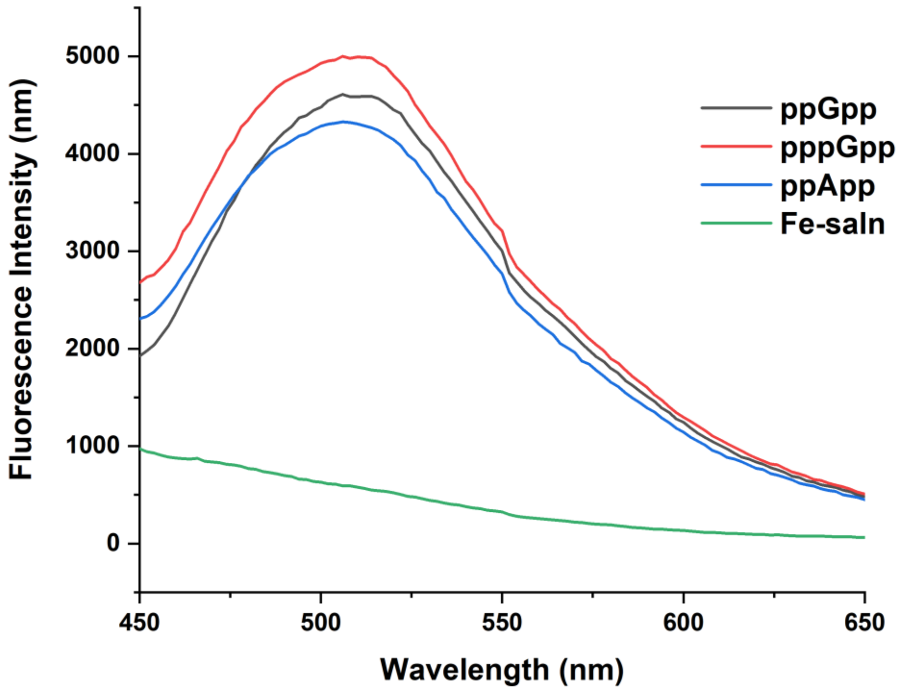

Figure S8. Comparison of fluorescence emission spectra of probe **19** (50 μM) in the presence of ppGpp, pppGpp, and ppApp (10 equiv each) in Tris buffer (10 mM, pH 7.4). The emission spectrum of Fe-Sal alone is shown for comparison (λ_{ex} = 375 nm and λ_{em} = 510 nm).

4.5. Relative fluorescence intensity of Salen complex with different phosphate compounds

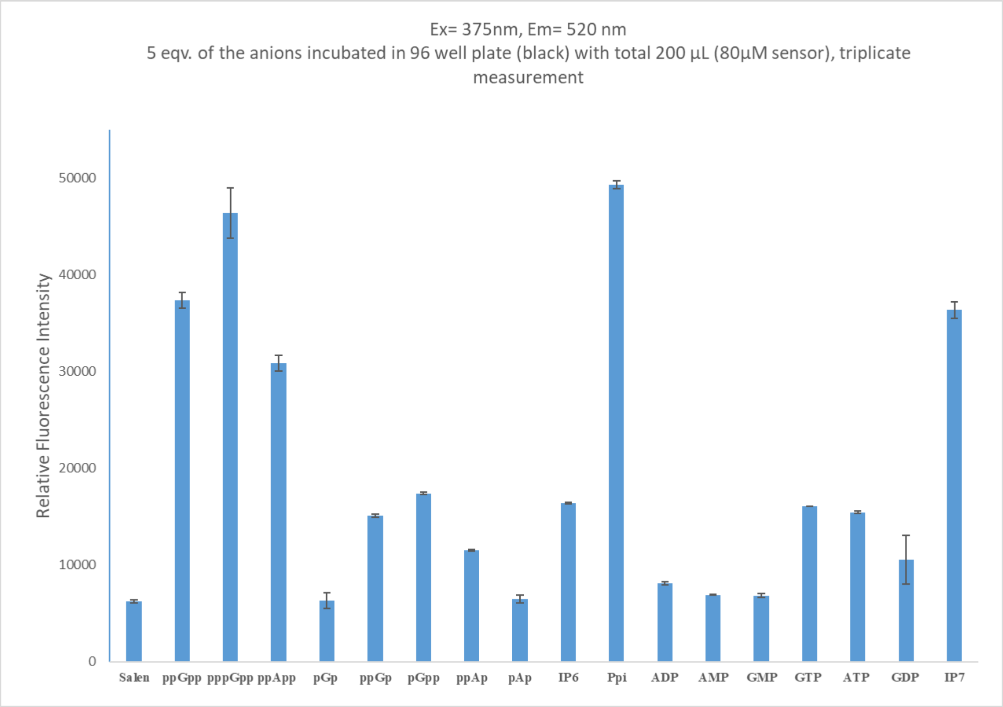

Figure S9. Selectivity of probe **19** toward different analytes. Normalized fluorescence emission intensity at 520 nm of **19** (80 µM) after incubation with various anions (5.0 equiv) in Tris buffer (10 mM, pH 7.4) for 40 min. Measurements were performed in a 96-well plate format (n = 3; mean ± SD).

5. DFT-Studies

General remarks

All DFT calculations have been carried out using Gaussian16.[8] All geometries were optimized in the gas phase using the UB3LYP[9] functional in combination with the def2SVP basis set[10] for all atoms except iron for which the SDD[11] pseudopotentials were applied. High and low spin complexes of iron(III) were considered by a multiplicity of 6 and 2 for all iron complexes in separate calculations. In all cases the ultrafine integral grid was employed (int=grid=ultrafine), moreover, we used the scf=xqc keyword for all structures. During the geometry optimization we considered the solvent water by the use of the IEFPCM model[12] and the dispersion energies were considered by the use of the Grimme D3 correction together with Becke-Johnson damping (keyword EmpiricalDispersion=GD3BJ).[13] Frequency calculations were performed in order to obtain thermal corrections at 298 K. All optimized species showed no imaginary frequencies during vibrational analysis.

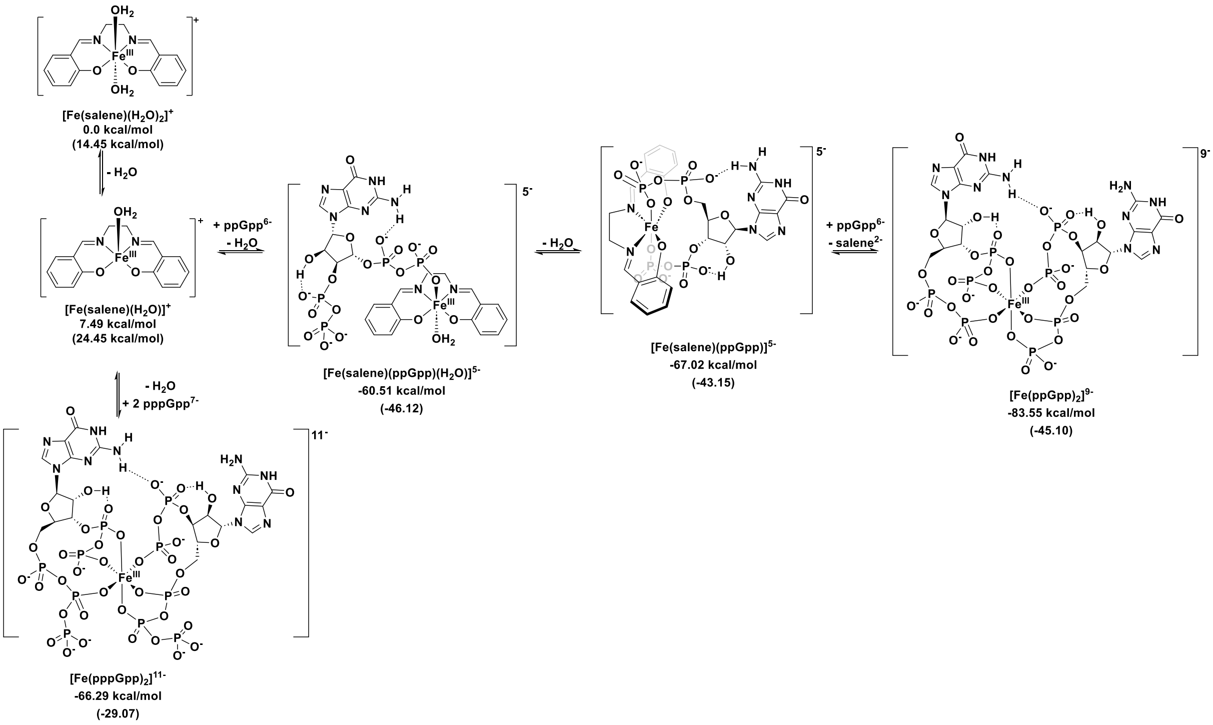

Figure S11 Relative ΔG values (UB3LYP/def2SVP/SDD/D3BJ/IEFPCM=water) of all considered species in kcal/mol. First the ΔG values of the high spin complex is listed and the ΔG values for the low spin complex is reported in brackets.

Atomic coordinates

[Fe(salene)(H_2O)$_2$]$^+$ (multiplicity = 6)

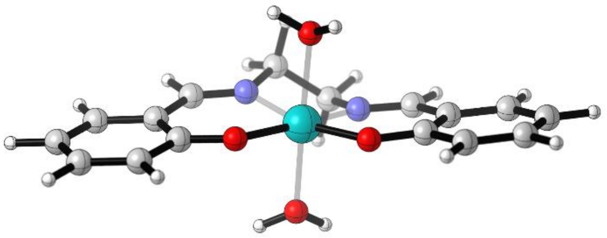

**E/hartree**	-1154.22
**E+zvp/hartree**	-1153.89
**G/hartree**	-1153.95

C	-5.0831880	-2.1106693	-0.1131952
C	-3.7069040	-2.2900803	-0.0561092
C	-2.8325340	-1.1833543	0.0448448
C	-3.4020500	0.1302687	0.0930388
C	-4.8070880	0.2766247	0.0332538
C	-5.6465220	-0.8225903	-0.0700772
H	-5.7340690	-2.9848643	-0.1933702
H	-3.2645090	-3.2873733	-0.0898902
H	-5.2272850	1.2848547	0.0716128
H	-6.7286800	-0.6902523	-0.1159502
O	-1.5360410	-1.3902093	0.0927898
C	-2.6055610	1.3292027	0.2148838
H	-3.1728020	2.2726947	0.2617588
N	-1.3178640	1.3830927	0.2786358
C	-0.6337830	2.6621247	0.4256688
H	-1.2896440	3.5091587	0.1696198
H	-0.3262740	2.7759327	1.4792048
C	0.6098570	2.6450867	-0.4617872
H	1.2633310	3.5044677	-0.2434432
H	0.3012090	2.7079147	-1.5190252
C	2.5890230	1.3242637	-0.2365492
H	3.1532650	2.2657937	-0.3329202
N	1.2992440	1.3757627	-0.2631652
C	3.3889720	0.1296307	-0.1005062
C	4.7949000	0.2777487	-0.0640642
C	2.8205090	-1.1837393	-0.0241522
C	5.6360190	-0.8191753	0.0457408
H	5.2137850	1.2853597	-0.1258282
C	3.6973570	-2.2883163	0.0815158
C	5.0738770	-2.1068943	0.1172578
H	6.7186630	-0.6859973	0.0741288
H	3.2561430	-3.2851603	0.1366428
H	5.7265900	-2.9792793	0.2022798
O	1.5242970	-1.3908873	-0.0528932
Fe	-0.0057420	-0.2728533	0.0302098
O	0.2233040	-0.2492283	2.1831728
H	-0.4343880	0.2305527	2.7101578
H	1.0874240	-0.0523103	2.5769588
H	0.5231940	-0.2212583	-2.6643442
H	-0.8616710	-0.8888363	-2.4688632
O	-0.2843660	-0.1872073	-2.1272442

[Fe(salene)(H_2O)$_2$]$^+$ (multiplicity = 2)

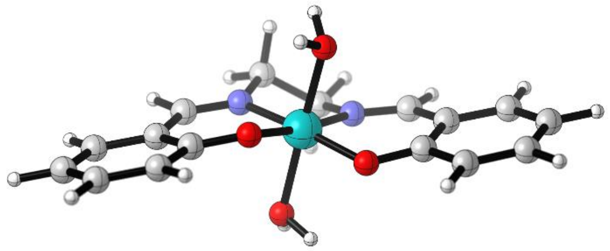

**E/hartree**	-1154.20
**E+zvp/hartree**	-1153.88
**G/hartree**	-1153.92

C	-4.7250725	-2.2854344	0.0081309
C	-3.3538285	-2.3406134	0.2016479
C	-2.5687955	-1.1593544	0.2212689
C	-3.2447905	0.0955806	0.0590259
C	-4.6507295	0.1164526	-0.1225851
C	-5.3890975	-1.0523754	-0.1593831
H	-5.2992555	-3.2152414	-0.0135801
H	-2.8395675	-3.2940934	0.3355579
H	-5.1460005	1.0843356	-0.2342941
H	-6.4696775	-1.0224934	-0.3080271
O	-1.2796565	-1.2606314	0.4254949
C	-2.5522925	1.3484766	0.1519459
H	-3.1716695	2.2562076	0.1800039
N	-1.2670475	1.4875216	0.2291809
C	-0.6435955	2.7982206	0.4099909
H	-1.3206815	3.6105476	0.1069749
H	-0.4068865	2.9353036	1.4787259
C	0.6436835	2.7981216	-0.4098691
H	1.3207525	3.6104656	-0.1068611
H	0.4071365	2.9350346	-1.4786571
C	2.5523565	1.3484376	-0.1518771
H	3.1716555	2.2562116	-0.1802651
N	1.2670775	1.4874066	-0.2287631
C	3.2449555	0.0956166	-0.0590111
C	4.6509405	0.1165136	0.1222229
C	2.5688805	-1.1593234	-0.2209991
C	5.3893055	-1.0523174	0.1589319
H	5.1462355	1.0844096	0.2337309
C	3.3539365	-2.3405884	-0.2015211
C	4.7252235	-2.2853834	-0.0083511
H	6.4699245	-1.0224454	0.3072979
H	2.8396595	-3.2940864	-0.3352501
H	5.2994115	-3.2151904	0.0132859
O	1.2797485	-1.2605504	-0.4249641
Fe	-0.0000745	0.0477016	0.0002109
O	0.5529645	-0.0743444	1.9237229
H	-0.0146995	-0.7640034	2.3118279
H	0.4437125	0.7113556	2.4832339
H	-0.4462235	0.7116836	-2.4829731
H	0.0151285	-0.7629884	-2.3119551
O	-0.5530475	-0.0741444	-1.9232261

$[Fe(salene)(H_2O)]^+$ (multiplicity = 6)

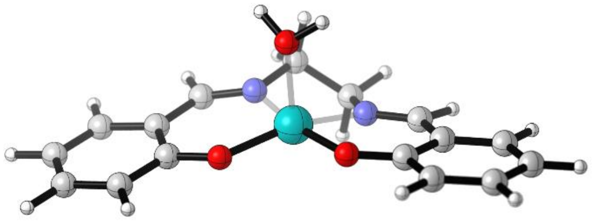

E/hartree	-1077.82
**E+zvp/hartree**	-1077.52
**G/hartree**	-1077.57

C	4.9970999	-2.1924309	0.4441483
C	3.6190359	-2.3368549	0.3455763
C	2.7829809	-1.2094209	0.1837273
C	3.3884869	0.0869681	0.1305053
C	4.7940099	0.1987911	0.2410183
C	5.5972999	-0.9210029	0.3924053
H	5.6209009	-3.0813939	0.5653323
H	3.1481309	-3.3205449	0.3869023
H	5.2420339	1.1948091	0.2023843
H	6.6806169	-0.8179989	0.4724313
O	1.4826309	-1.3826349	0.0855683
C	2.6230529	1.2999691	-0.0158947
H	3.2017179	2.2368671	0.0053423
N	1.3409379	1.3731821	-0.1631637
C	0.6721329	2.6640761	-0.2735927
H	1.3321039	3.4947191	0.0208243
H	0.3748779	2.8183261	-1.3248037
C	-0.5744741	2.6152911	0.6068783
H	-1.2304111	3.4789791	0.4162633
H	-0.2699981	2.6421061	1.6669463
C	-2.5528041	1.2877521	0.4516983
H	-3.1046311	2.2151071	0.6727173
N	-1.2639891	1.3501911	0.3606993
C	-3.3614121	0.1030901	0.3071423
C	-4.7628391	0.2338321	0.4425933
C	-2.7997301	-1.1914219	0.0662473
C	-5.6020311	-0.8660839	0.3469503
H	-5.1790801	1.2267961	0.6296883
C	-3.6712261	-2.3000929	-0.0215587
C	-5.0440091	-2.1365839	0.1147743
H	-6.6815241	-0.7486579	0.4540663
H	-3.2322441	-3.2830609	-0.2008807
H	-5.6969331	-3.0096979	0.0414773
O	-1.5055711	-1.3737389	-0.0703877
Fe	0.0040539	-0.2550589	-0.1783927
O	-0.0647711	-0.2217179	-2.2744837
H	0.6074759	0.2468081	-2.7941437
H	-0.9119001	-0.1192639	-2.7370077

$[Fe(salene)(H_2O)]^+$ (multiplicity = 2)

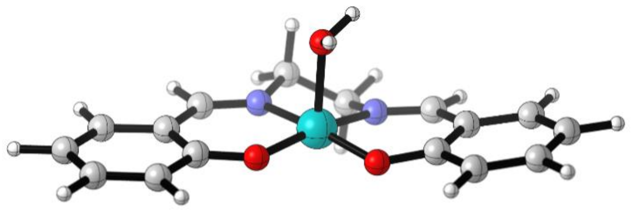

E/hartree	-1077.80
**E+zvp/hartree**	-1077.50
**G/hartree**	-1077.55

C	-4.8042265	-2.2656975	-0.1915156
C	-3.4218135	-2.3295815	-0.2545276
C	-2.6379185	-1.1488735	-0.2156076
C	-3.3160955	0.1081615	-0.1121316
C	-4.7331905	0.1380245	-0.0539506
C	-5.4747245	-1.0277285	-0.0909416
H	-5.3835435	-3.1919585	-0.2187276
H	-2.9006265	-3.2851725	-0.3331106
H	-5.2311875	1.1075605	0.0244054
H	-6.5641905	-0.9939435	-0.0417736
O	-1.3348355	-1.2567265	-0.2879156
C	-2.6020545	1.3458325	-0.0611136
H	-3.2063405	2.2600455	0.0173854
N	-1.3105505	1.4784875	-0.0852166
C	-0.6975275	2.8009635	0.0815514
H	-1.3536275	3.5952325	-0.3027316
H	-0.5407425	2.9769405	1.1590164
C	0.6373915	2.7779925	-0.6447236
H	1.2902485	3.6046165	-0.3283406
H	0.4757645	2.8597055	-1.7321596
C	2.5478205	1.3498495	-0.3099896
H	3.1577895	2.2613115	-0.3771566
N	1.2578125	1.4767655	-0.3734986
C	3.2471305	0.1063725	-0.1873506
C	4.6550335	0.1267055	-0.0229726
C	2.5652815	-1.1483925	-0.3021506
C	5.3828865	-1.0470045	0.0431584
H	5.1579315	1.0939225	0.0505954
C	3.3364055	-2.3357335	-0.2489826
C	4.7099075	-2.2816845	-0.0731236
H	6.4653935	-1.0207035	0.1772204
H	2.8131305	-3.2886465	-0.3438826
H	5.2796365	-3.2129545	-0.0254336
O	1.2691005	-1.2440995	-0.4816746
Fe	-0.0215495	0.0494425	-0.1672076
O	0.1559365	-0.0335575	1.8532154
H	0.8210225	0.5304415	2.2811324
H	0.3091205	-0.9359165	2.1802314

[Fe(salene)(ppGpp)(H_2O)]$^{5-}$ (multiplicity = 6)

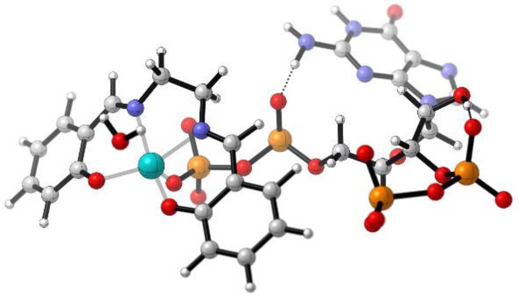

**E/hartree**	-4383.10
**E+zvp/hartree**	-4382.51
**G/hartree**	-4382.60

C	8.5532244	1.2887438	1.6644977
C	7.8270254	0.1287158	1.4336527
C	6.7265124	0.1060208	0.5354537
C	6.4041994	1.3305178	-0.1453853
C	7.1709384	2.4905918	0.1073757
C	8.2298574	2.4885398	1.0032617
H	9.3878474	1.2667308	2.3707547
H	8.0774184	-0.8028842	1.9460977
H	6.9088094	3.4090978	-0.4257313
H	8.8048394	3.3977918	1.1881367
O	6.0709764	-1.0075642	0.3466857
C	5.3508884	1.4251778	-1.1330763
H	5.2783754	2.3896578	-1.6623053
N	4.5354554	0.4825778	-1.4474923
C	3.5299414	0.6688078	-2.4781283
H	3.4852454	1.7164268	-2.8206463
H	3.8043884	0.0437888	-3.3469783
C	2.1750404	0.2279268	-1.9287633
H	1.4324944	0.1390328	-2.7390513
H	1.8077794	0.9758138	-1.2115323
C	1.3513484	-1.8464642	-1.0861133
H	0.3938524	-1.5971342	-1.5726853
N	2.3394014	-1.0313022	-1.2170873
C	1.3593024	-3.0907132	-0.3457333
C	0.1742254	-3.8627882	-0.3638353
C	2.5002104	-3.5650262	0.3878467
C	0.0804014	-5.0792042	0.3021567
H	-0.7139796	-3.4891962	-0.8878343
C	2.3823134	-4.8133162	1.0492357
C	1.2021504	-5.5478872	1.0065697
H	-0.8722156	-5.6170992	0.2814997
H	3.2531274	-5.1737962	1.6020687
H	1.1516374	-6.5028382	1.5389237
O	3.6278674	-2.9043972	0.4708377
Fe	4.1602414	-1.1336662	-0.1015113
O	4.9741474	-2.3606052	-1.9309413
H	5.4536114	-1.7494342	-2.5101283
H	4.2329064	-2.6831902	-2.4655643
C	-5.3341806	1.0779028	0.9935107
O	-4.1750666	0.8054648	1.7431717
C	-3.5931366	-0.4496012	1.3077227
C	-4.5837676	-1.0471692	0.3135767
C	-5.2426106	0.2114828	-0.2724603
H	-6.2523256	0.7874538	1.5368797
H	-3.5065416	-1.1203892	2.1721807
H	-4.0685336	-1.6655062	-0.4375563
H	-4.5088436	0.6937018	-0.9460873
O	-5.5556906	-1.8161172	1.0174977
O	-6.4763856	0.0430338	-0.8984673
H	-6.5930416	-0.9426842	-1.0772893
C	-2.2301396	-0.2592512	0.6659997
H	-2.3181686	0.3242828	-0.2596563
H	-1.8634406	-1.2688102	0.4211147
C	-4.5363756	3.3254578	0.1242187
C	-6.5064416	3.3122118	1.1296627
C	-5.1161466	4.6035678	0.1569077
H	-7.3643526	2.8915108	1.6551357
C	-4.3904986	5.7021878	-0.4178553
C	-2.6555466	3.9651588	-0.9101663
H	-2.5750666	5.9949358	-1.3336553
N	-5.4453926	2.5036468	0.7541397
N	-6.3481226	4.5658618	0.7867687
N	-3.3522136	2.9637268	-0.3957523
N	-3.1456146	5.2578858	-0.9273583
N	-1.4498066	3.7316978	-1.4585943
H	-0.9717626	2.8440918	-1.1486603
H	-0.8604346	4.5265148	-1.6767033
O	-4.6990336	6.8869448	-0.5041983
O	-1.3340616	0.3959408	1.5567357
P	-0.1923226	1.4527848	1.0018097
O	1.1388094	0.5924778	1.4358217
P	-6.2766096	-3.0803742	0.2057167
O	-7.3997356	-3.5549052	1.0930717
O	-5.1462376	-4.2129762	0.0875987
O	-6.6169506	-2.5328412	-1.1917813
O	2.8981564	2.2853048	0.5478337
O	-0.2861596	2.7310398	1.7918177
P	2.6915564	1.2393628	1.6290487
O	2.8639914	1.6188228	3.0830657
P	-3.3730476	-4.2118232	0.1138567
O	-3.0225296	-3.6142392	1.4773557
O	-3.1058966	-5.7146412	-0.0505373
O	-2.9938176	-3.3569102	-1.1171243
O	-0.2778486	1.4935628	-0.5255413
O	3.5395784	-0.0972312	1.3326957

[Fe(salene)(ppGpp)(H_2O)]$^{5-}$ (multiplicity = 2)

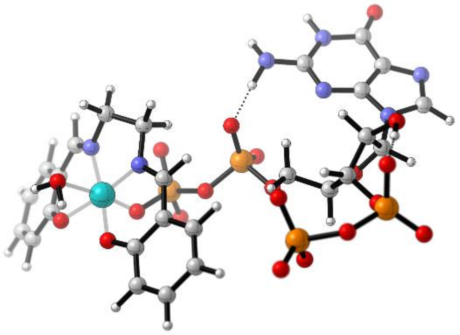

**E/hartree**	-4383.08455
**E+zvp/hartree**	-4382.494343
**G/hartree**	-4382.580775

C	-8.4969596	0.8247193	-1.9673212
C	-7.7032076	-0.2310747	-1.5464212
C	-6.6360426	-0.0423907	-0.6232482
C	-6.4322296	1.2906243	-0.1227992
C	-7.2699536	2.3423313	-0.5625302
C	-8.2853536	2.1308883	-1.4815182
H	-9.2977906	0.6385933	-2.6885002
H	-7.8673116	-1.2431307	-1.9233292
H	-7.0977766	3.3440303	-0.1583092
H	-8.9144726	2.9573283	-1.8173712
O	-5.9240636	-1.0682007	-0.2556692
C	-5.4473726	1.5812593	0.8878478
H	-5.4951206	2.5768583	1.3528408
N	-4.5461446	0.7591313	1.2968068
C	-3.6365326	1.0820803	2.3864938
H	-3.6073386	2.1663553	2.5749698
H	-4.0130226	0.5851733	3.2972068
C	-2.2563276	0.5480213	2.0248748
H	-1.6312906	0.4236283	2.9231108
H	-1.7460896	1.2351183	1.3380268
C	-1.4373776	-1.5468137	1.2080028
H	-0.4815986	-1.2646557	1.6717488
N	-2.4302106	-0.7304097	1.3327218
C	-1.4556256	-2.8103717	0.5158868
C	-0.2618876	-3.5749887	0.5014698
C	-2.6389466	-3.3309167	-0.1119472
C	-0.2005546	-4.8237967	-0.1010022
H	0.6567904	-3.1869627	0.9580848
C	-2.5472766	-4.6140537	-0.7166502
C	-1.3618526	-5.3355697	-0.7108852
H	0.7486424	-5.3693607	-0.1037232
H	-3.4490216	-5.0103537	-1.1897362
H	-1.3349886	-6.3175947	-1.1936532
O	-3.7879326	-2.7082837	-0.1373092
Fe	-4.1220746	-0.9026937	0.4389248
O	-4.9455066	-1.9139747	2.1219358
H	-4.7265836	-2.8121337	1.8133558
H	-5.8873506	-1.8217447	1.8937638
C	5.4049874	0.9635783	-1.0001192
O	4.2171044	0.7037953	-1.7084362
C	3.6168024	-0.5275327	-1.2258092
C	4.6379054	-1.1368267	-0.2749632
C	5.3480484	0.1063803	0.2754038
H	6.2989364	0.6565843	-1.5735812
H	3.4665214	-1.2147117	-2.0678452
H	4.1417934	-1.7439497	0.4961168
H	4.6573904	0.6097573	0.9788528
O	5.5648434	-1.9244447	-1.0177212
O	6.6032184	-0.0960537	0.8484638
H	6.6926904	-1.0826287	1.0308338
C	2.2932084	-0.2877877	-0.5241202
H	2.4319814	0.3671203	0.3459328
H	1.9410454	-1.2734117	-0.1794892
C	4.6708434	3.2330283	-0.1258482
C	6.6250894	3.1692773	-1.1601232
C	5.2845704	4.4951723	-0.1648142
H	7.4636504	2.7267003	-1.6986542
C	4.5989414	5.6120723	0.4234508
C	2.8217554	3.9238533	0.9335928
H	2.8042644	5.9539833	1.3630988
N	5.5480854	2.3888303	-0.7711832
N	6.5054864	4.4259553	-0.8127612
N	3.4839004	2.9035603	0.4100898
N	3.3492224	5.2018533	0.9494908
N	1.6133914	3.7243623	1.4882098
H	1.1072234	2.8463063	1.1961378
H	1.0518674	4.5333303	1.7260708
O	4.9428574	6.7871283	0.5086248
O	1.3523724	0.2991283	-1.4181162
P	0.2255294	1.3860553	-0.8955482
O	-1.1154836	0.5075673	-1.2470472
P	6.2549844	-3.2191287	-0.2320312
O	7.3335864	-3.7387197	-1.1488052
O	5.0770064	-4.2996177	-0.0819252
O	6.6535934	-2.6890957	1.1571228
O	-2.8427676	2.2983473	-0.5133092
O	0.3073854	2.6198273	-1.7547662
P	-2.6640196	1.1519133	-1.4928842
O	-2.8233176	1.4073023	-2.9766792
P	3.3065104	-4.2344267	-0.1698932
O	3.0216884	-3.6194777	-1.5411232
O	2.9763004	-5.7273697	-0.0258122
O	2.9189084	-3.3728007	1.0543438
O	0.3454004	1.5077053	0.6266678
O	-3.5475476	-0.1491607	-1.1352432

[Fe(salene)(ppGpp)]$^{5-}$ (multiplicity = 6)

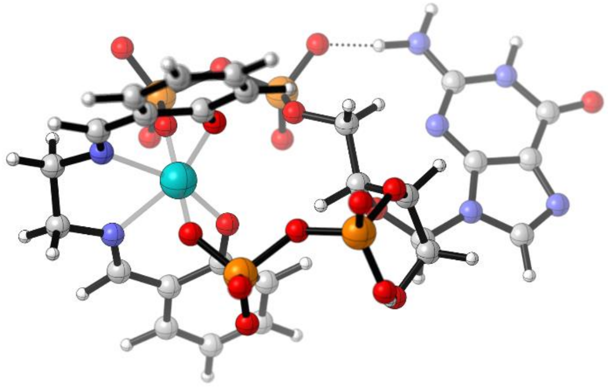

**E/hartree**	-4306.73
**E+zvp/hartree**	-4306.17
**G/hartree**	-4306.25

C	3.4840431	1.0220411	1.4383818
O	2.6321501	0.0688821	0.8847438
C	1.6819911	0.6772711	-0.0273282
C	2.1862281	2.1173521	-0.1690432
C	2.8065751	2.3865641	1.2195568
H	3.5940171	0.8341531	2.5182988
H	0.6912431	0.6570741	0.4461678
H	3.0324891	2.1125521	-0.8810122
O	1.2586501	3.0221261	-0.7124672
O	1.8892321	2.5419071	2.2580328
H	1.2811291	3.3084731	2.0047408
C	1.6130941	-0.0424639	-1.3617922
H	2.6275071	-0.3130319	-1.6888012
H	1.1849171	0.6836491	-2.0759212
C	5.3559461	0.1267181	-0.1370252
C	5.8935681	1.7461691	1.2758568
C	6.7114711	0.4923851	-0.2384912
H	5.7507471	2.4833371	2.0661988
C	7.5480961	-0.1865539	-1.1888992
C	5.4674381	-1.4571659	-1.7316292
H	7.3493601	-1.6779909	-2.5874422
N	4.8382011	0.9549521	0.8420098
N	7.0188211	1.5010651	0.6537438
N	4.7195521	-0.8212649	-0.8438522
N	6.8088981	-1.1632899	-1.8970062
N	4.9247991	-2.4158749	-2.4948582
H	3.8885941	-2.6091739	-2.4373332
H	5.4800271	-2.8703939	-3.2079452
O	8.7401831	-0.0240599	-1.4346752
O	0.7560041	-1.1635159	-1.3504432
P	1.2720351	-2.7332319	-1.3378702
O	-0.1153239	-3.4242539	-1.8404552
P	-0.0572059	3.7581071	0.0094968
O	0.4258821	4.4071641	1.3106708
O	-0.9670569	2.4575111	0.3363098
O	-0.6276649	4.6306361	-1.0721212
O	-1.4099619	-4.4582129	0.1892358
O	1.6743481	-3.1719309	0.0470508
P	-1.6348009	-3.7130879	-1.1159162
O	-2.1908729	-2.2498209	-0.8789672
P	-2.3584019	2.3883311	1.3465878
O	-3.1321629	1.1768261	0.6784278
O	-1.8691869	2.0690911	2.7493248
O	-3.1009889	3.6975811	1.1275478
O	2.2738421	-2.9050809	-2.4809732
O	-2.4154859	-4.4077139	-2.2193462
Fe	-2.6200499	-0.5799059	-0.0432562
C	-3.4257369	3.1398931	-3.9050512
C	-2.6007479	2.2743401	-3.2056722
C	-3.1220059	1.2052121	-2.4134472
C	-4.5588459	1.0677741	-2.3869142
C	-5.3681449	1.9569411	-3.1303122
C	-4.8277129	2.9913451	-3.8795442
H	-2.9787589	3.9528631	-4.4852242
H	-1.5175109	2.4067861	-3.2130642
H	-6.4530549	1.8136561	-3.1011682
H	-5.4714229	3.6734401	-4.4385892
O	-2.3196499	0.3969051	-1.8103552
C	-5.2159229	-0.0003599	-1.6687052
H	-6.2944859	-0.1260029	-1.8781652
N	-4.6504359	-0.7993839	-0.8379552
C	-5.3835839	-1.8693419	-0.2007292
H	-6.4588339	-1.8535889	-0.4543002
H	-4.9546709	-2.8261749	-0.5434712
C	-5.1827389	-1.7398639	1.3084368
H	-5.6066729	-2.6110499	1.8385808
H	-5.7234039	-0.8402429	1.6550378
C	-3.2946889	-1.9627649	2.7172528
H	-3.9789619	-2.4655339	3.4256518
N	-3.7669669	-1.5678929	1.5872658
C	-1.9405499	-1.8115319	3.1911488
C	-1.6439549	-2.3157699	4.4810518
C	-0.9084019	-1.1635029	2.4201468
C	-0.3810859	-2.2096409	5.0396708
H	-2.4497419	-2.8002099	5.0417728
C	0.3870581	-1.0766919	3.0217768
C	0.6347261	-1.5819999	4.2859268
H	-0.1743229	-2.6043759	6.0365438
H	1.1819151	-0.6170239	2.4336228
H	1.6416441	-1.4959509	4.7064368
O	-1.0952259	-0.6881229	1.2412558
H	3.5409811	3.2139651	1.1735758

[Fe(salene)(ppGpp)]$^{5-}$ (multiplicity = 2)

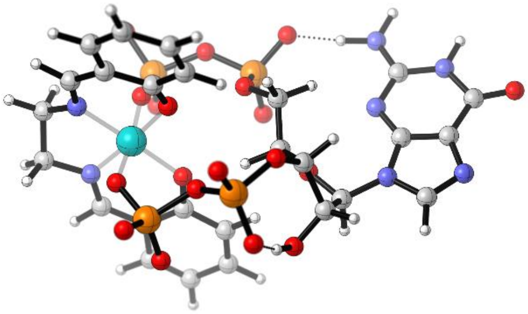

**E/hartree**	-4306.70029
**E+zvp/hartree**	-4306.135215
**G/hartree**	-4306.214071

C	3.2389268	1.0727743	1.5148979
O	2.3811008	0.0976223	1.0230169
C	1.4773748	0.6365483	0.0158189
C	1.9770318	2.0753583	-0.1817691
C	2.5649078	2.4185923	1.2074329
H	3.3465198	0.9484483	2.6043319
H	0.4550308	0.6228063	0.4166509
H	2.8402088	2.0467693	-0.8731711
O	1.0683158	2.9693643	-0.7787461
O	1.6262418	2.6225563	2.2178799
H	1.0368738	3.3904363	1.9198859
C	1.5072488	-0.1987237	-1.2529961
H	2.5452358	-0.4895757	-1.4776921
H	1.1291718	0.4472083	-2.0622651
C	5.1565628	-0.0022777	0.1147889
C	5.6261388	1.8530853	1.2283489
C	6.5038348	0.3787503	-0.0326111
H	5.4533058	2.7109103	1.8778599
C	7.3741588	-0.4311377	-0.8393921
C	5.3327658	-1.8270357	-1.1896941
H	7.2328928	-2.1378237	-1.9745371
N	4.5979988	0.9703153	0.9242319
N	6.7688388	1.5355933	0.6738519
N	4.5577698	-1.0758887	-0.4245031
N	6.6708698	-1.5319257	-1.3821681
N	4.8241638	-2.9117127	-1.7926821
H	3.7734798	-3.0518017	-1.8027391
H	5.3946038	-3.4268657	-2.4510521
O	8.5671638	-0.2827227	-1.0911061
O	0.6584538	-1.3227707	-1.2017371
P	1.1100688	-2.8800317	-0.9389561
O	-0.2685082	-3.5967037	-1.4209121
P	-0.2392722	3.7714133	-0.1119371
O	0.2413748	4.4703533	1.1676069
O	-1.1825112	2.5194053	0.2687329
O	-0.7649572	4.6276253	-1.2282731
O	-1.7041472	-4.3971587	0.6115599
O	1.4217278	-3.1398017	0.5145059
P	-1.8427022	-3.7177117	-0.7458401
O	-2.3383162	-2.2363897	-0.6575471
P	-2.6155722	2.4709623	1.1823399
O	-3.3818962	1.2315703	0.5356599
O	-2.2090512	2.2388383	2.6282109
O	-3.3757352	3.7508683	0.8601749
O	2.1699118	-3.2541467	-1.9817811
O	-2.6006852	-4.4717787	-1.8331011
Fe	-2.7926742	-0.5025377	0.0137639
C	-1.8682372	2.5797273	-4.2262051
C	-1.4129212	1.8860263	-3.1222981
C	-2.2602802	0.9892863	-2.3863231
C	-3.6272132	0.8812153	-2.8519621
C	-4.0512242	1.6018633	-3.9966561
C	-3.1993042	2.4451233	-4.6870591
H	-1.1860272	3.2600283	-4.7458071
H	-0.3993142	2.0315543	-2.7489441
H	-5.0864202	1.4758363	-4.3302411
H	-3.5446422	2.9966703	-5.5640831
O	-1.7685962	0.3240543	-1.4104581
C	-4.5676872	-0.0047277	-2.2250151
H	-5.5299032	-0.1525737	-2.7427921
N	-4.3617872	-0.6440517	-1.1267551
C	-5.3034252	-1.6180687	-0.6086881
H	-6.3034222	-1.5125587	-1.0613421
H	-4.9031452	-2.6193417	-0.8385271
C	-5.3442032	-1.4242297	0.9021329
H	-5.8532282	-2.2621427	1.4061579
H	-5.8972502	-0.4970657	1.1345409
C	-3.6521712	-1.5672257	2.5782839
H	-4.4351842	-1.9853207	3.2317639
N	-3.9687672	-1.2750337	1.3620219
C	-2.3602542	-1.3887177	3.1745999
C	-2.1981442	-1.7746027	4.5300879
C	-1.2514462	-0.8173947	2.4467729
C	-0.9955942	-1.6248797	5.1954809
H	-3.0626172	-2.1997537	5.0501629
C	-0.0174972	-0.6849217	3.1677219
C	0.0989498	-1.0727477	4.4877309
H	-0.8903592	-1.9260057	6.2398769
H	0.8310538	-0.2718697	2.6220169
H	1.0604418	-0.9501927	4.9966209
O	-1.2946762	-0.4412387	1.2218969
H	3.2902548	3.2516233	1.1309429

[Fe(ppGpp)$_2$]$^{9-}$ (multiplicity = 6)

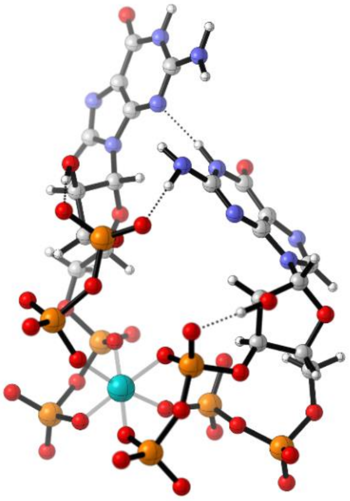

**E/hartree**	-6734.19
**E+zvp/hartree**	-6733.60
**G/hartree**	-6733.70

C	1.3825572	4.1704655	0.8791480
O	2.4833342	4.8052585	0.3033210
C	3.5692482	3.8689005	0.0431360
C	3.0905202	2.5263275	0.6318540
C	1.9077602	2.9150045	1.5568700
H	0.9205942	4.8451645	1.6171550
H	4.4673752	4.2136765	0.5773330
H	2.7593452	1.8705175	-0.1883680
H	1.1442422	2.1256575	1.5961170
O	4.1211402	1.8866445	1.3622560
O	2.3337162	3.2709565	2.8481370
H	2.6296212	2.4052835	3.2394030
C	3.8144562	3.9031165	-1.4776930
H	3.4981622	4.9061445	-1.8098120
H	3.1790612	3.1390655	-1.9544320
C	-0.7934958	3.1523325	0.0226050
C	0.3045512	4.4081745	-1.4282250
C	-1.5047298	3.3003895	-1.1774190
H	1.1117622	5.0293445	-1.8079170
C	-2.8041618	2.6912175	-1.2861000
C	-2.3325668	1.8680525	1.0185240
H	-4.0318358	1.5109675	-0.0843250
N	0.3720952	3.8652305	-0.1549310
N	-0.7952138	4.0854585	-2.0666690
N	-1.1606838	2.4690785	1.1252700
N	-3.1234108	1.9944615	-0.1016800
N	-2.8124198	1.1152745	2.0399760
H	-3.6082298	0.5224455	1.8128060
H	-2.0641478	0.6417635	2.5987880
O	-3.6011708	2.7153095	-2.2207180
O	5.1490022	3.7614095	-1.9218710
P	6.2243502	2.5679975	-1.4603640
O	5.2656242	1.2621615	-1.2879060
P	3.7712412	0.4520265	2.0874390
O	2.9906822	0.7690575	3.3610450
O	5.2388292	-0.0307105	2.4802260
O	3.0781002	-0.4584815	1.0744440
O	4.2728482	-1.0398175	-1.5002690
O	6.7724532	2.9321235	-0.0978860
P	4.2357842	0.3340735	-2.2695800
O	4.8841932	0.2081885	-3.6403810
P	6.3084622	-1.2791835	1.9300710
O	6.6329722	-2.0433065	3.2015710
O	5.4134622	-2.1144845	0.9447480
O	7.4305172	-0.5405425	1.2240350
O	7.1774142	2.4413765	-2.6262280
O	2.8711552	1.0316515	-2.2250360
C	-3.4631258	-1.5133275	-0.5547650
O	-2.6255838	-1.6394545	-1.6729760
C	-1.3941048	-2.2708825	-1.2442650
C	-1.5721428	-2.6347095	0.2374910
C	-3.1086208	-2.6937075	0.3548340
H	-3.2602668	-0.5907635	0.0049090
H	-0.5701028	-1.5498635	-1.3318210
H	-1.0492738	-3.5645165	0.5089060
H	-3.4449878	-3.6197345	-0.1589120
O	-1.0637458	-1.5503475	1.0162350
O	-3.6868508	-2.5719065	1.6108040
H	-2.9655098	-2.6900365	2.3083290
C	-1.1366458	-3.4407175	-2.1905970
H	-1.3021998	-3.0714675	-3.2171990
H	-1.8556138	-4.2529875	-1.9944210
C	-5.7797328	-0.5818405	-0.4637350
C	-5.5249758	-2.2970695	-1.8295360
C	-6.9925998	-0.9511805	-1.0526130
H	-4.9979078	-3.0783155	-2.3749400
C	-8.1794588	-0.2154055	-0.7081530
C	-6.6360688	1.1088295	0.7445230
H	-8.6762698	1.3606585	0.5250420
N	-4.8455828	-1.4475615	-0.9686950
N	-6.8044118	-2.0228055	-1.9060660
N	-5.5516598	0.4246795	0.4107400
N	-7.8719068	0.8112895	0.2319090
N	-6.5309768	2.1415665	1.6019970
H	-5.6428038	2.2938335	2.0644840
H	-7.3372398	2.6574675	1.9285340
O	-9.3312148	-0.3579265	-1.0905590
O	0.1594992	-3.9835055	-2.0564310
P	1.4610382	-2.9813385	-2.3610530
O	2.5578362	-4.0625185	-2.8017190
P	-0.7214288	-1.7220855	2.6333540
O	-0.7595898	-0.3096005	3.1920810
O	0.7876402	-2.2355565	2.6912050
O	-1.6954358	-2.7539155	3.2055210
O	4.3126512	-3.8329995	-0.9231790
O	1.8246202	-2.3397465	-1.0134120
P	3.7116342	-4.9505605	-1.8565120
O	4.7058532	-5.4154465	-2.9034240

P	1.5889512	-3.7363795	2.3649580	O	1.0967272	-2.0837145	-3.5151900
O	2.8135352	-3.2375115	1.5258420	O	2.9132662	-5.9987745	-1.1001420
O	2.0159452	-4.2305815	3.7375140	Fe	3.6600532	-2.1962355	0.0112490
O	0.5942192	-4.5965515	1.5913840				

$[Fe(ppGpp)_2]^{9-}$ (multiplicity = 2)

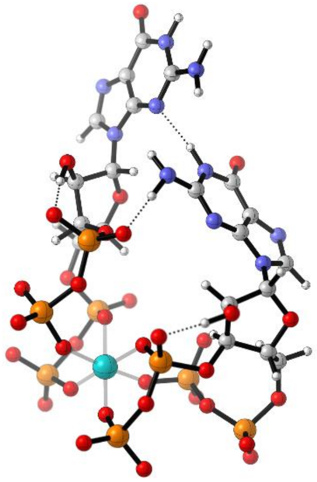

**E/hartree**	-6734.13
**E+zvp/hartree**	-6733.54
**G/hartree**	-6733.64

C	1.3945177	4.1821083	0.8780193
O	2.4963557	4.8189143	0.3062063
C	3.5851867	3.8839183	0.0526913
C	3.1118917	2.5451783	0.6515193
C	1.9197647	2.9323233	1.5651723
H	0.9241407	4.8595713	1.6081003
H	4.4826777	4.2398193	0.5805953
H	2.7874967	1.8816103	-0.1647527
H	1.1591617	2.1402833	1.5993583
O	4.1383847	1.9124263	1.3957223
O	2.3319117	3.2908303	2.8604463
H	2.6266097	2.4258153	3.2527413
C	3.8180227	3.9038333	-1.4697057
H	3.5282077	4.9144333	-1.8035727
H	3.1525487	3.1578683	-1.9341937
C	-0.7698513	3.1447913	0.0175593
C	0.3272397	4.3954813	-1.4383547
C	-1.4771553	3.2790783	-1.1861457
H	1.1320297	5.0187933	-1.8195737
C	-2.7730923	2.6618323	-1.2943337
C	-2.3052523	1.8594803	1.0183703
H	-3.9997683	1.4843253	-0.0899017
N	0.3920437	3.8636633	-0.1602137
N	-0.7681363	4.0612073	-2.0787017
N	-1.1371923	2.4677373	1.1237843
N	-3.0930103	1.9713383	-0.1056657
N	-2.7817353	1.1132483	2.0462943
H	-3.5856913	0.5279203	1.8312163
H	-2.0329243	0.6381583	2.6029783
O	-3.5670683	2.6749993	-2.2316167
O	5.1405917	3.7179613	-1.9302907
P	6.2062827	2.5113273	-1.4831527
O	5.2415387	1.2083533	-1.3017607
P	3.7626577	0.4664023	2.0906793
O	2.9861167	0.7798503	3.3667193
O	5.2181467	-0.0561227	2.4805013
O	3.0562557	-0.4048467	1.0527413
O	4.2083167	-1.0932207	-1.5950107
O	6.7752447	2.8596383	-0.1259047
P	4.1704487	0.3286103	-2.2817107
O	4.7829247	0.2393633	-3.6718537
P	6.2453377	-1.3128287	1.8865143
O	6.4512467	-2.2068457	3.0963573
O	5.4033197	-2.0152987	0.7541593
O	7.4427477	-0.5797477	1.3111613
O	7.1423597	2.3828943	-2.6621117
O	2.8179717	1.0420333	-2.1764627
C	-3.4891543	-1.5600447	-0.4743637
O	-2.6666743	-1.6503937	-1.6076107
C	-1.4210623	-2.2742087	-1.2159717
C	-1.5581123	-2.6468557	0.2671153
C	-3.0909853	-2.7394987	0.4187273
H	-3.3001253	-0.6409857	0.0953633
H	-0.6073663	-1.5446227	-1.3201307
H	-1.0133683	-3.5676487	0.5248423
H	-3.4201473	-3.6712167	-0.0890137
O	-1.0589213	-1.5529917	1.0356033
O	-3.6393223	-2.6337437	1.6897623
H	-2.8977733	-2.7380857	2.3675163
C	-1.1789453	-3.4362967	-2.1751697
H	-1.3635093	-3.0603127	-3.1960857
H	-1.8921963	-4.2519147	-1.9721577
C	-5.7979663	-0.6056957	-0.4269517
C	-5.5594353	-2.3900917	-1.7047097
C	-7.0093443	-0.9781077	-1.0168817
H	-5.0418583	-3.2074207	-2.2047687
C	-8.1831643	-0.1965137	-0.7349947
C	-6.6331903	1.1633013	0.6778493
H	-8.6613963	1.4549343	0.4024603
N	-4.8758313	-1.5154447	-0.8726827
N	-6.8313533	-2.0940677	-1.8141157
N	-5.5595323	0.4379683	0.3998823
N	-7.8663533	0.8701063	0.1562103
N	-6.5194303	2.2352253	1.4844223
H	-5.6371223	2.3902133	1.9568143
H	-7.3196063	2.7835533	1.7706853
O	-9.3305903	-0.3292187	-1.1335447
O	0.1204137	-3.9757337	-2.0666127
P	1.4113937	-2.9603687	-2.3741947
O	2.5107777	-4.0250277	-2.8461477
P	-0.6622463	-1.7237507	2.6407783
O	-0.7209213	-0.3125407	3.2022613
O	0.8575067	-2.2040947	2.6497703
O	-1.5985103	-2.7789557	3.2333223
O	4.2875837	-3.7774467	-0.9729907
O	1.7732517	-2.3371197	-1.0144837
P	3.6637967	-4.8994487	-1.8930557
O	4.6673377	-5.3714157	-2.9287767
P	1.7030677	-3.6941957	2.3802833
O	2.9532397	-3.2152677	1.5574553
O	2.1220097	-4.1343087	3.7745583
O	0.7299547	-4.5995117	1.6311853

O	1.0235157	-2.0492017	-3.5091517	Fe	3.6503487	-2.1635277	-0.0385067
O	2.8624947	-5.9465157	-1.1384147				

$[Fe(pppGpp)_2]^{11-}$ (multiplicity = 6)

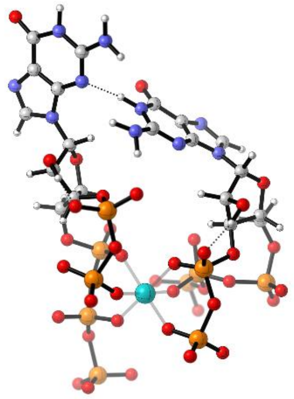

**E/hartree**	-7868.17
**E+zvp/hartree**	-7867.55
**G/hartree**	-7867.66

C	-0.0550396	4.5491350	-0.4975439
O	-1.1236736	5.2462050	0.0756371
C	-2.3910206	4.5712720	-0.1324609
C	-2.0217546	3.2385360	-0.7999119
C	-0.6423346	3.5114980	-1.4471669
H	0.5877154	5.2504080	-1.0541679
H	-3.0057456	5.1673740	-0.8257379
H	-1.9462436	2.4537530	-0.0311179
H	-0.0107976	2.6121050	-1.4867399
O	-2.9944536	2.8865590	-1.7564429
O	-0.7671866	4.0971150	-2.7187879
H	-1.1319306	3.3620390	-3.2784549
C	-3.0789426	4.4824730	1.2384781
H	-2.7159406	5.3458120	1.8200901
H	-2.7673266	3.5518590	1.7402941
C	1.8347734	3.0947120	0.3848571
C	0.7354484	4.2500450	1.9161901
C	2.4072964	2.9430650	1.6557581
H	-0.0304966	4.9109680	2.3157251
C	3.5920654	2.1338340	1.7799441
C	3.2827684	1.7497020	-0.6632119
H	4.7798844	0.9740690	0.5210281
N	0.7585914	3.9343000	0.5667721
N	1.6989264	3.6715370	2.5937831
N	2.2336744	2.5438830	-0.7780279
N	3.9495924	1.5840550	0.5293401
N	3.7557764	1.0828020	-1.7460919
H	4.4249494	0.3410030	-1.5544259
H	3.0225244	0.8333570	-2.4466679
O	4.2720524	1.8925670	2.7739121
O	-4.4901596	4.5834680	1.2446431
P	-5.5872916	3.6674160	0.3864011
O	-4.8587806	2.1896530	0.3291471
P	-2.7800976	1.5132420	-2.6236089
O	-1.7605926	1.8302380	-3.7096379
O	-4.2293426	1.3469980	-3.2603749
O	-2.4236856	0.3879360	-1.6432059
O	-4.3510256	-0.2732180	0.4877941
O	-5.6589486	4.1771770	-1.0311789
P	-4.3167136	1.0099660	1.3486441
O	-5.4968676	0.9530230	2.4073561
P	-5.4595496	0.1224090	-3.2419219
O	-5.4815556	-0.3897660	-4.6684299
O	-4.8889946	-0.9555310	-2.2405509
O	-6.6757526	0.8370900	-2.6908819
O	-6.8269966	3.6460070	1.2430471
O	-2.9717886	1.4150400	1.9169401
C	3.6742754	-1.8853750	0.5185841
O	2.6812314	-1.9450650	1.5083271
C	1.4271134	-2.2739390	0.8674501
C	1.7321934	-2.5377510	-0.6150199
C	3.2361734	-2.8770970	-0.5621979
H	3.7257644	-0.8937250	0.0523511
H	0.7563714	-1.4061780	0.9363901
H	1.0935904	-3.3274550	-1.0402139
H	3.3264354	-3.8978640	-0.1326959
O	1.5179084	-1.3155020	-1.3259679
O	3.9938424	-2.7447610	-1.7176319
H	3.3680414	-2.6530310	-2.5051189
C	0.8291064	-3.4507310	1.6320561
H	0.8882034	-3.2162730	2.7078761
H	1.4166854	-4.3625290	1.4402191
C	6.1036444	-1.4033620	0.8622471
C	5.3517774	-3.1926320	1.9154121
C	7.1275284	-2.0611390	1.5504651
H	4.6217534	-3.9209710	2.2652601
C	8.4593624	-1.5230370	1.4868851
C	7.4125104	0.2295770	0.0447231
H	9.4016524	0.0734850	0.5864711
N	4.9704184	-2.1350210	1.1026331
N	6.6298514	-3.1730510	2.2041021
N	6.1896584	-0.2748180	0.1204451
N	8.4819094	-0.3519510	0.6749991
N	7.6237264	1.3623000	-0.6536799
H	6.8659564	1.7210520	-1.2218469
H	8.5541354	1.7222280	-0.8223579
O	9.4934524	-1.9214570	2.0016201
O	-0.5044796	-3.7262610	1.2530981
P	-1.6679076	-2.5885550	1.5837231
O	-2.9537956	-3.5604180	1.7508871
P	1.3717114	-1.2736620	-2.9794899
O	1.6672174	0.1651620	-3.3589639
O	-0.1755686	-1.5560690	-3.2801029
O	2.2554114	-2.3802390	-3.5556089
O	-4.3380246	-2.9983990	-0.3489009
O	-1.8375596	-1.7272550	0.3282121
P	-3.9478646	-4.2028720	0.5403231
O	-5.1523076	-4.6268620	1.4664981
P	-1.1813056	-2.9524480	-3.1667599
O	-2.4266186	-2.3720810	-2.4031589
O	-1.5196026	-3.2874010	-4.6074999
O	-0.4082076	-3.9821980	-2.3527079
O	-1.3229026	-1.9178340	2.8810741
O	-3.2127626	-5.3323680	-0.1297489
Fe	-3.4058586	-1.3447560	-1.0303429
P	-5.5458886	0.2872000	4.0753621

P	-6.8701176	-4.9132700	0.9100401
O	-7.5119306	-3.5374180	1.1013021
O	-7.2841446	-5.9906820	1.9115021
O	-6.6746736	-5.3759380	-0.5372149
O	-7.0608426	0.1964330	4.2769781
O	-4.8099156	-1.0504760	3.9351461
O	-4.8313416	1.3575550	4.9055371

[Fe(pppGpp)$_2$]$^{11-}$ (multiplicity = 2)

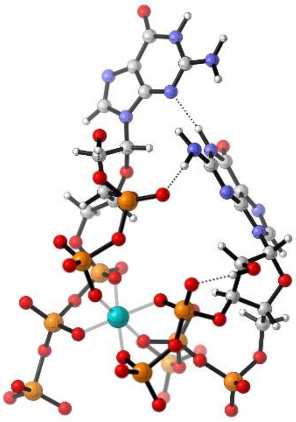

**E/hartree**	-7868.11
**E+zvp/hartree**	-7867.49
**G/hartree**	-7867.60

C	0.0994069	4.4995654	0.8050107
O	1.1812959	5.2306164	0.3034957
C	2.4381019	4.5168274	0.4475567
C	2.0459369	3.1438434	1.0115167
C	0.6675419	3.3854604	1.6737597
H	-0.5444811	5.1620044	1.4059767
H	3.0706749	5.0474014	1.1764877
H	1.9671019	2.4217964	0.1848537
H	0.0289779	2.4913054	1.6468217
O	3.0042599	2.6988244	1.9457467
O	0.7935579	3.8659144	2.9888237
H	1.1203989	3.0706714	3.4835387
C	3.1034849	4.5197554	-0.9368883
H	2.7808479	5.4526164	-1.4286093
H	2.7319169	3.6559614	-1.5105933
C	-1.7835661	3.1212284	-0.2109643
C	-0.6676621	4.3962454	-1.6319323
C	-2.3398681	3.0717774	-1.4974503
H	0.1019539	5.0875344	-1.9678903
C	-3.5219171	2.2757514	-1.7034823
C	-3.2590301	1.7136334	0.7111977
H	-4.7334591	1.0325774	-0.5512903
N	-0.7058191	3.9732834	-0.3127693
N	-1.6218031	3.8733494	-2.3654913
N	-2.1985321	2.4794214	0.8994147
N	-3.9004551	1.6338994	-0.5048573
N	-3.7760511	0.9922044	1.7364127
H	-4.4613551	0.2862944	1.4753457
H	-3.0686741	0.6746924	2.4387507
O	-4.1840421	2.1118274	-2.7247763
O	4.5176349	4.5386064	-0.9695813
P	5.5920799	3.5408834	-0.1758633
O	4.8562989	2.0666314	-0.2386773
P	2.7564609	1.2548564	2.6727697
O	1.6650349	1.4588364	3.7115127
O	4.1660149	1.0368194	3.3768697
O	2.4821059	0.2283654	1.5586407
O	4.3297809	-0.3614546	-0.7931053
O	5.6628509	3.9461944	1.2757667
P	4.2431499	1.0513634	-1.4018283
O	5.3470019	1.2044304	-2.5386963
P	5.3986689	-0.1700646	3.2225457
O	5.3364539	-0.9364446	4.5288087
O	4.9279759	-1.0333796	1.9779827
O	6.6450189	0.6225034	2.8877847
O	6.8406859	3.5757844	-1.0194223
O	2.8691019	1.5522964	-1.8128983
C	-3.6652001	-1.8621546	-0.6544093

O	-2.6754301	-1.8251646	-1.6505413
C	-1.4157891	-2.1953116	-1.0411823
C	-1.7169081	-2.5627806	0.4189807
C	-3.2101691	-2.9390266	0.3340967
H	-3.7190451	-0.9161396	-0.1019323
H	-0.7476391	-1.3229776	-1.0524993
H	-1.0588941	-3.3574326	0.8024707
H	-3.2768961	-3.9199326	-0.1830873
O	-1.5560721	-1.3790316	1.2040887
O	-3.9689161	-2.9276076	1.4959137
H	-3.3439391	-2.8758236	2.2871107
C	-0.8216001	-3.3140316	-1.8925563
H	-0.8538591	-2.9856776	-2.9448223
H	-1.4312471	-4.2259976	-1.7937053
C	-6.0890801	-1.3439476	-0.9264613
C	-5.3790381	-3.0729896	-2.1017313
C	-7.1365071	-1.9548296	-1.6215283
H	-4.6653931	-3.7857276	-2.5124013
C	-8.4635631	-1.4192306	-1.4798083
C	-7.3622261	0.2417484	0.0293097
H	-9.3699891	0.1176894	-0.4477213
N	-4.9674041	-2.0658716	-1.2406393
N	-6.6644911	-3.0294726	-2.3527163
N	-6.1447821	-0.2604626	-0.1181873
N	-8.4543501	-0.2988716	-0.5982673
N	-7.5441761	1.3271554	0.8052077
H	-6.7613711	1.6626714	1.3533567
H	-8.4629351	1.7008024	1.0041467
O	-9.5165881	-1.7849916	-1.9799633
O	0.4977819	-3.6558896	-1.5145963
P	1.6882879	-2.5108726	-1.6685483
O	2.9692549	-3.4752286	-1.8842283
P	-1.4111261	-1.4488296	2.8581627
O	-1.7961771	-0.0621046	3.3412447
O	0.1522269	-1.6552466	3.1263027
O	-2.2264601	-2.6457066	3.3477957
O	4.2624329	-3.0579396	0.3180797
O	1.7960329	-1.8089986	-0.3018983
P	3.9279709	-4.1909106	-0.6924373
O	5.1782649	-4.5168926	-1.5933563
P	1.1843119	-3.0352536	3.0768317
O	2.5125419	-2.4054326	2.5029197
O	1.3794349	-3.4186416	4.5330897
O	0.5107009	-4.0460796	2.1604267
O	1.4076239	-1.6930676	-2.8929613
O	3.1953929	-5.3873436	-0.1496863
Fe	3.3625809	-1.4550906	0.9784157
P	5.3496379	0.7786284	-4.2768793

P	6.8878139	-4.7895266	-1.0104563
O	7.4909169	-3.3884256	-1.1293783
O	7.3546579	-5.8100236	-2.0471443
O	6.6700059	-5.3187636	0.4104067
O	6.8566969	0.6282084	-4.5094243
O	4.5337619	-0.5186826	-4.3339073
O	4.7038679	2.0010154	-4.9396193

salene^{2-}

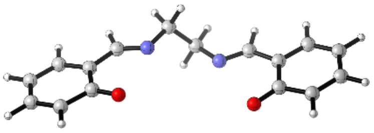

**E/hartree**	-877.72
**E+zvp/hartree**	-877.46
**G/hartree**	-877.50

C	-6.1936237	-1.4862705	-0.2121100
C	-5.0011627	-2.1359505	0.0437490
C	-3.7317057	-1.4487285	0.2270240
C	-3.8161297	0.0135345	0.1137900
C	-5.0622297	0.6305715	-0.1455960
C	-6.2459817	-0.0773575	-0.3117480
H	-7.1111367	-2.0720365	-0.3389040
H	-4.9649387	-3.2277495	0.1213790
H	-5.0798847	1.7247125	-0.2186360
H	-7.1878797	0.4383185	-0.5127030
O	-2.6757857	-2.0898605	0.4628260
C	-2.6803387	0.9127415	0.2563580
H	-2.9728397	1.9862425	0.1612620
N	-1.4448807	0.6341335	0.4702410
C	-0.5339047	1.7509085	0.5545500
H	-1.0643757	2.7294645	0.5080080
H	-0.0011767	1.7173465	1.5243980
C	0.5338383	1.7508745	-0.5542920
H	1.0643123	2.7294335	-0.5078200
H	0.0011013	1.7172445	-1.5241320
C	2.6802883	0.9127055	-0.2561880
H	2.9728243	1.9862215	-0.1613610
N	1.4448043	0.6340945	-0.4699140
C	3.8161083	0.0135145	-0.1137120
C	5.0622463	0.6305965	0.1453820
C	3.7316863	-1.4487635	-0.2267730
C	6.2460383	-0.0772965	0.3114020
H	5.0798973	1.7247465	0.2182970
C	5.0011883	-2.1359435	-0.0436650
C	6.1936863	-1.4862185	0.2119010
H	7.1879723	0.4384155	0.5120970
H	4.9649753	-3.2277505	-0.1211930
H	7.1112333	-2.0719575	0.3385800
O	2.6757753	-2.0899375	-0.4624980

water

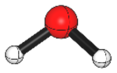

**E/hartree**	-76.37
**E+zvp/hartree**	-76.34
**G/hartree**	-76.36

O	0.0000000	0.0000000	0.4036067
H	0.0000000	0.7551370	-0.2018033
H	0.0000000	-0.7551370	-0.2018033

ppGpp^{6-}

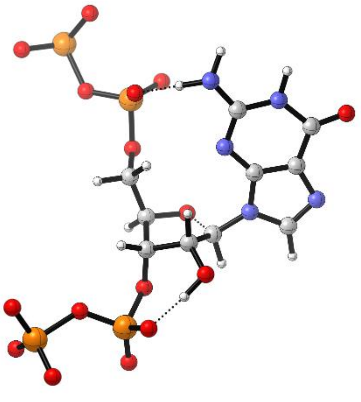

**E/hartree**	-3305.14
**E+zvp/hartree**	-3304.85
**G/hartree**	-3304.92

C	0.8255824	1.6777101	-1.0206994
O	0.2641294	0.5222131	-1.5960694
C	0.8646014	-0.6581649	-1.0201914
C	2.0673424	-0.1528769	-0.2178064

C	1.5563534	1.2226171	0.2545686
H	1.5735724	2.1406911	-1.6891354
H	1.2177764	-1.2892089	-1.8513464
H	2.3172134	-0.8255219	0.6186686
H	0.7854944	1.0437261	1.0278946
O	3.1729844	-0.0085199	-1.0897424
O	2.4944624	2.1501471	0.6899246
H	3.4029714	1.7022001	0.7265076
C	-0.1342166	-1.4343039	-0.1742314
H	-0.4604846	-0.8267849	0.6810886
H	0.3877244	-2.3304339	0.2217136
C	-1.3666686	2.5599931	-0.0843784
C	-0.1820356	3.9731691	-1.3098194
C	-1.9931726	3.8117571	-0.1956974
H	0.6489014	4.3174791	-1.9262084
C	-3.2652116	4.0022671	0.4439826
C	-2.9856576	1.6121641	1.1442906
H	-4.5792246	2.8793291	1.5516416
N	-0.1989086	2.6802321	-0.8088844
N	-1.2296906	4.6758471	-0.9618094
N	-1.7990596	1.4701751	0.5682206
N	-3.6742096	2.8125341	1.0931416

N	-3.5353206	0.5873941	1.8115576
H	-3.1564516	-0.3913689	1.6025566
H	-4.5002326	0.6716091	2.1089966
O	-3.9827866	4.9978701	0.4814496
O	-1.2411626	-1.7997719	-0.9612704
P	-2.7045926	-2.2189949	-0.2507404
O	-2.6074046	-3.8100829	-0.4017514
P	4.7306664	-0.0951899	-0.4456684
O	5.6452684	0.1582931	-1.6161364
O	4.7015854	-1.6010669	0.0861066
O	4.7294554	0.8952411	0.7312556
O	-4.9941456	-4.2719179	0.5854336
O	-3.7695486	-1.5746199	-1.1056004
P	-3.9466756	-4.9971859	-0.2715614
O	-4.3332446	-5.2302029	-1.7371844
P	6.0906824	-2.6276909	0.5951386
O	7.1535374	-1.5974599	0.9989416
O	5.4486404	-3.4115839	1.7424676
O	6.3910754	-3.4296959	-0.6751244
O	-2.6072946	-1.8208249	1.2319216
O	-3.2226206	-6.1611879	0.4135886

pppGpp^{7-}

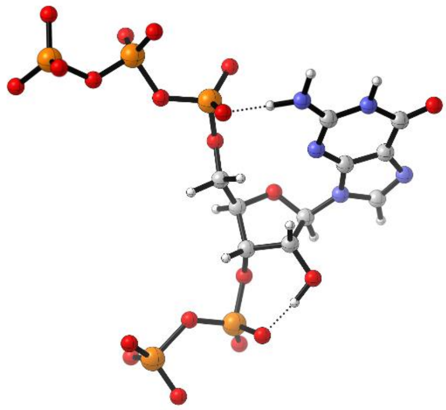

**E/hartree**	-3872.15
**E+zvp/hartree**	-3871.84
**G/hartree**	-3871.92

C	2.3834536	1.0723850	-0.9346765
O	1.2497956	0.5862720	-1.6142355
C	0.8905706	-0.7151680	-1.1047085
C	2.0659076	-1.1489970	-0.2230395
C	2.5421346	0.2101890	0.3289565
H	3.2994226	0.9508650	-1.5401825
H	0.8036516	-1.3967580	-1.9656595
H	1.7475956	-1.8416050	0.5729195
H	1.7878316	0.5559240	1.0615945
O	3.0627096	-1.7404230	-1.0333455
O	3.8256656	0.2800630	0.8549275
H	4.1828676	-0.6645720	0.9468735
C	-0.4255834	-0.6738620	-0.3424225
H	-0.3300764	-0.0400300	0.5496175
H	-0.6553734	-1.7059120	-0.0099805
C	1.2540376	3.1517220	0.0008285
C	3.1639836	3.4656280	-1.0737395
C	1.6299406	4.5042170	-0.0183005
H	4.0558196	3.1941680	-1.6393185
C	0.7709996	5.4662380	0.6148445
C	-0.6611564	3.4750010	1.1204545
H	-1.0265334	5.4584800	1.6094795
N	2.2539376	2.4939320	-0.6851975
N	2.8274406	4.6711090	-0.6919065

N	0.1594446	2.6028870	0.5518415
N	-0.3696304	4.8269170	1.1583635
N	-1.7970924	3.0518120	1.6953035
H	-2.1382404	2.0815240	1.4398185
H	-2.4791964	3.7399830	1.9899815
O	0.9021856	6.6817030	0.7237725
O	-1.4488874	-0.1771350	-1.1775905
P	-2.8693084	0.3976440	-0.5287055
O	-3.7776584	-0.9390420	-0.7304705
P	4.1264096	-2.8417490	-0.3190925
O	5.1488996	-3.1368930	-1.3857695
O	3.0991886	-4.0255920	-0.0155405
O	4.5802176	-2.1615790	0.9846115
O	-5.9889744	0.0674590	0.1986175
O	-3.3125714	1.5358740	-1.4085855
P	-5.4901434	-0.9945430	-0.7617215
O	-5.8877924	-0.9253460	-2.2211015
P	3.4723136	-5.7294480	0.4447295
O	4.8635196	-5.6188490	1.0820685
O	2.3154806	-6.0126940	1.4062395
O	3.4162576	-6.4562630	-0.9025995
O	-2.6448444	0.6224860	0.9676185
O	-5.5970874	-2.4884220	-0.1737365
P	-7.0441434	-3.3038830	0.4606065

O	-6.7830034	-4.7458020	0.0051735
O	-6.9413294	-3.0659530	1.9734565
O	-8.2130574	-2.5939600	-0.2410715

6. NMR Spektra

Compound **8** (ppAp), ¹H-NMR (D_2O, 400 MHz)

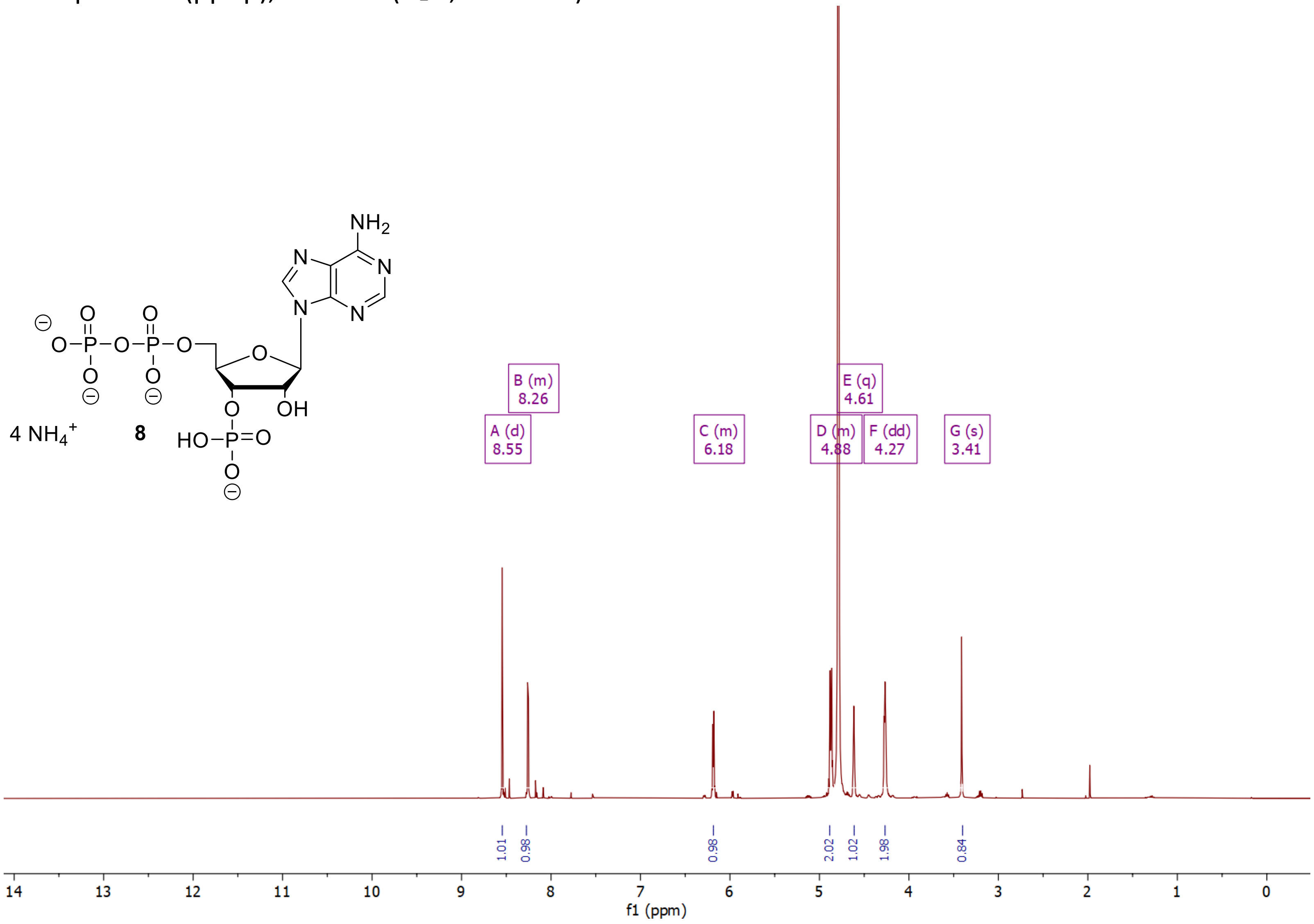

Compound **8** (ppAp), $^{31}P[^{1}H]$-NMR (D_2O, 162 MHz)

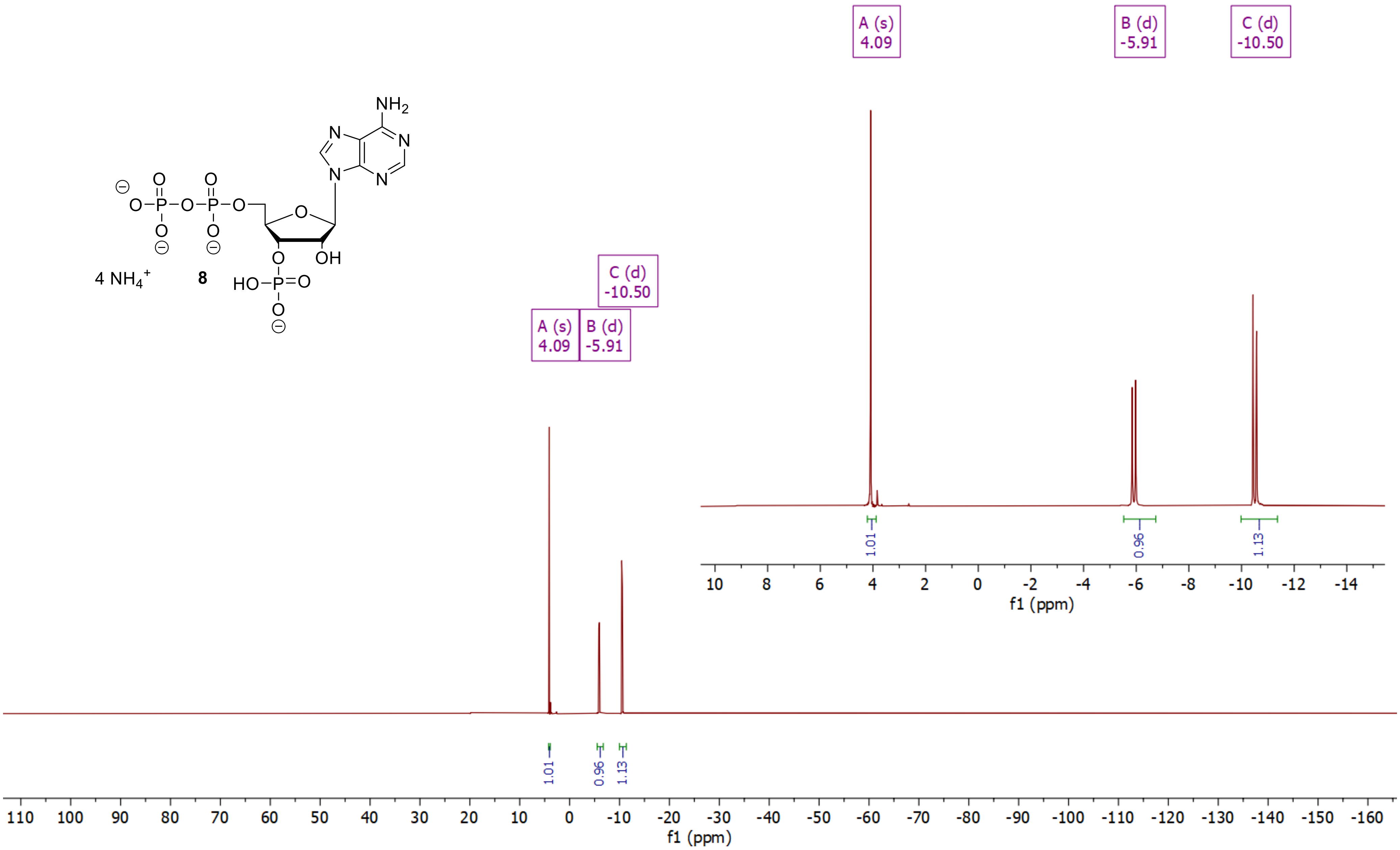

Compound **8** (ppAp), $^{13}C[^{1}H]$-NMR (D_2O, 101 MHz)

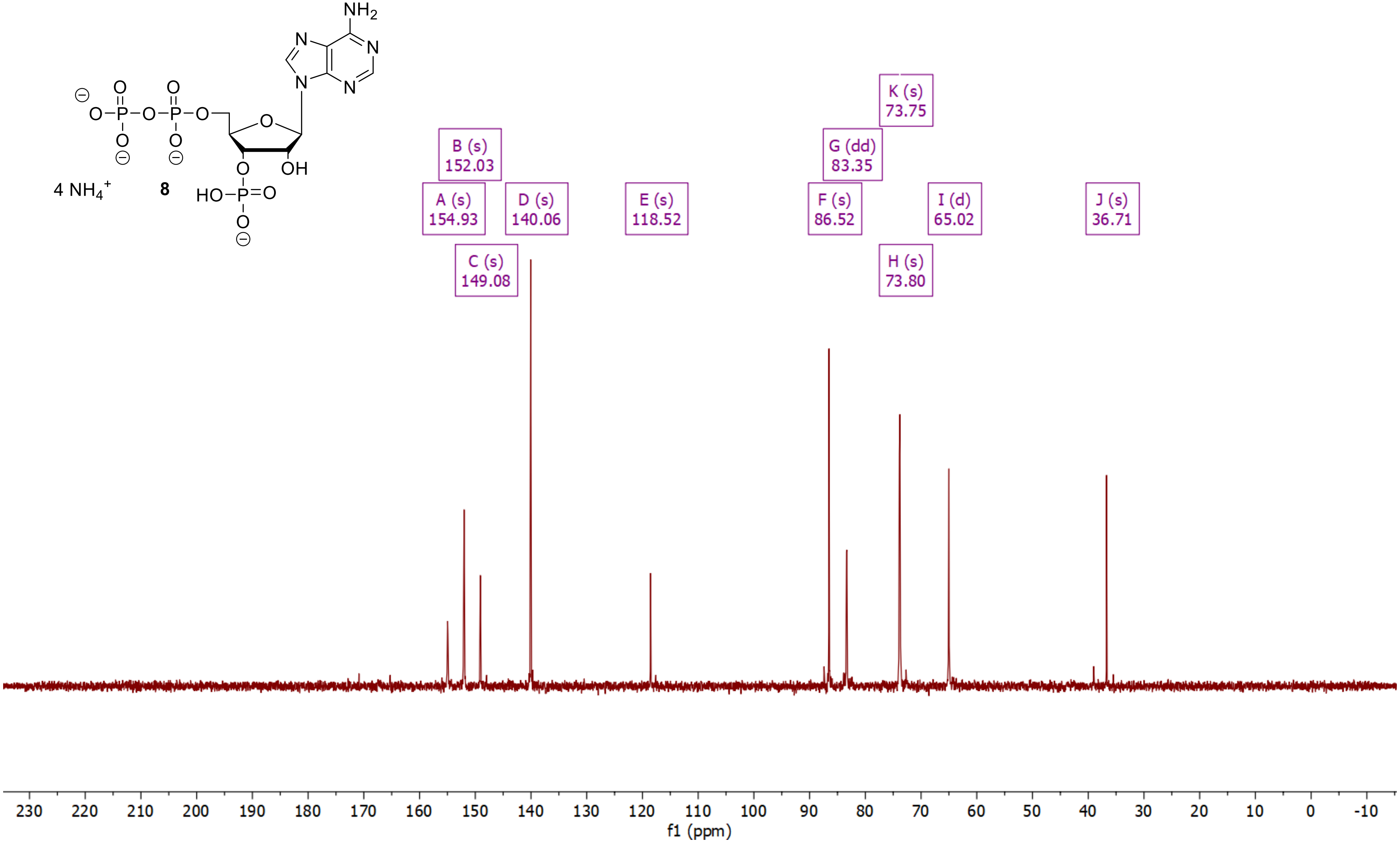

Compound **8** (ppApp), $^{13}C[^{1}H]$-NMR (D_2O, 101 MHz), detailed

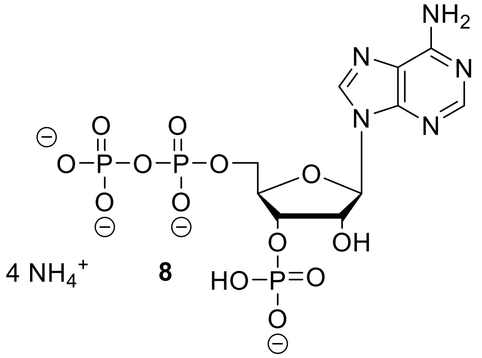

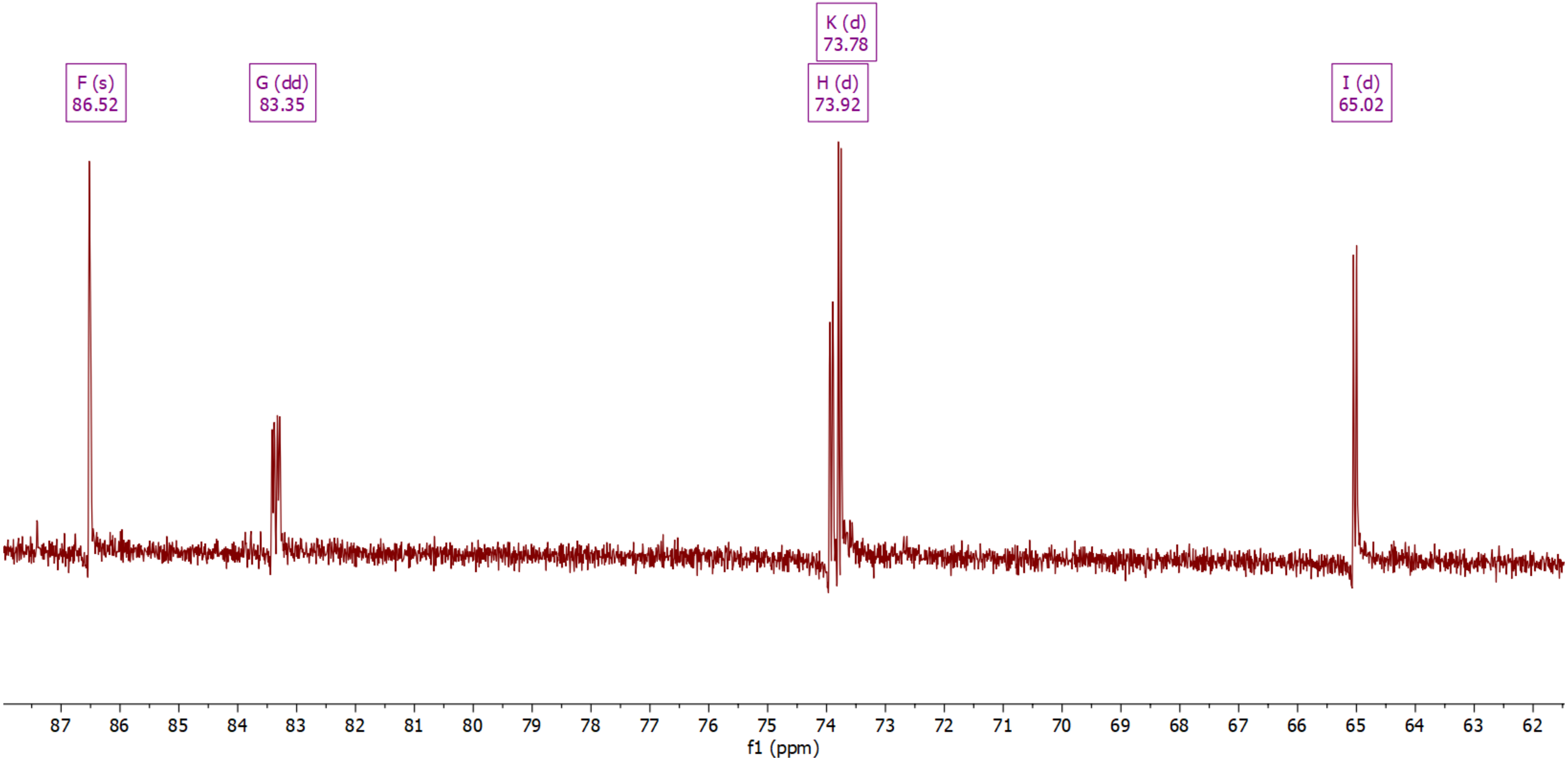

Compound **11** (ppGp), ^{1}H-NMR (D_2O, 400 MHz)

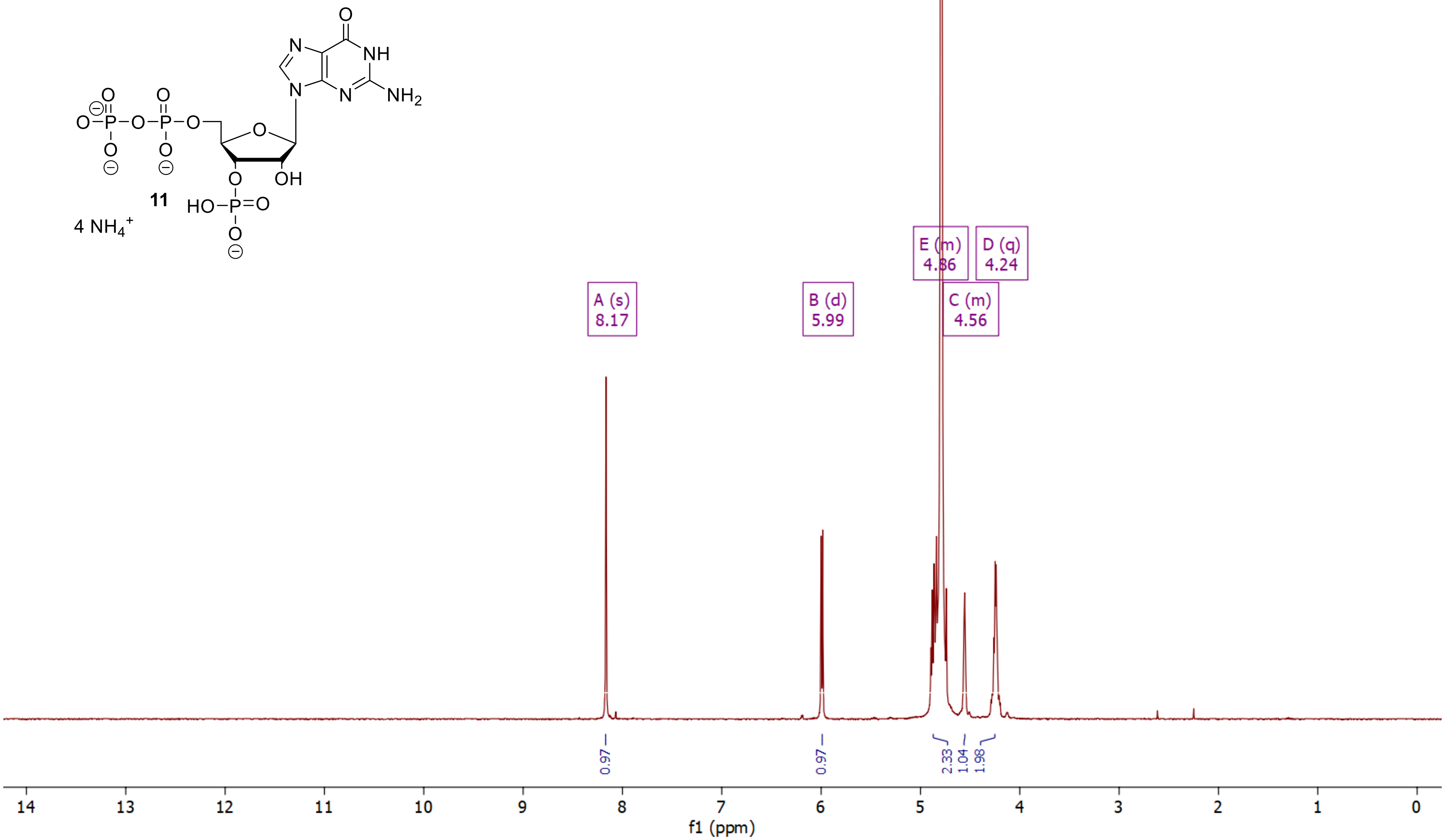

Compound **11** (ppGp), ed-HSQC (D_2O)

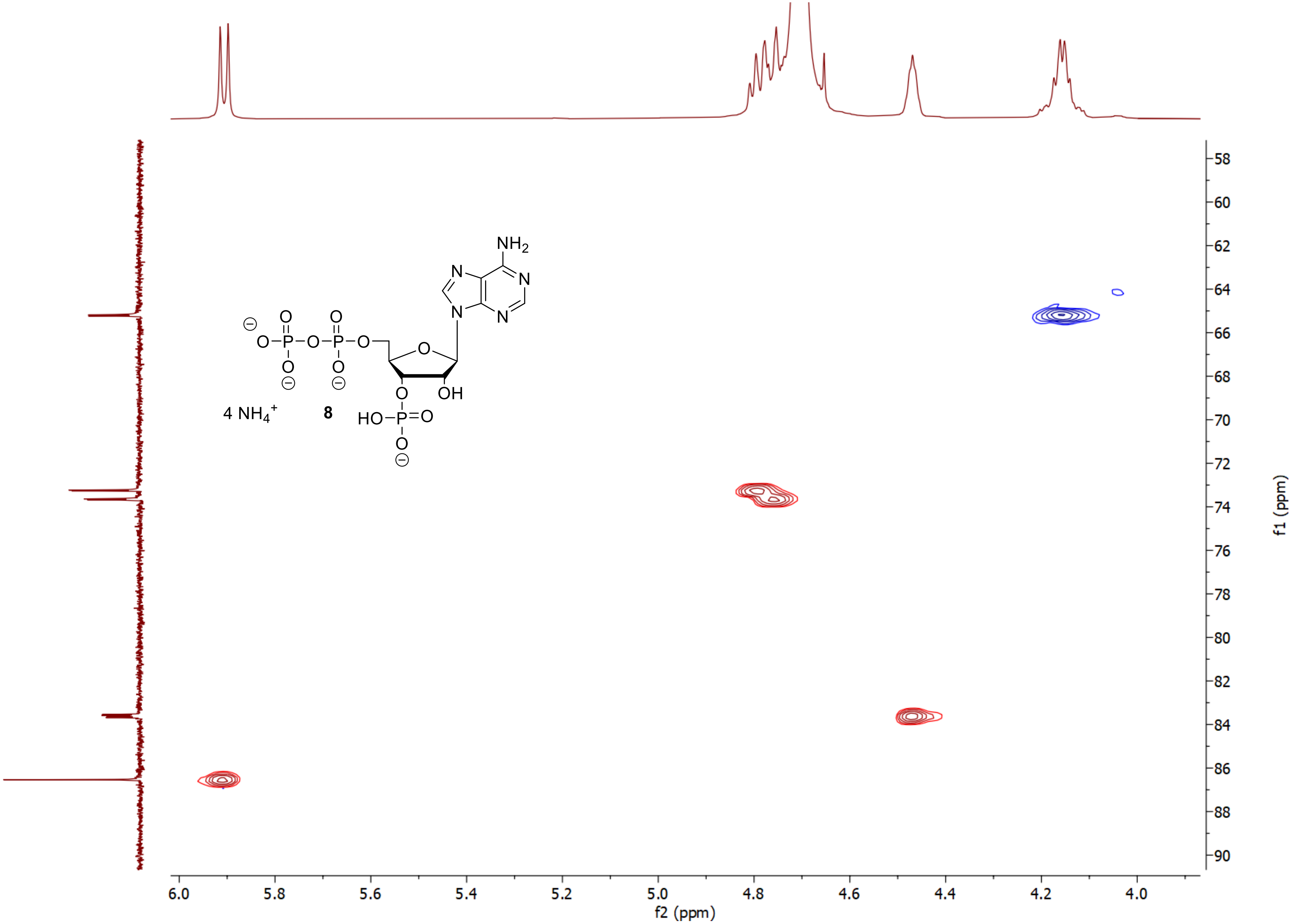

Compound **11** (ppGp), $^{31}P[^{1}H]$ - NMR (D_2O, 162 MHz)

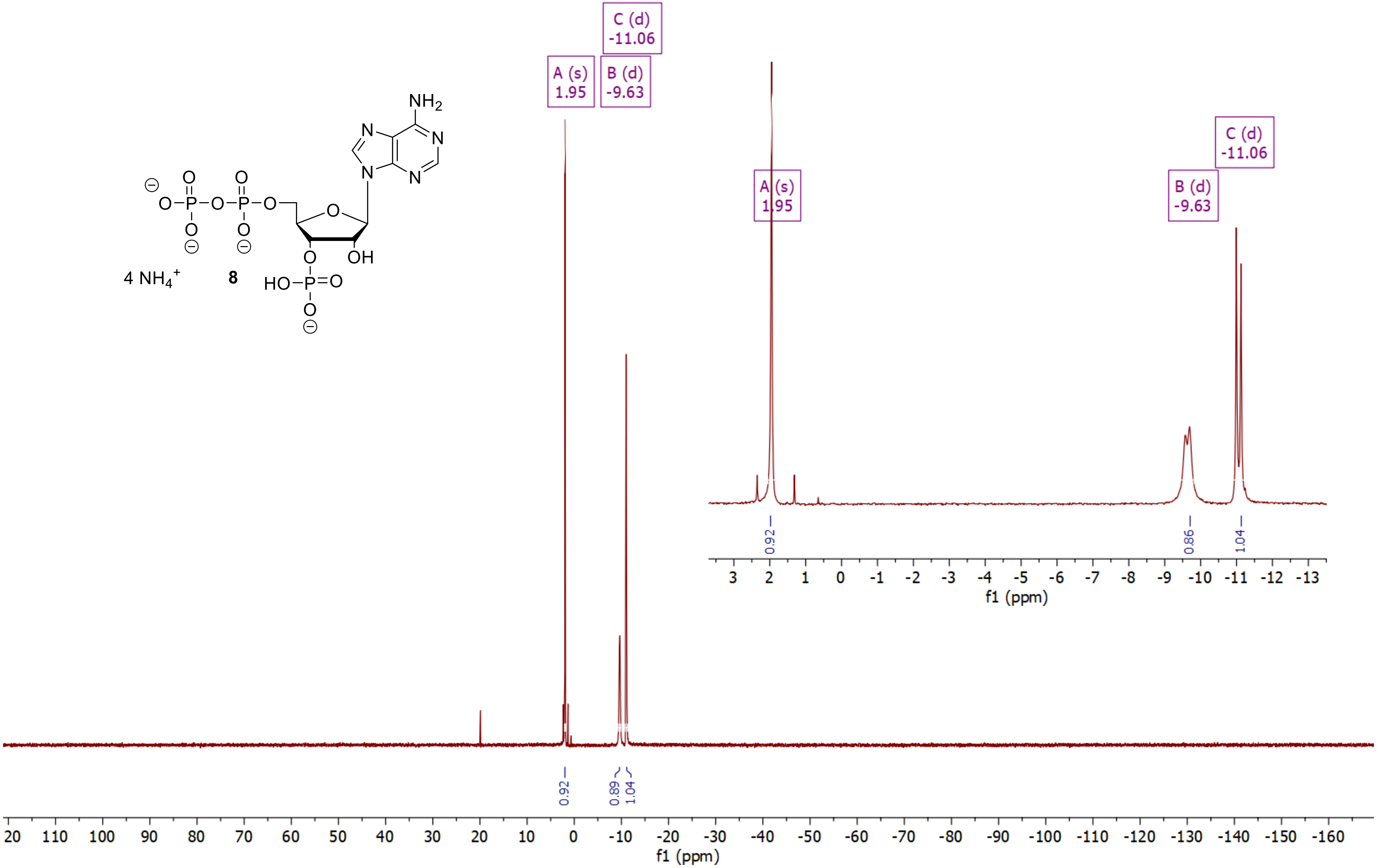

Compound **11** (ppGp), $^{13}C[^{1}H]$ - NMR (D_2O, 101 MHz)

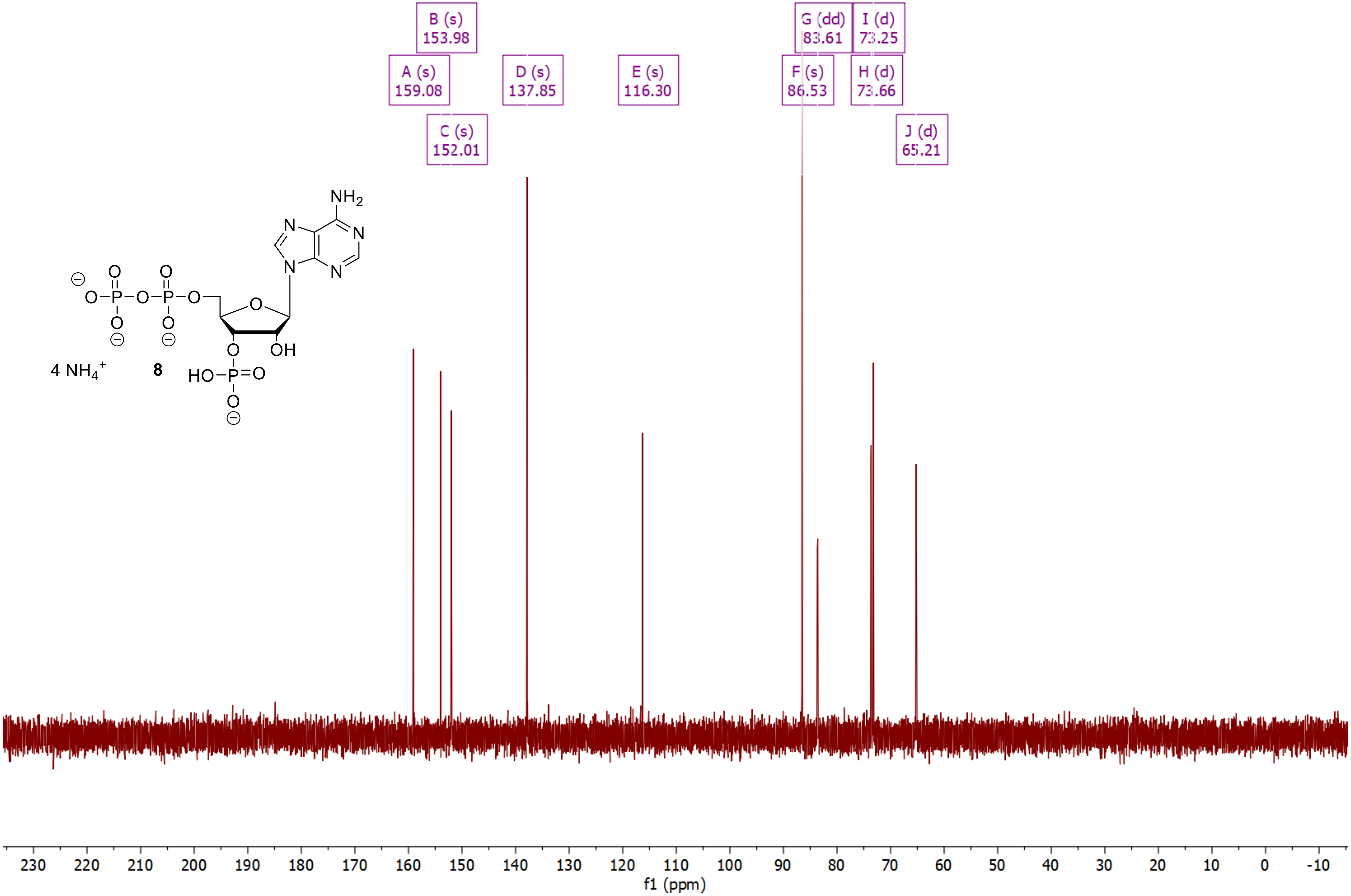

Compound **11** (ppGp), $^{13}C[^{1}H]$-NMR (D_2O, 101 MHz), detailed

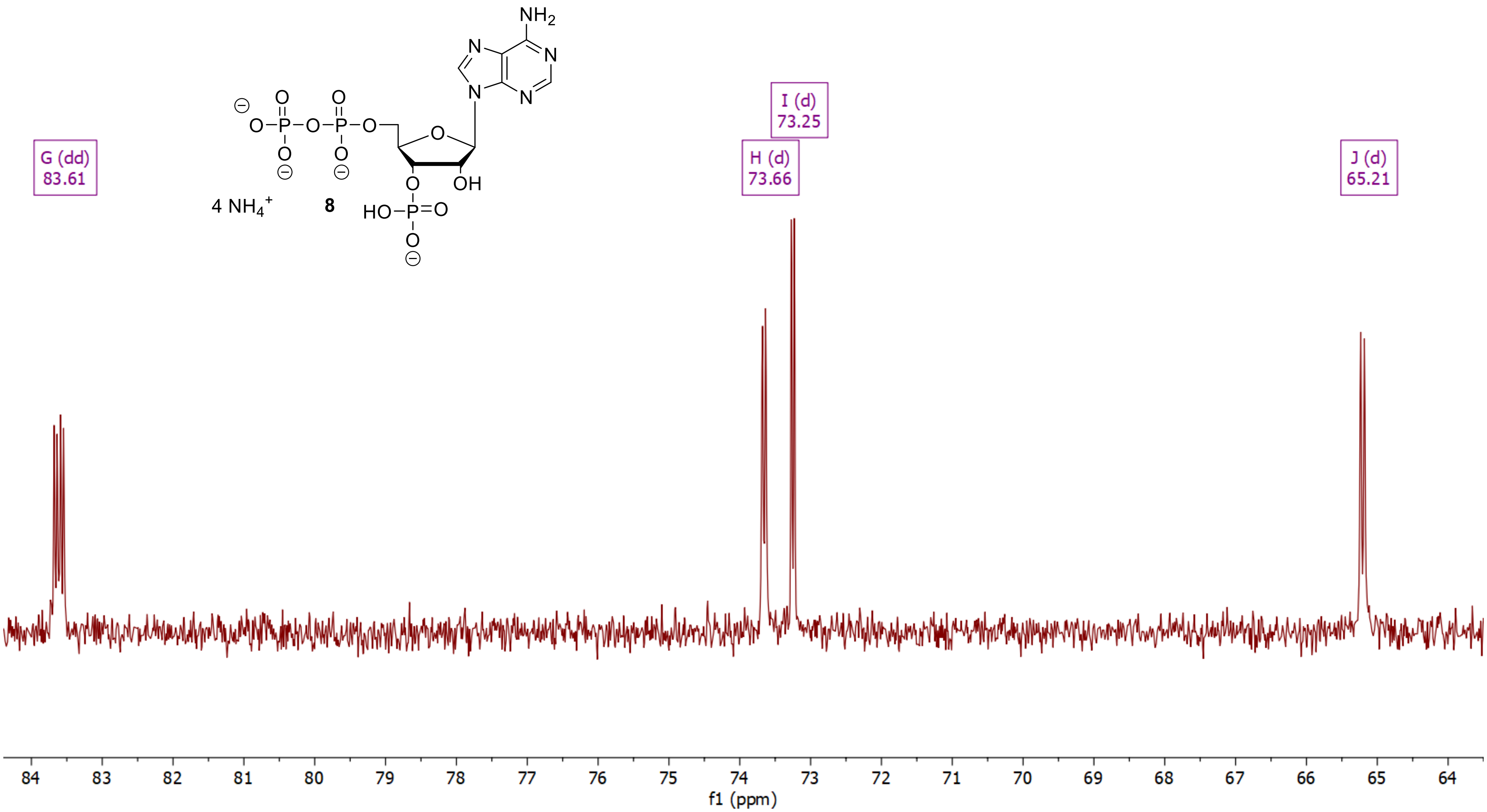

Compound **13** (pppApp), ^{1}H-NMR (D_2O, 400 MHz)

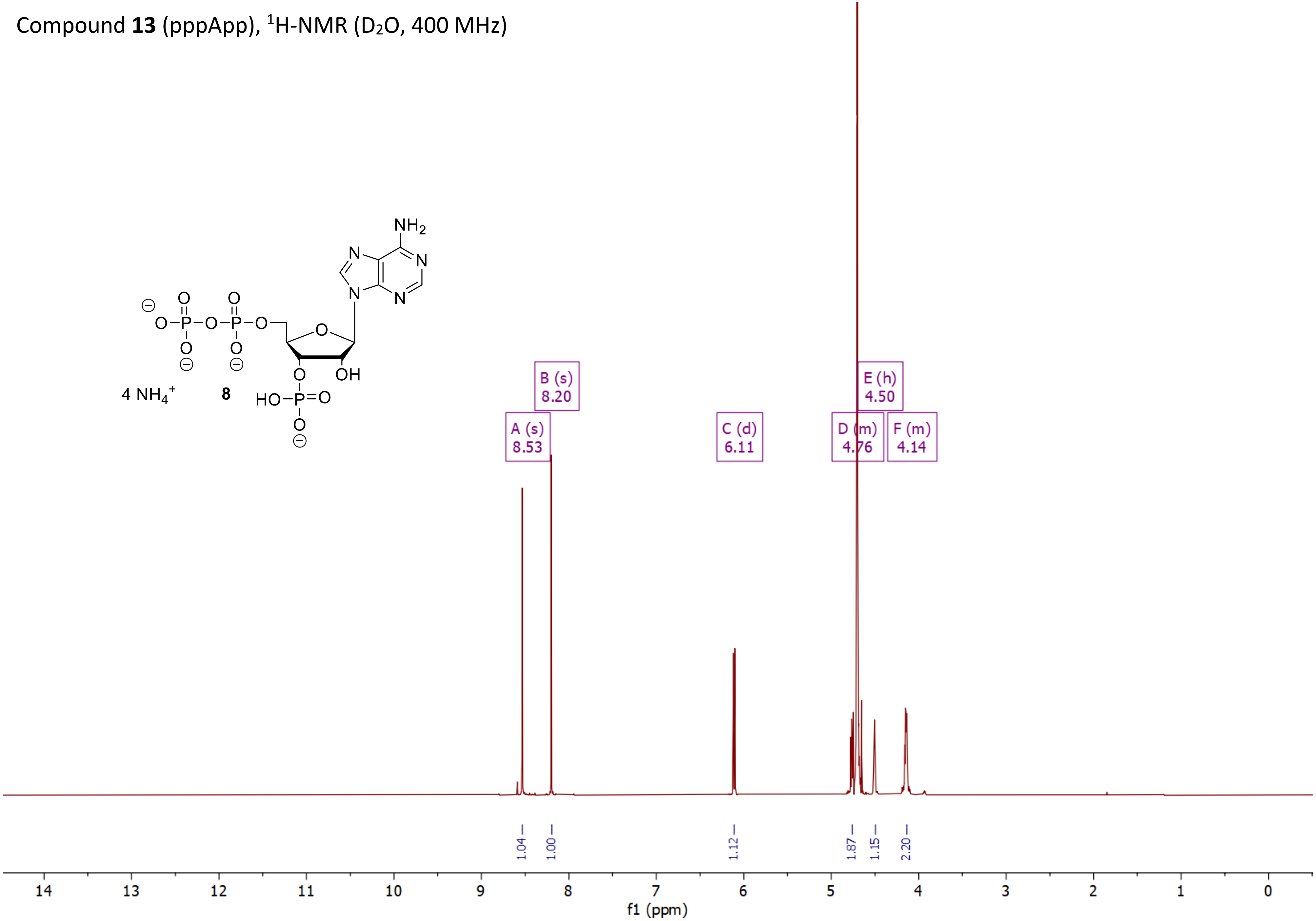

Compound **13** (pppApp), $^{31}P[^{1}H]$-NMR (D_2O, 162 MHz)

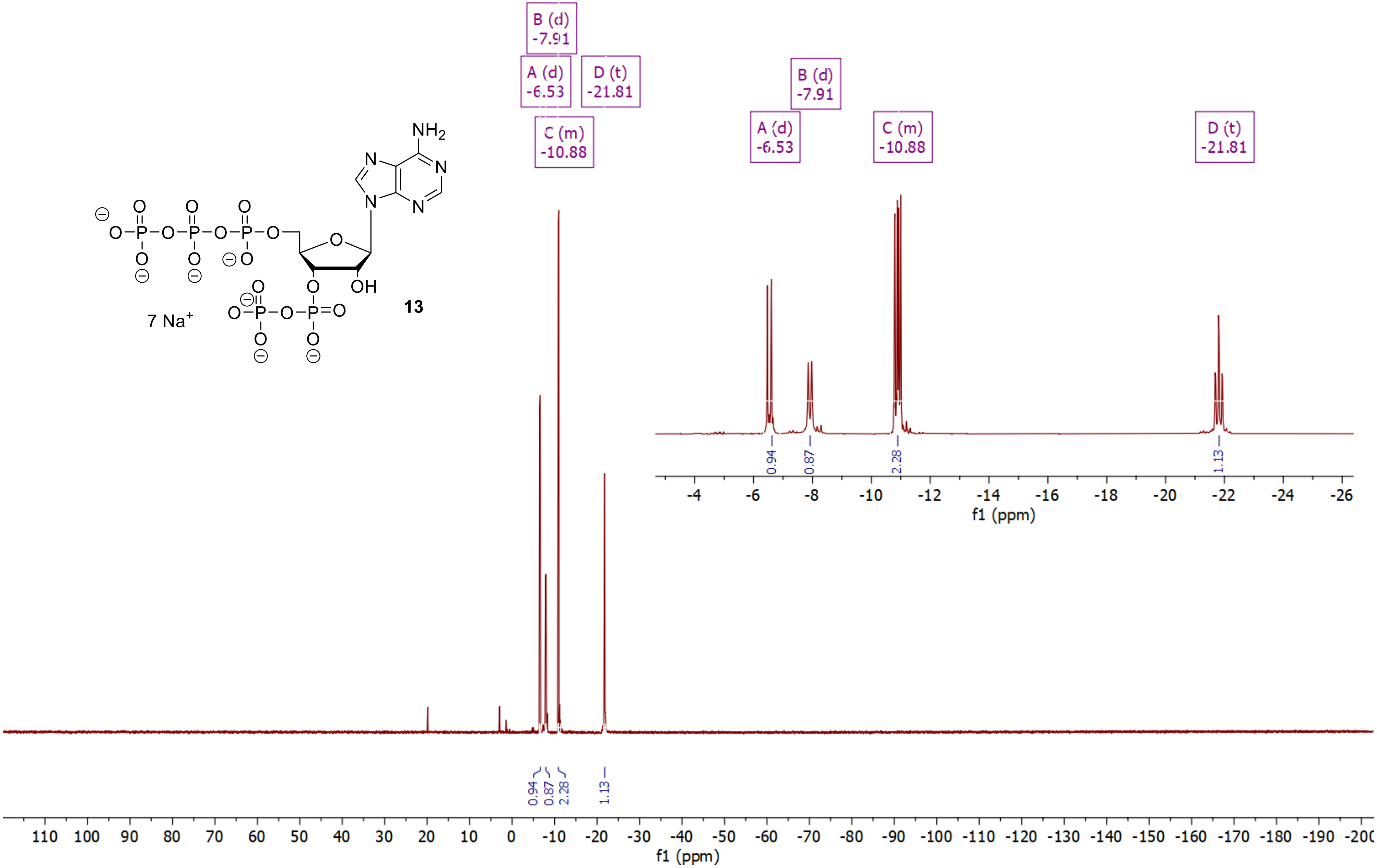

Compound **13** (pppApp), $^{13}C[^{1}H]$-NMR (D_2O, 101 MHz)

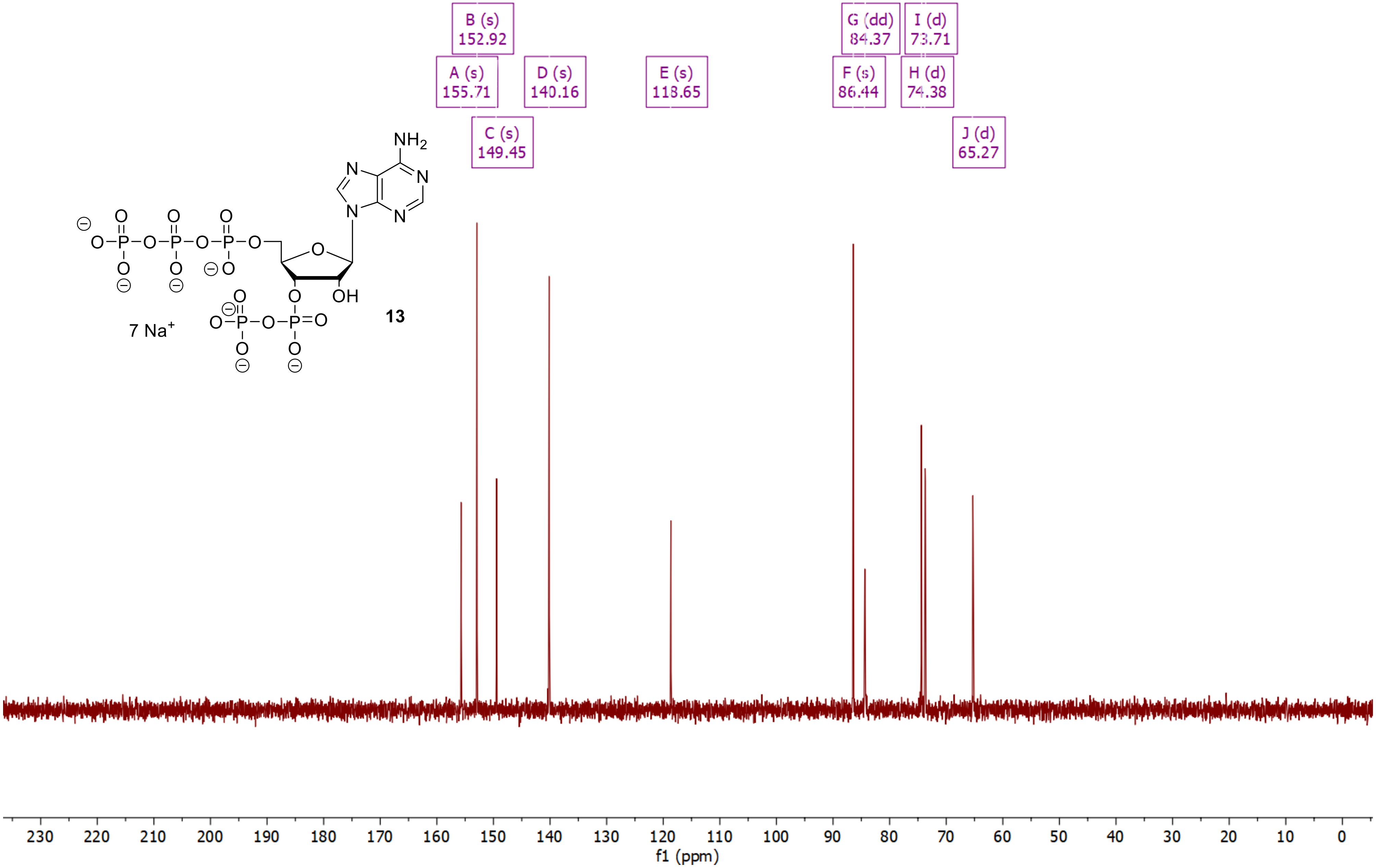

Compound **13** (pppApp), $^{13}C[^{1}H]$-NMR (D_2O, 101 MHz), detailed

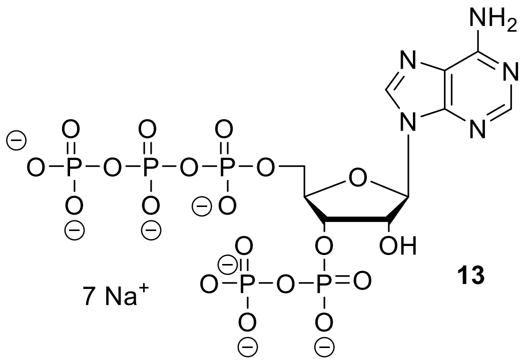

13

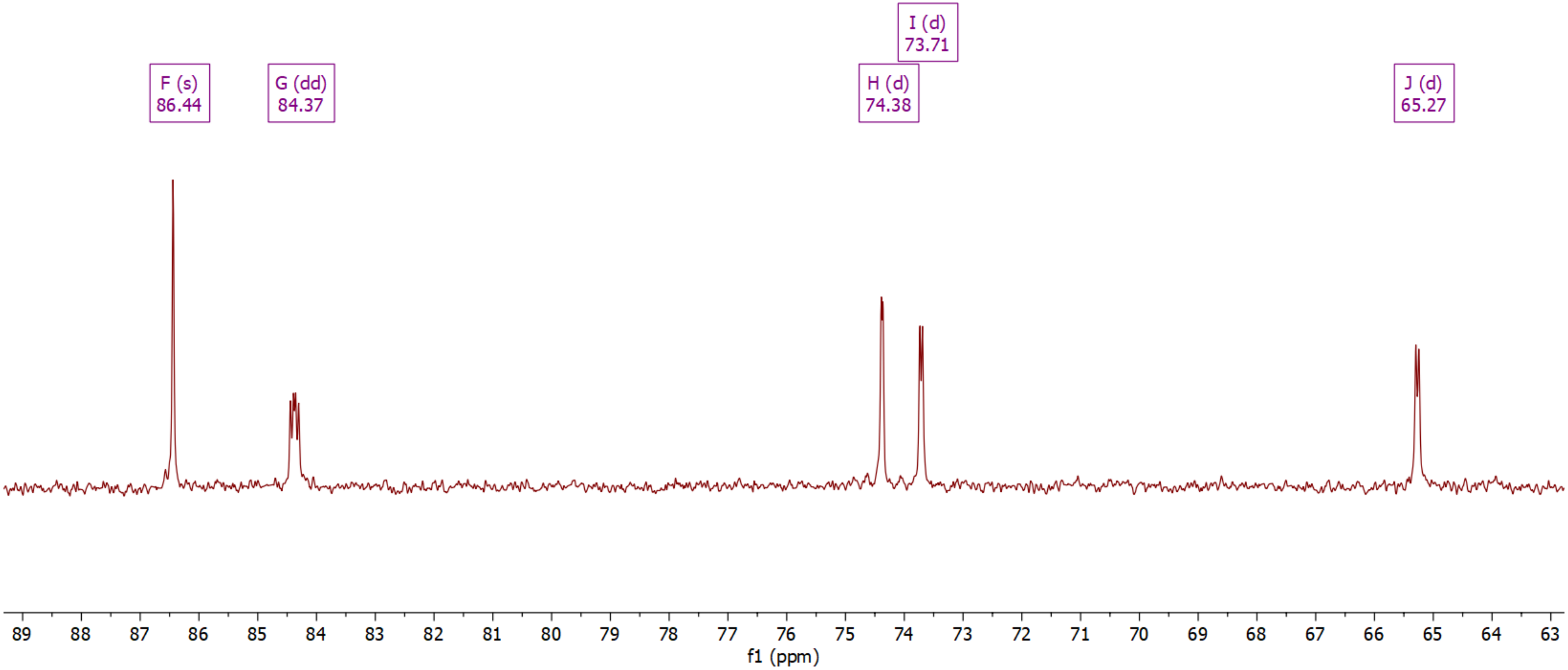

Compound **12** (pppGpp), ^{1}H-NMR (D_2O, 400 MHz)

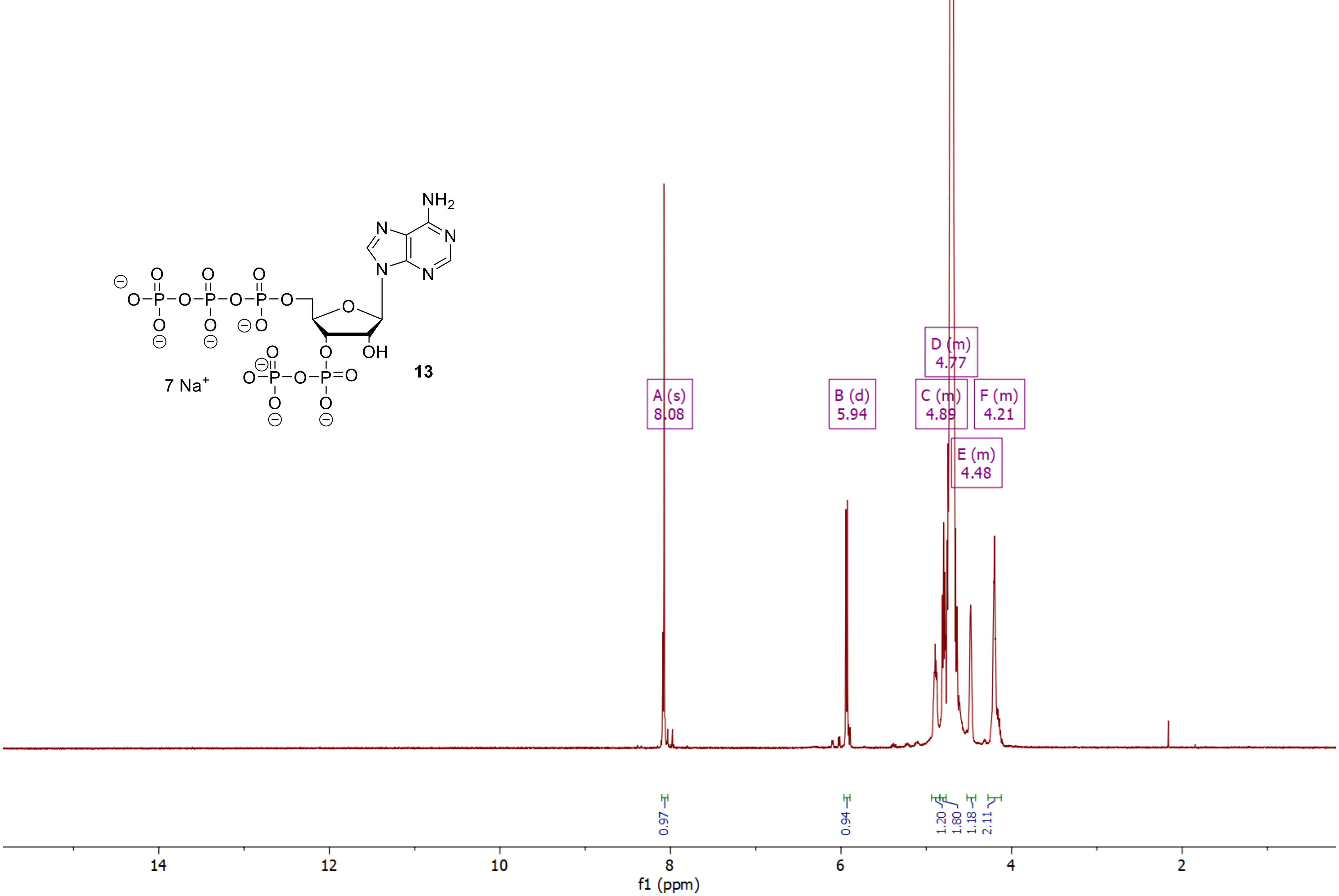

Compound **12** (pppGpp), ed-HSQC (D_2O)

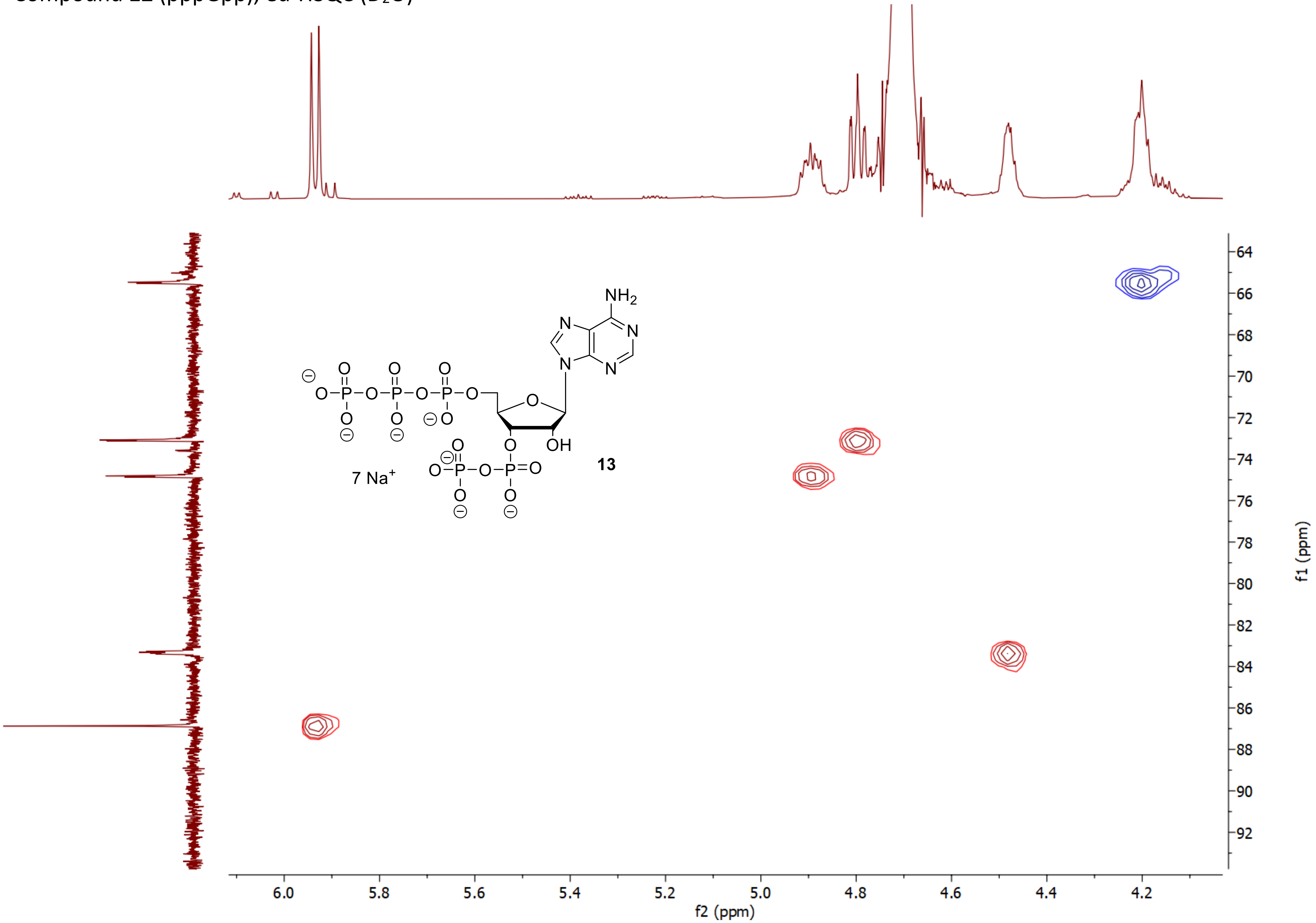

Compound **12** (pppGpp), $^{31}P[^{1}H]$-NMR (D_2O, 162 MHz)

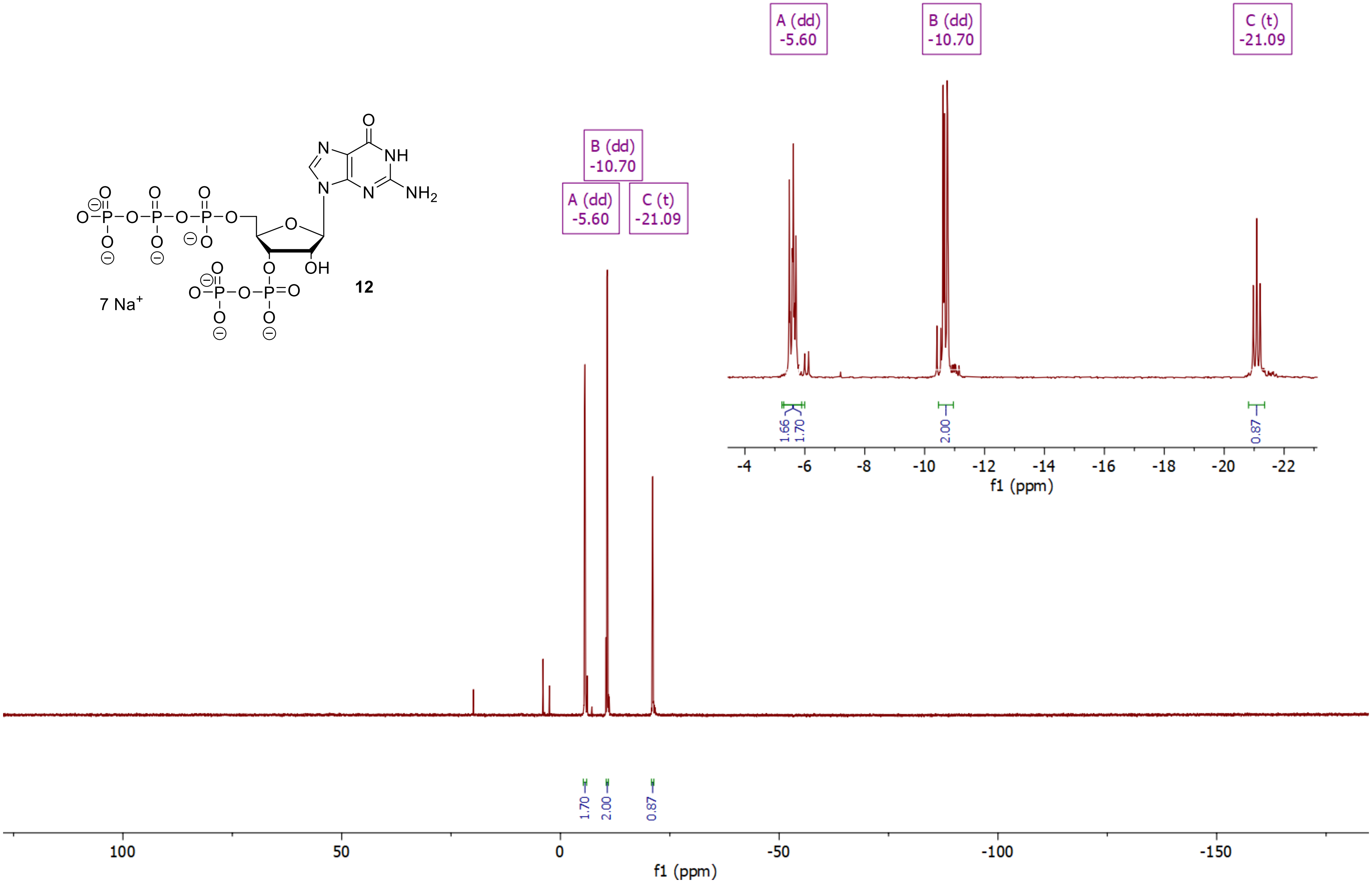

Compound **12** (pppGpp), $^{13}C[^{1}H]$-NMR (D_2O, 101 MHz)

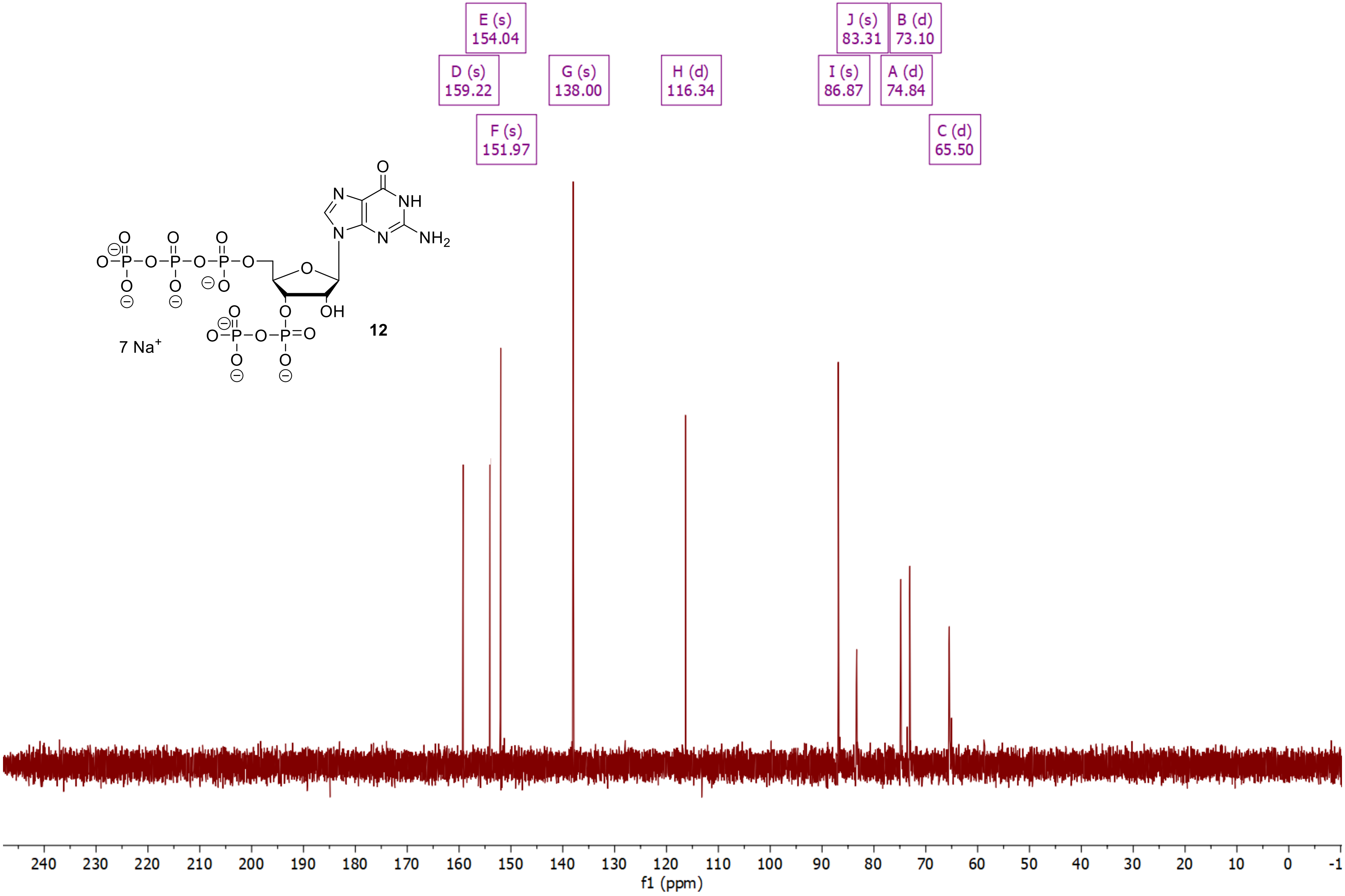

Compound **16** (pentinyl-pppAp), ^{1}H-NMR (D_2O, 400 MHz)

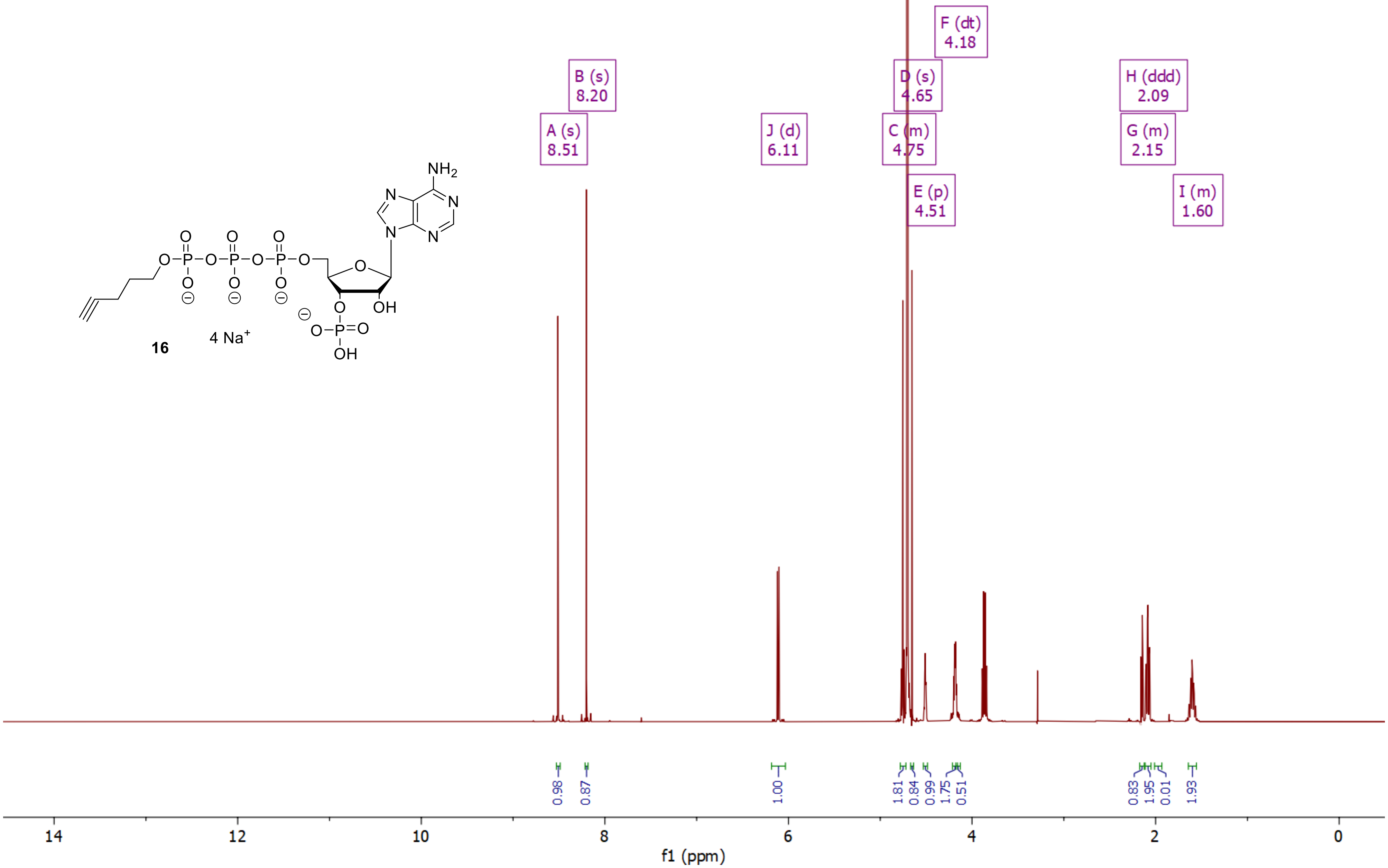

Compound **16** (pppApp), $^{31}P[^{1}H]$-NMR (D_2O, 162 MHz)

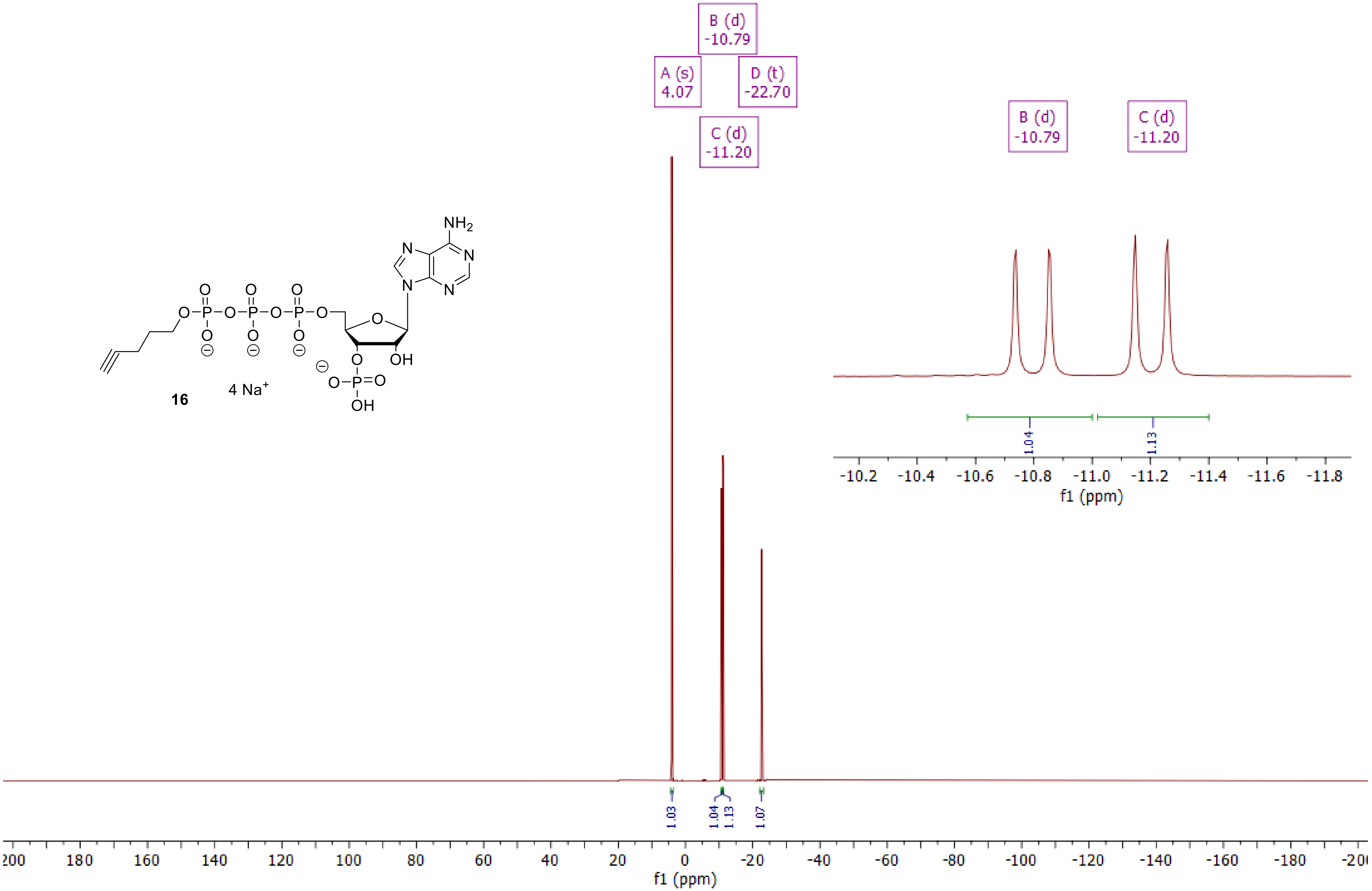

Compound **16** (pppAp), ^{31}P-NMR (D_2O, 162 MHz)

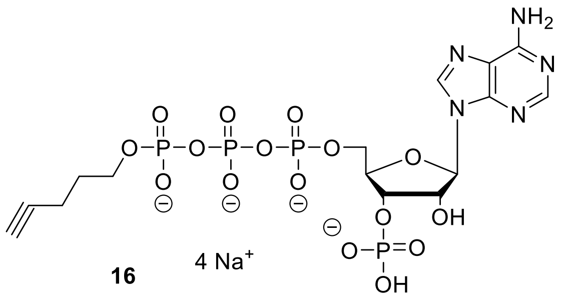

16

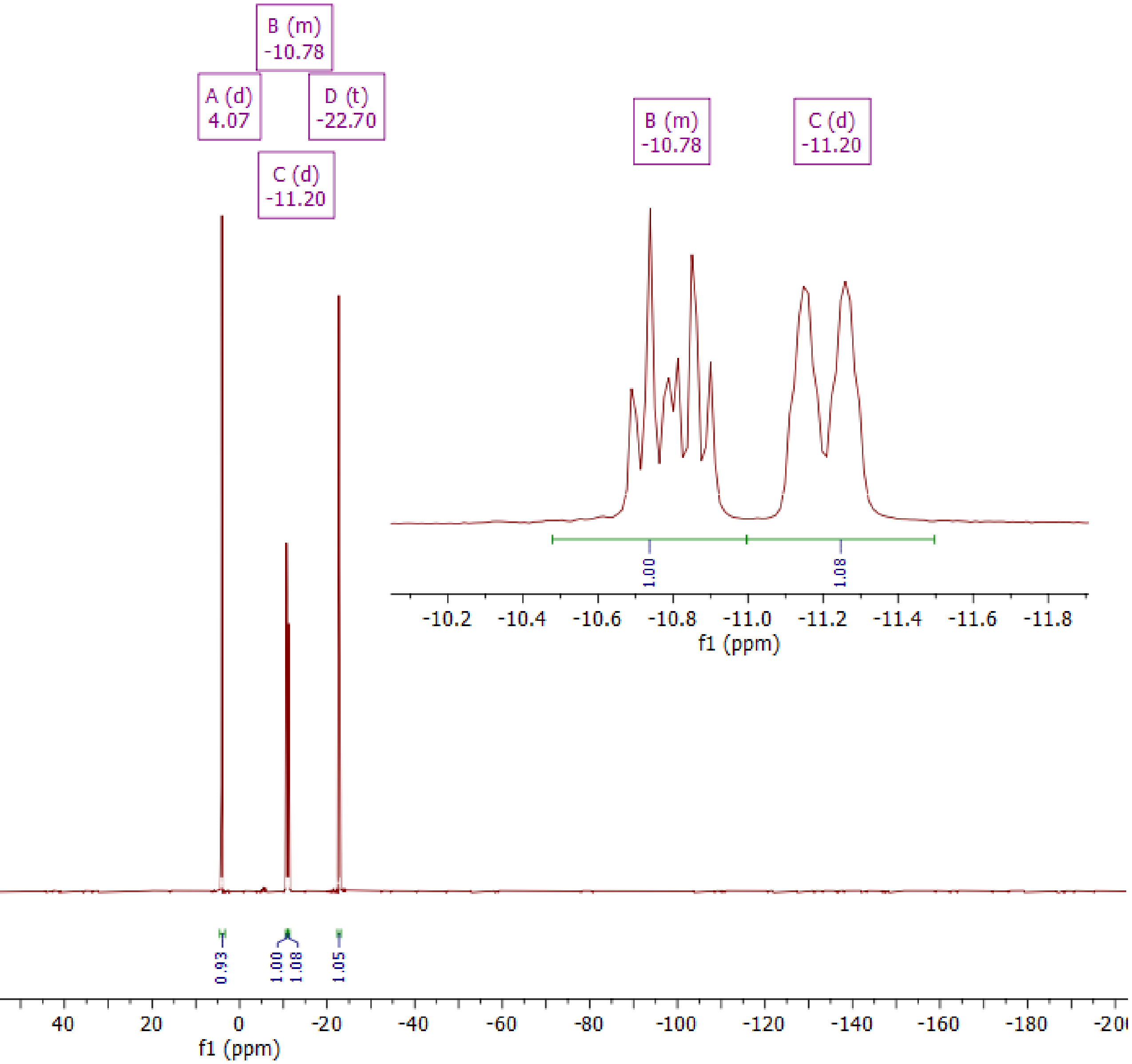

Compound **16** (pentinyl-pppAp), $^{13}C[^{1}H]$-NMR (D_2O, 101 MHz)

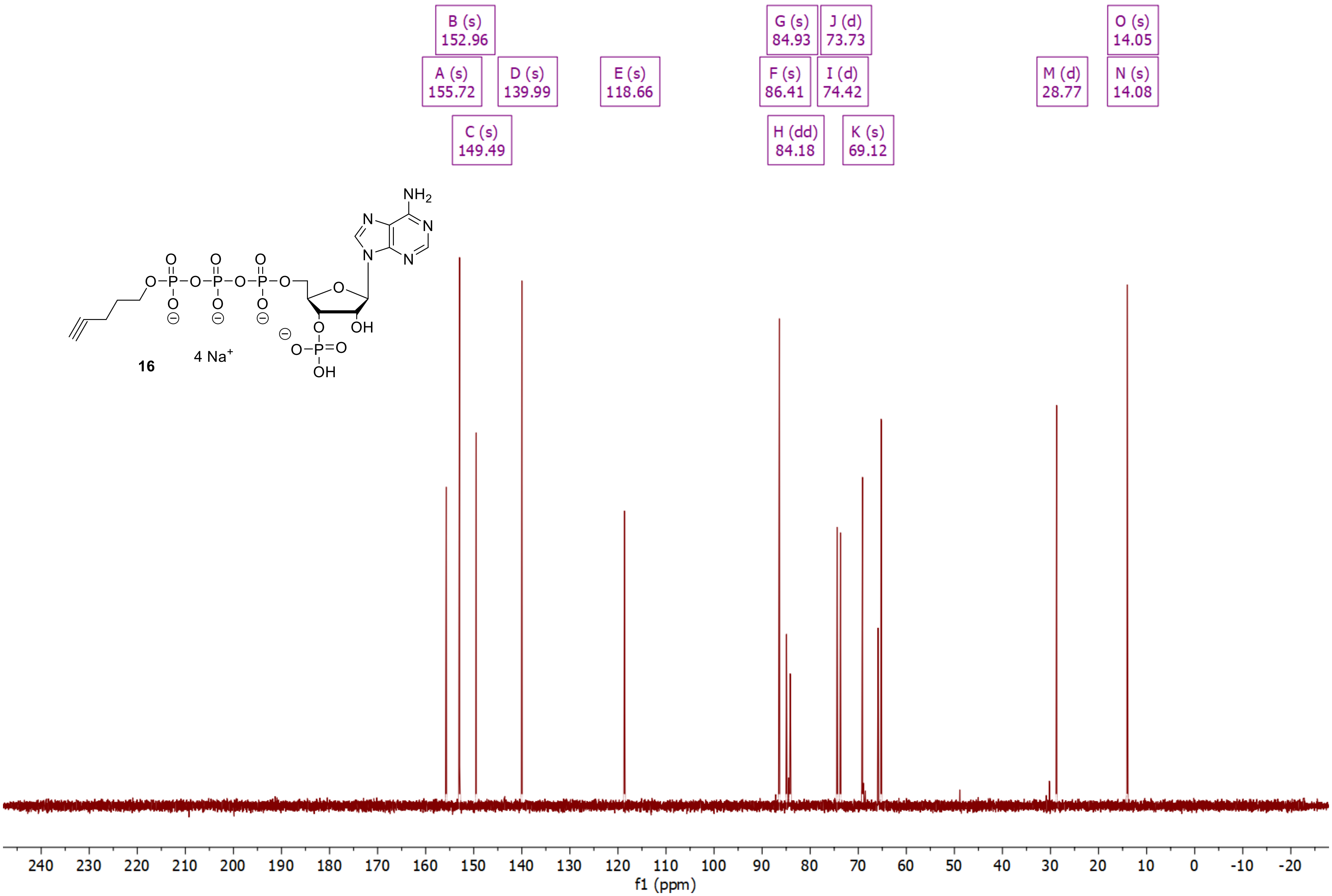

Compound **16** (pentynyl-pppAp), $^{13}C[^{1}H]$-NMR (D_2O, 101 MHz), detailed

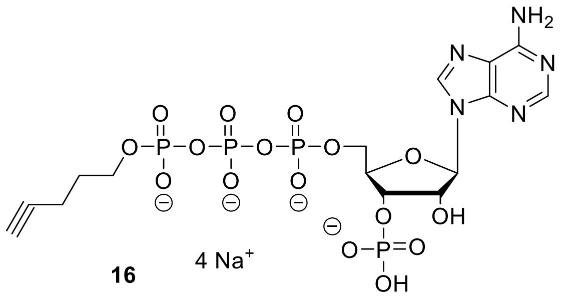

16

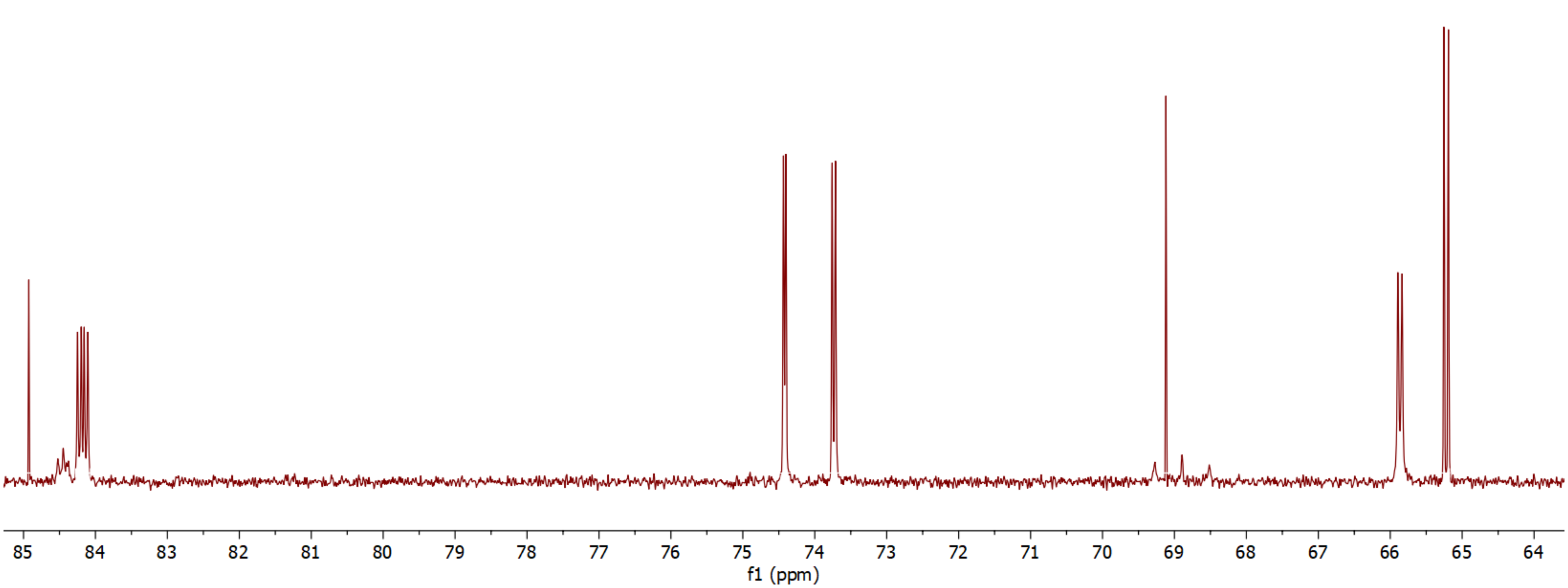

Compound **17** (pentinyl-pppApp), ^{1}H-NMR (D_2O, 400 MHz)

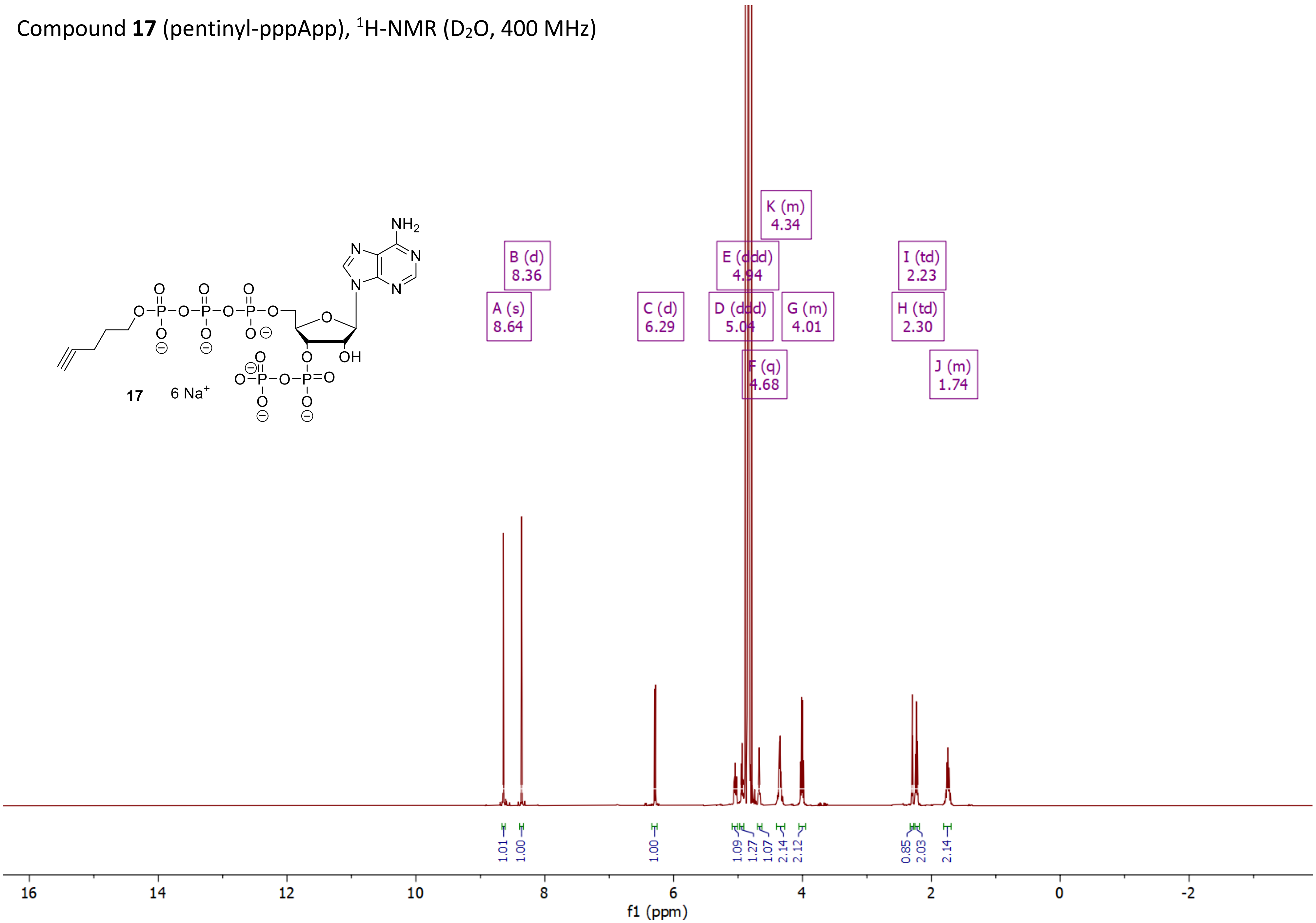

Compound **17** (pentynyl-pppApp), $^{31}P[^{1}H]$-NMR (D_2O, 162 MHz)

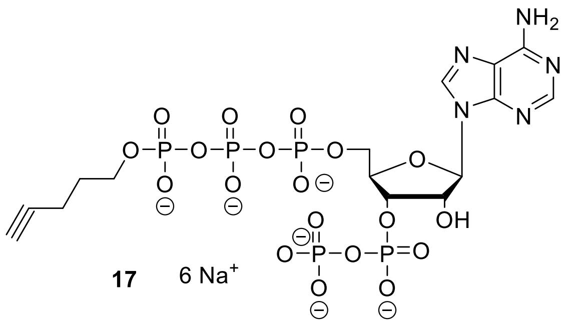

17

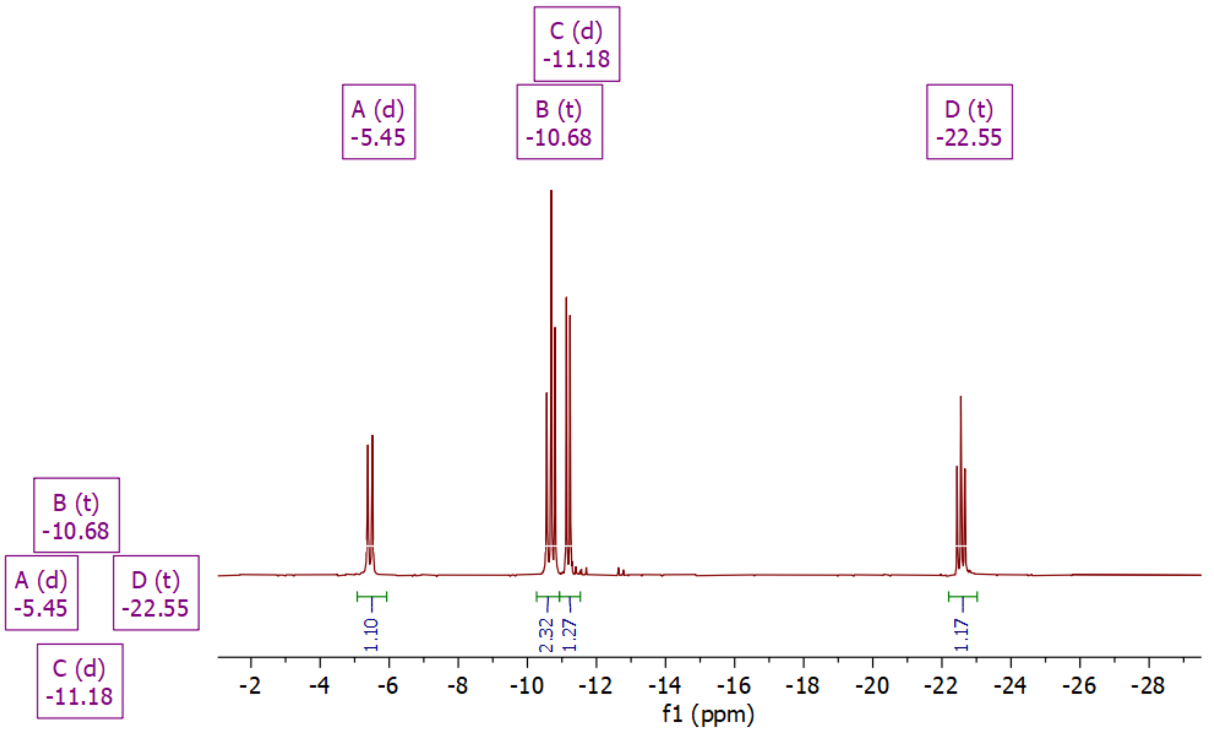

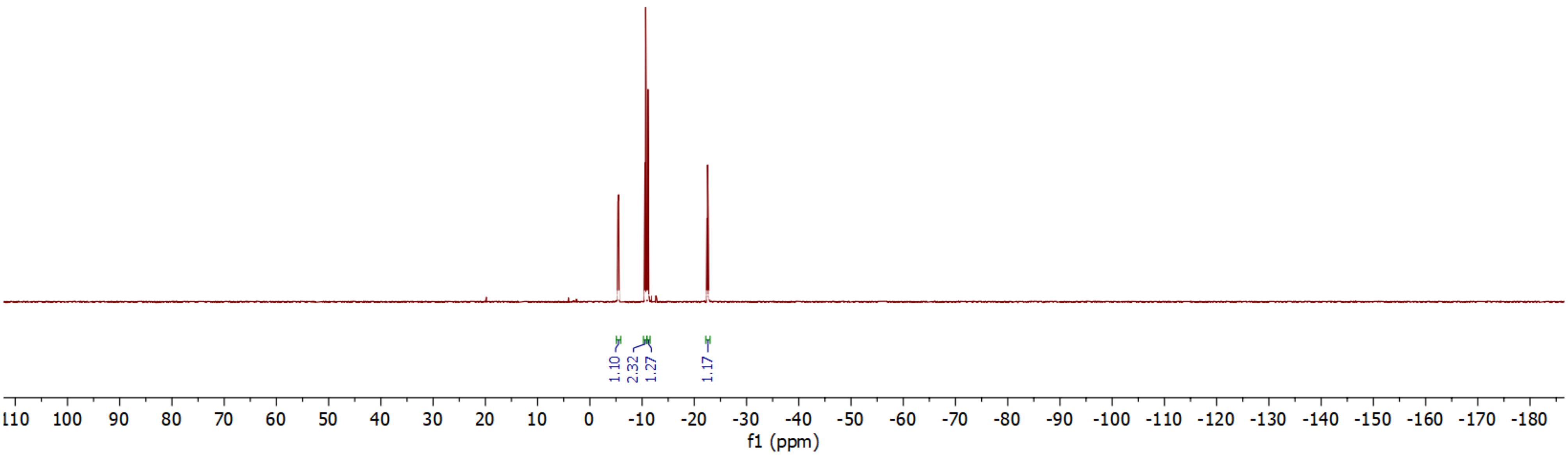

Compound **17** (pentynyl-pppApp), $^{13}C[^{1}H]$-NMR (D_2O, 101 MHz)

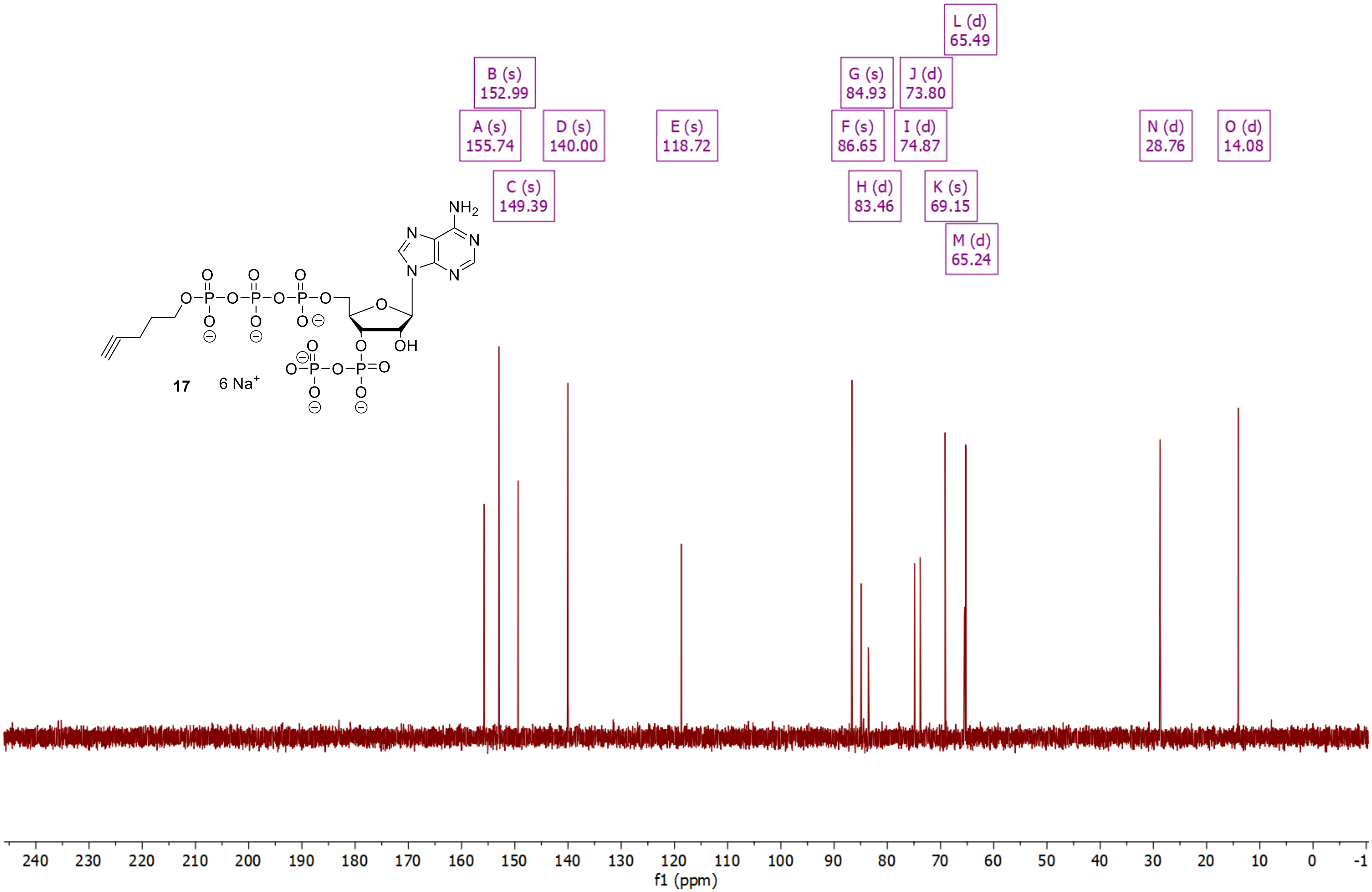

Compound **18** (amino-pppApp), ^{1}H-NMR (D_2O, 400 MHz)

18

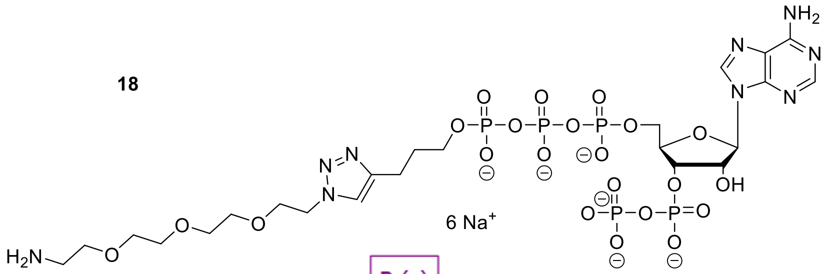

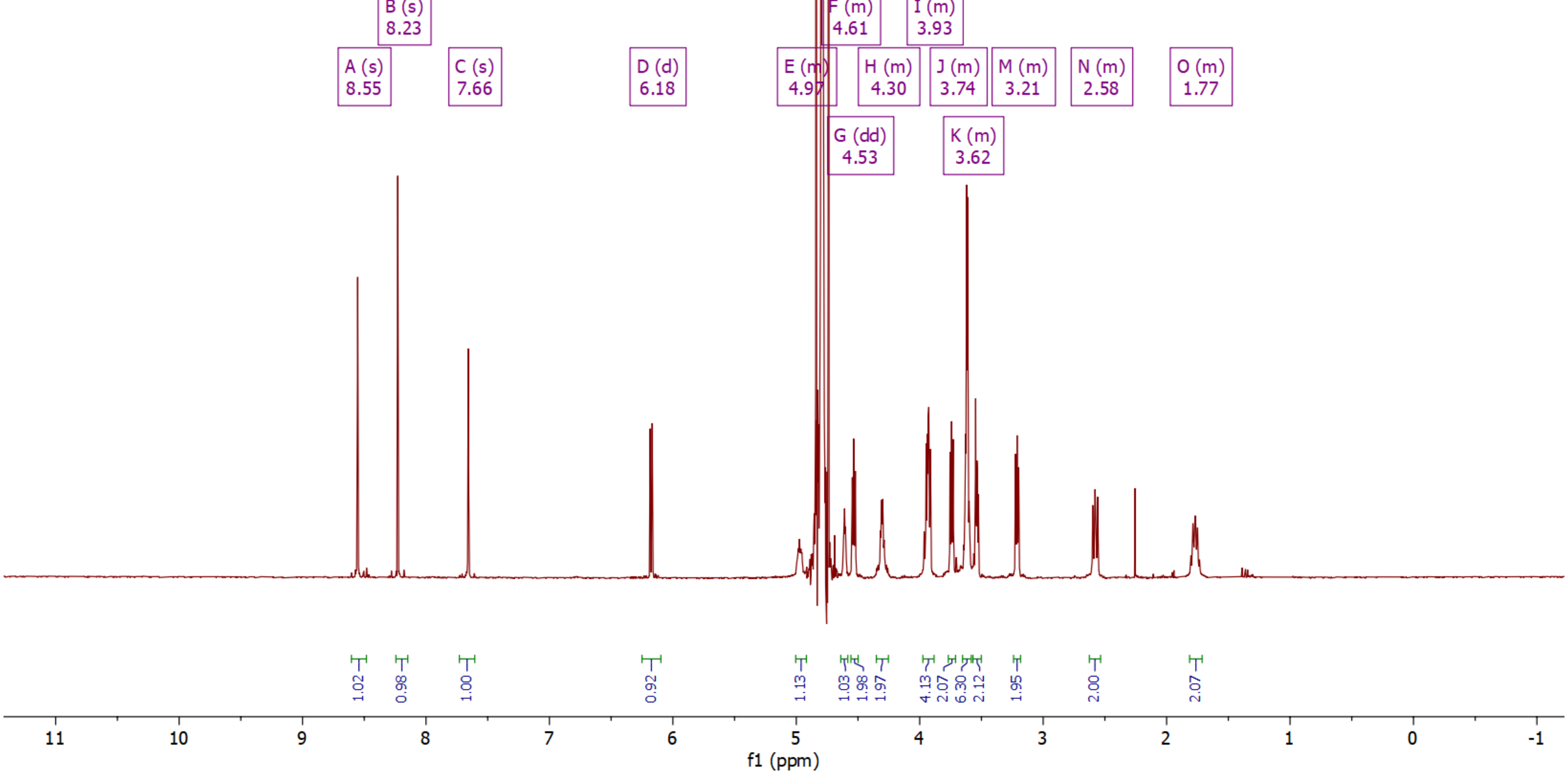

Compound **18** (amino-pppApp), $^{31}P[^{1}H]$-NMR (D_2O, 162 MHz)

18

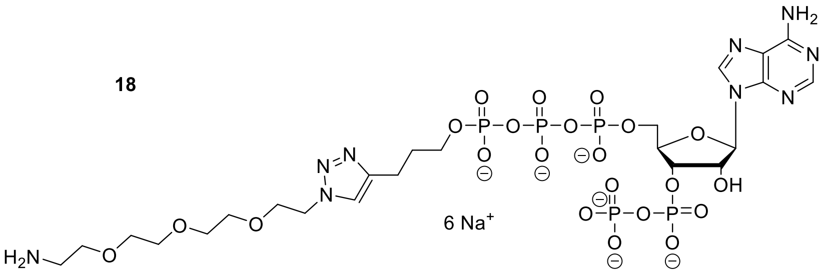

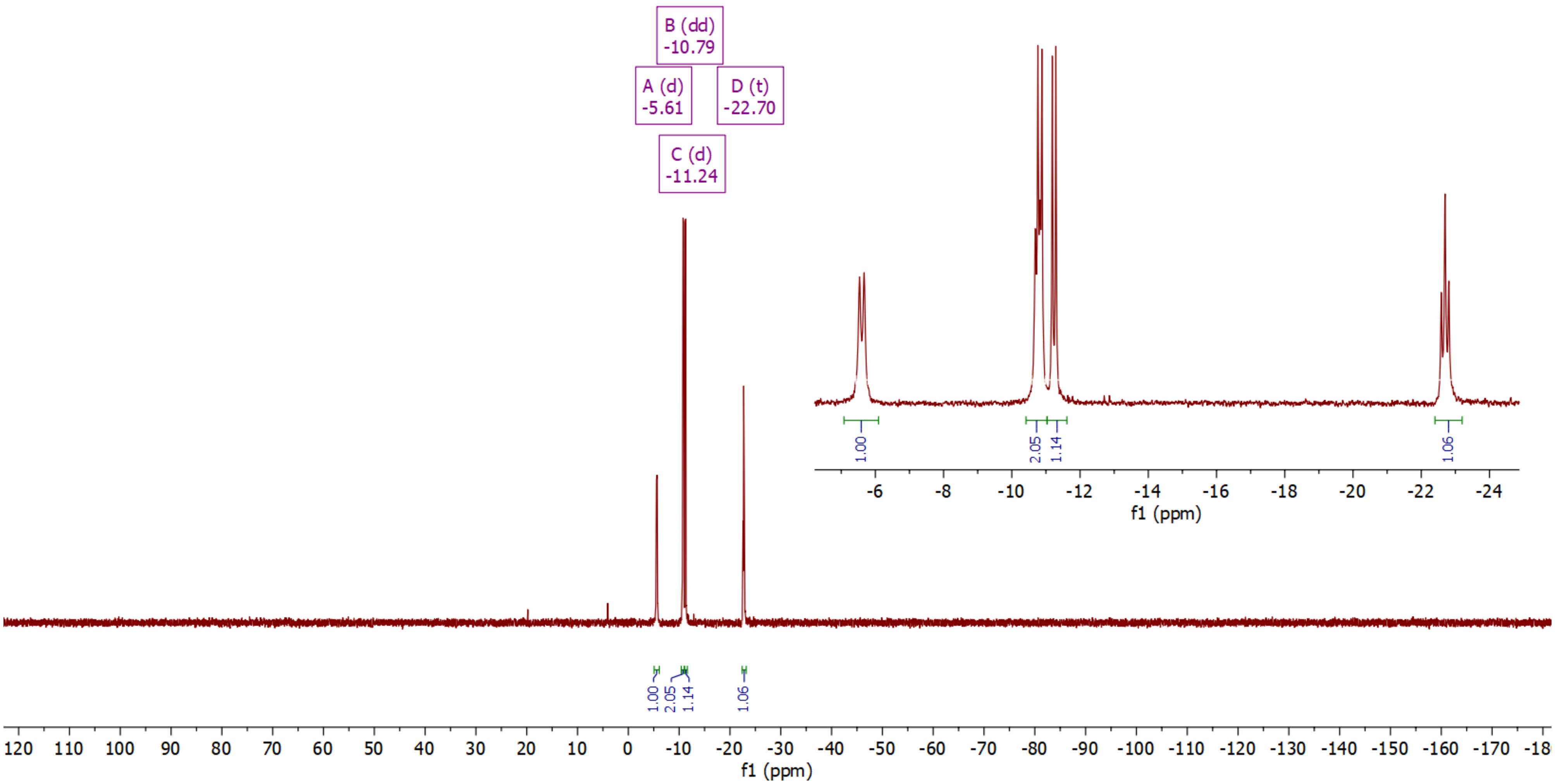

Compound **18** (amino-pppApp), $^{13}C[^{1}H]$-NMR (D_2O, 101 MHz)

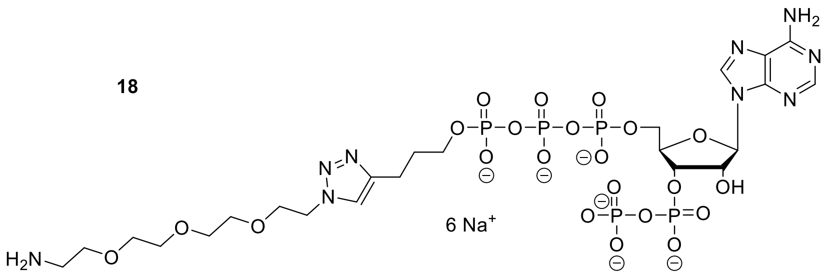

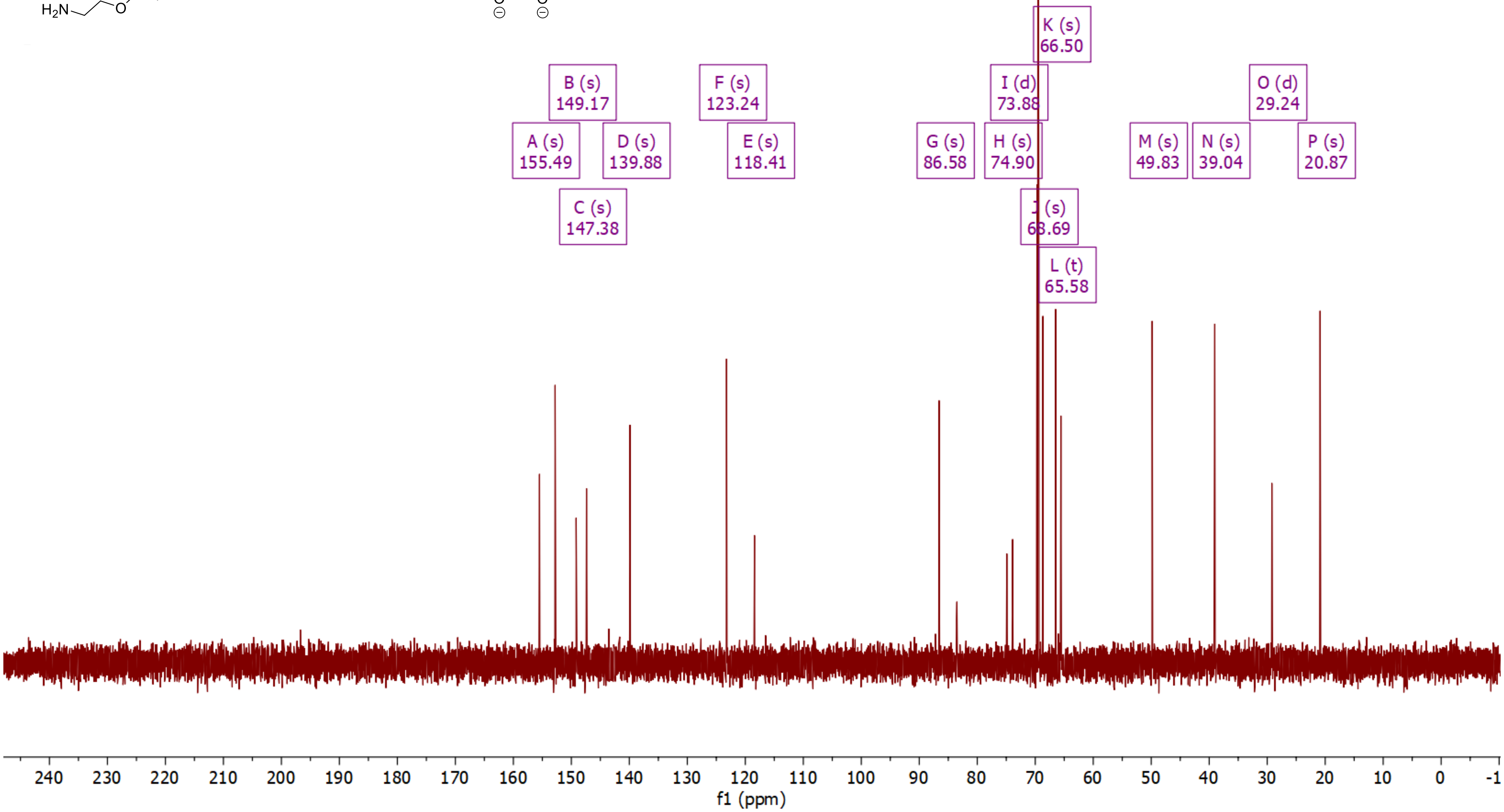

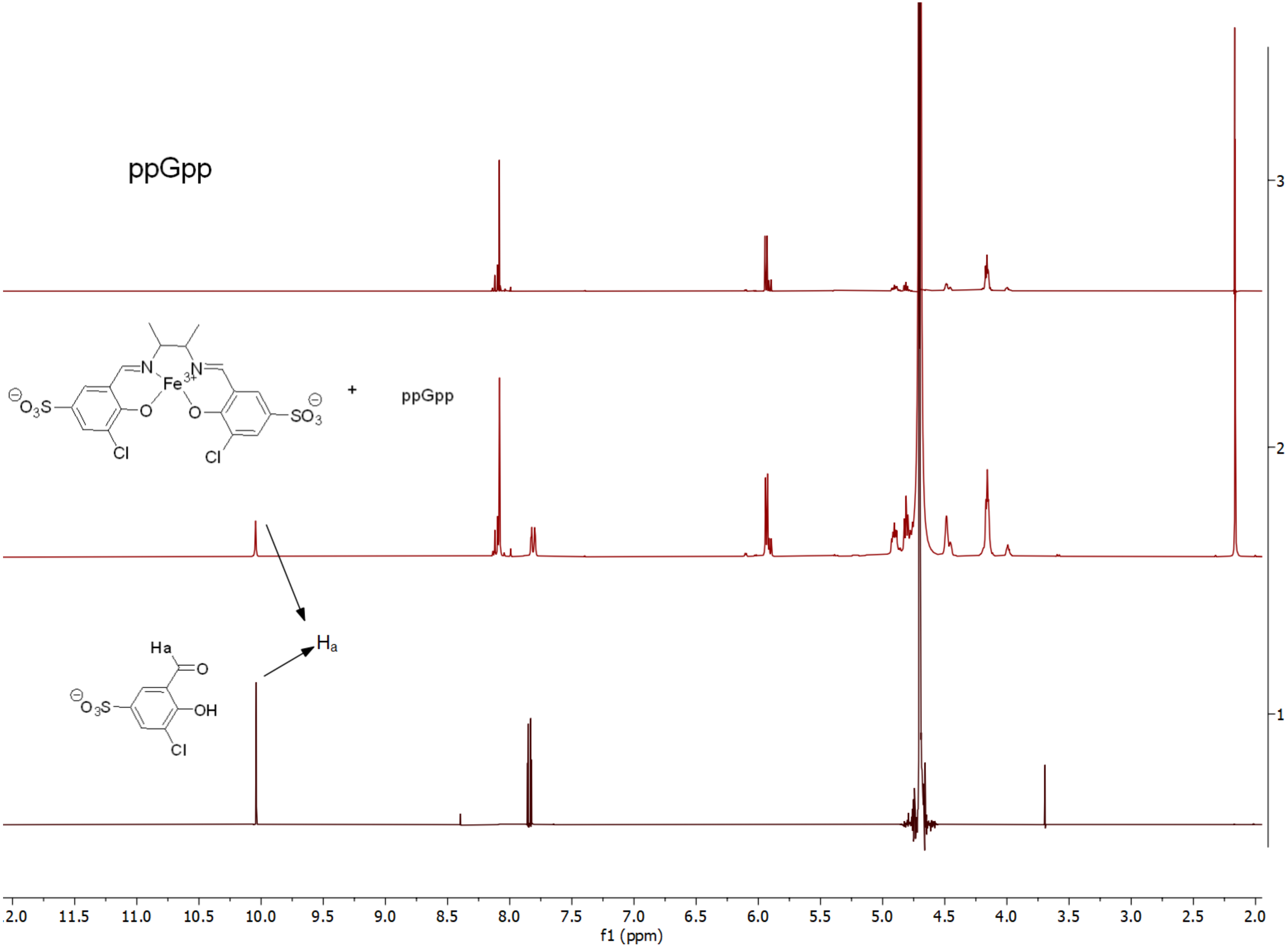

ppGpp
Fe3+
N
N
O
O
SO3
O3S
Cl
Cl
+
ppGpp
Ha
O
OH
O3S
Cl
Ha
3
2
1
2.0
11.5
11.0
10.5
10.0
9.5
9.0
8.5
8.0
7.5
7.0
6.5
6.0
5.5
5.0
4.5
4.0
3.5
3.0
2.5
2.0
f1 (ppm)

7. HRMS analysis

HRMS (ESI) Analysis of compound 16: PpppAp

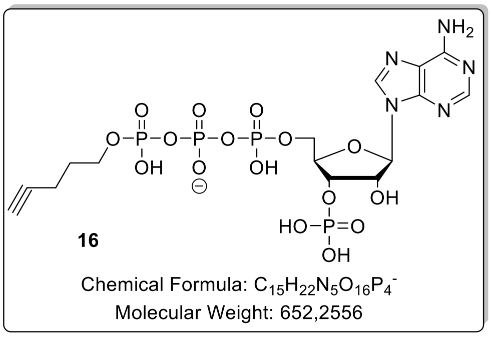

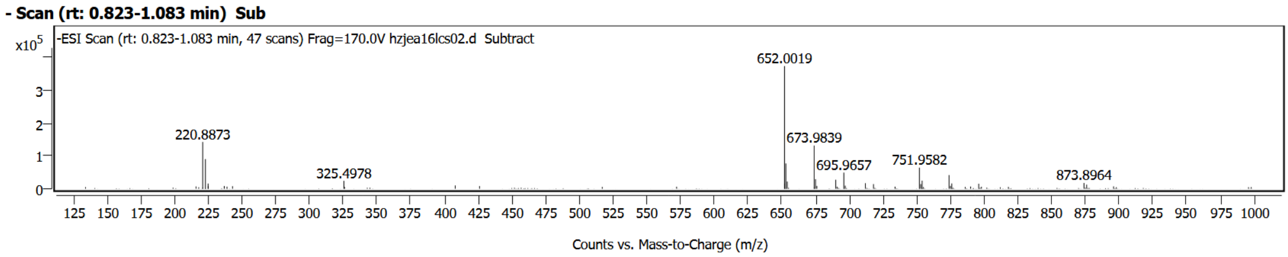

HRMS (ESI) Analysis of compound 17: PpppApp

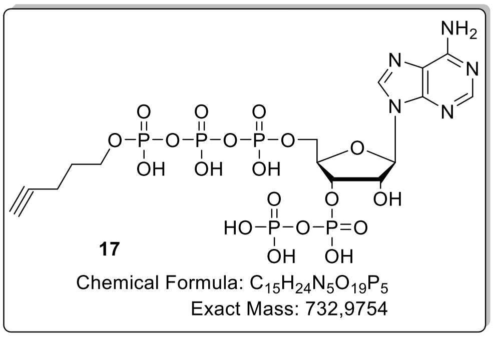

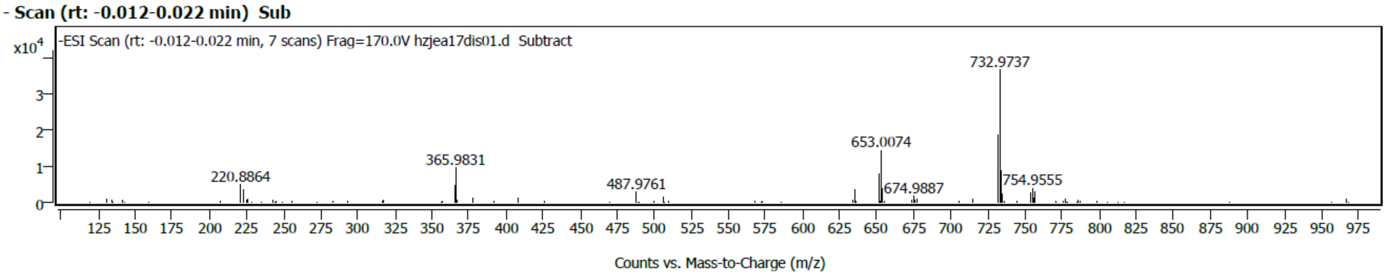

HRMS (ESI) Analysis of compound 18: ApppApp

18

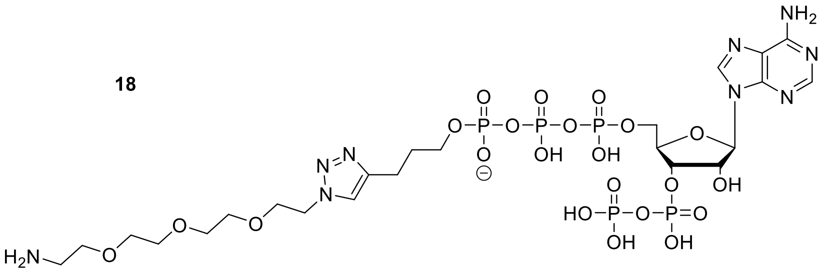

Chemical Formula: $C_{23}H_{41}N_9O_{22}P_5^-$

Exact Mass: 950,1060

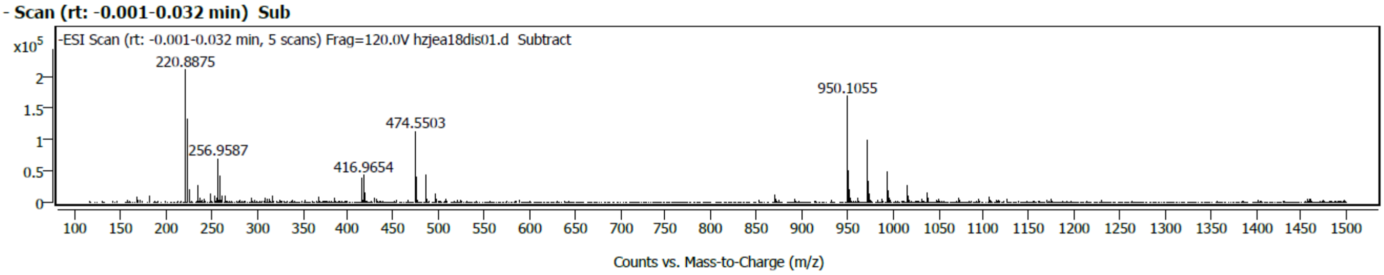

HRMS (ESI) Analysis of compound 8: ppAp

8

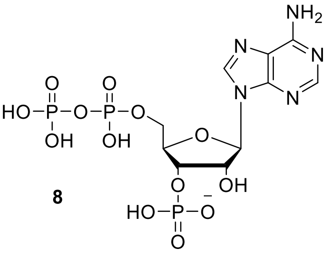

Chemical Formula: $C_{10}H_{15}N_5O_{13}P_3^-$

Exact Mass: 505,9885

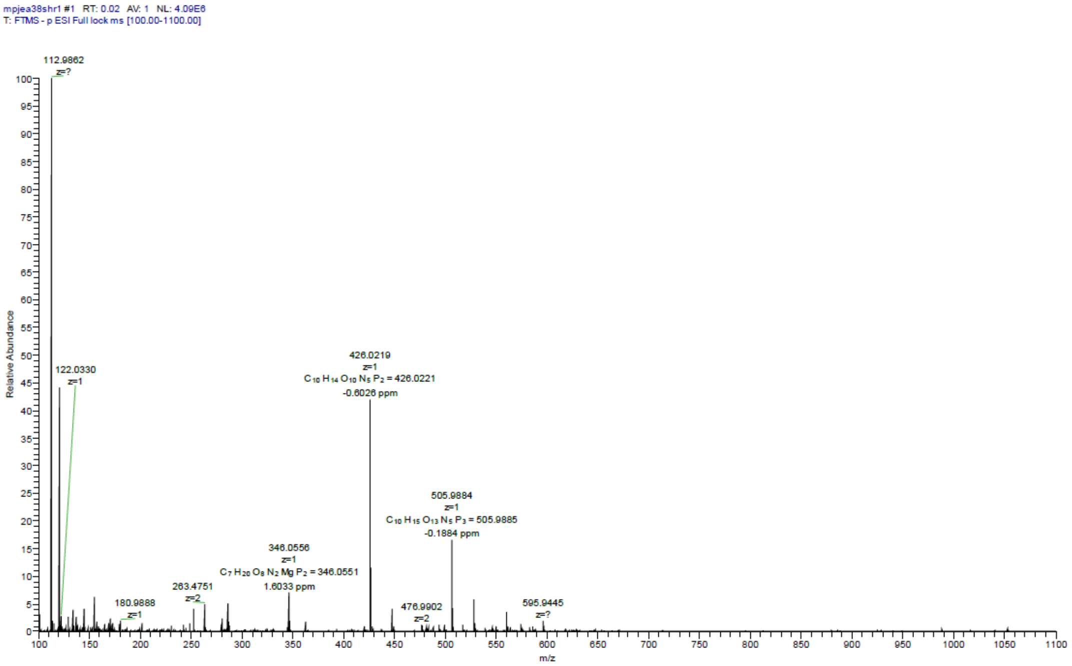

HRMS (ESI) Analysis of compound 11: ppGp

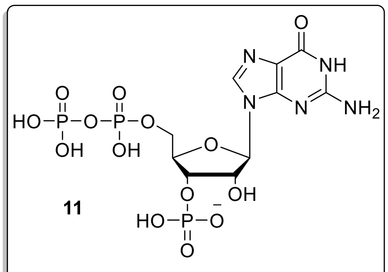

Chemical Formula: $C_{10}H_{15}N_5O_{14}P_3^-$
Exact Mass: 521,9834

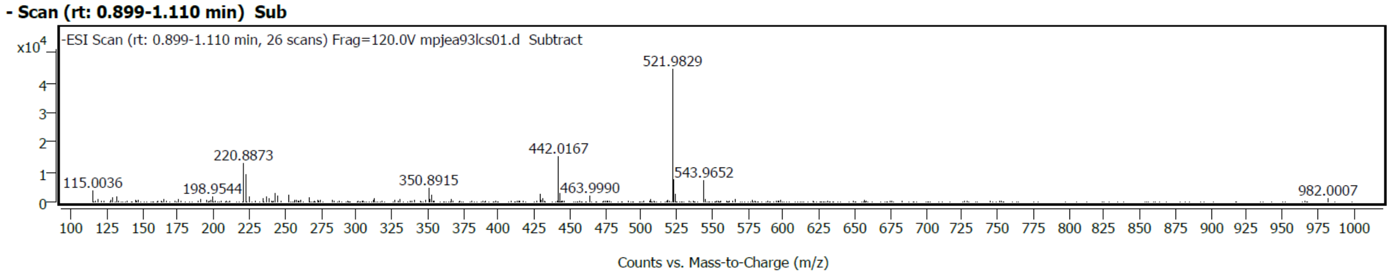

HRMS (ESI) Analysis of compound 13: pppApp

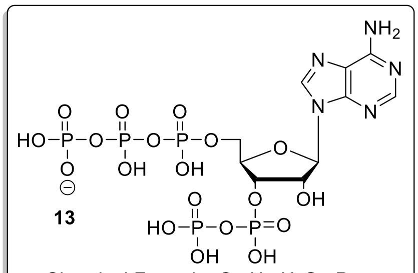

Chemical Formula: $C_{10}H_{17}N_5O_{19}P_5^-$
Exact Mass: 665,9211

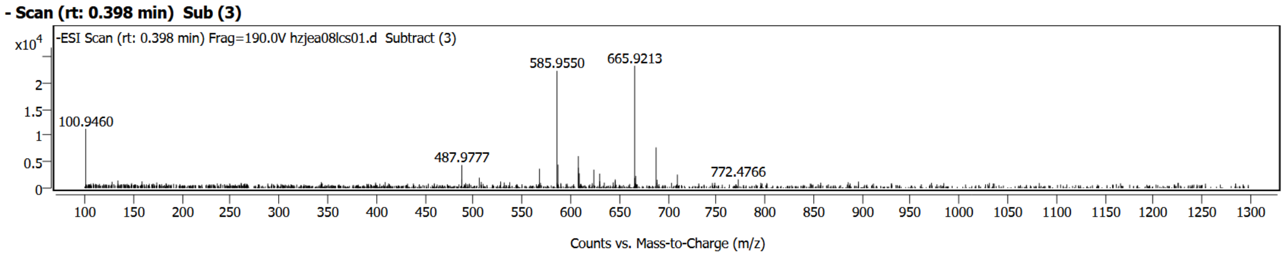

HRMS (ESI) Analysis of compound 12: pppGpp

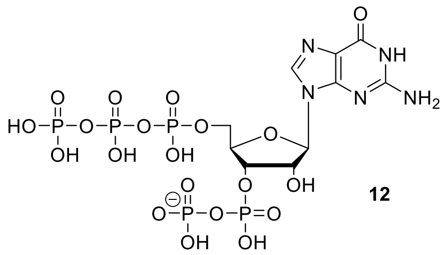
Chemical Formula: $C_{10}H_{17}N_5O_{20}P_5^-$
Exact Mass: 681,9160

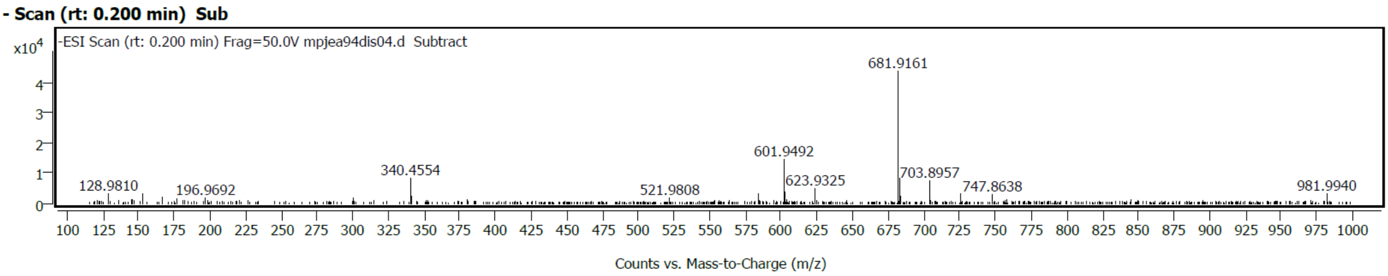

References

1 L. Bialy and H. Waldmann, *Angew. Chem. Int. Ed.*, 2002, **41**, 1748.

2 A. Hofer, G. S. Cremosnik, A. C. Müller, R. Giambruno, C. Trefzer, G. Superti-Furga, K. L. Bennett and H. J. Jessen, *Chemistry (Weinheim an der Bergstrasse, Germany)*, 2015, **21**, 10116.

3 T. M. Haas, P. Ebensperger, V. B. Eisenbeis, C. Nopper, T. Dürr, N. Jork, N. Steck, C. Jessen-Trefzer and H. J. Jessen, *Chemical communications (Cambridge, England)*, 2019, **55**, 5339.

4 J. Singh, A. Ripp, T. M. Haas, D. Qiu, M. Keller, P. A. Wender, J. S. Siegel, K. K. Baldridge and H. J. Jessen, *Journal of the American Chemical Society*, 2019, **141**, 15013.

5 P. J. L. M. Quaedflieg, A. P. van der Heiden, L. H. Koole, A. J. J. M. Coenen, S. van der Wal and E. M. Meijer, *J. Org. Chem.*, 1991, **56**, 5846.

6 A. Ripp, M. Krämer, V. Barth, P. Moser, T. M. Haas, J. Singh, T. Huck, L. Gleue, K. Friedland, M. Helm and H. J. Jessen, *Angew. Chem. Int. Ed.*, 2025, **64**, e202414537.

7 N. Kumari, H. Huang, H. Chao, G. Gasser and F. Zelder, *Chembiochem : a European journal of chemical biology*, 2016, **17**, 1211.

8 M. J. Frisch, G. W. Trucks and D. J. Fox, Gaussian 16 (Revision C.01), Gaussian, Inc., Wallingford, CT, 2019.

9 A. D. Becke, *The Journal of Chemical Physics*, 1993, **98**, 5648.

10 F. Weigend and R. Ahlrichs, *Physical chemistry chemical physics : PCCP*, 2005, **7**, 3297.

11 M. Dolg, U. Wedig, H. Stoll and H. Preuss, *The Journal of Chemical Physics*, 1987, **86**, 866.

12 J. Tomasi, B. Mennucci and R. Cammi, *Chemical reviews*, 2005, **105**, 2999.

13 S. Grimme, S. Ehrlich and L. Goerigk, *Journal of computational chemistry*, 2011, **32**, 1456.
